# Synthesis of New Morphinan Opioids by TBADT‐Catalyzed Photochemical Functionalization at the Carbon Skeleton**

**DOI:** 10.1002/chem.202201478

**Published:** 2022-07-13

**Authors:** Dmitry Gorbachev, Elliot Smith, Stephen P. Argent, Graham N. Newton, Hon Wai Lam

**Affiliations:** ^1^ The GlaxoSmithKline Carbon Neutral Laboratories for Sustainable Chemistry University of Nottingham Jubilee Campus, Triumph Road Nottingham NG7 2TU UK; ^2^ School of Chemistry University of Nottingham University Park Nottingham NG7 2RD UK

**Keywords:** late-stage functionalization, morphinans, opioids, photocatalysis, radical reactions

## Abstract

The synthesis of new morphinan opioids by the addition of photochemically generated carbon‐centered radicals to substrates containing an enone in the morphinan C‐ring, is described. Using tetrabutylammonium decatungstate (TBADT) as a hydrogen atom transfer photocatalyst, diverse radical donors can be used to prepare a variety of C8‐functionalized morphinan opioids. This work demonstrates the late‐stage modification of complex, highly functionalized substrates.

## Graphical abstract

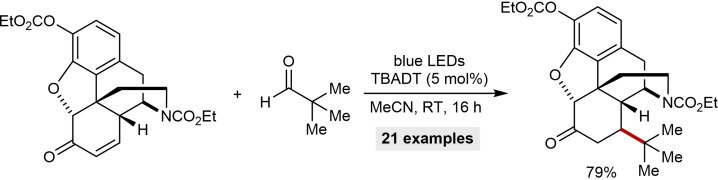

## Introduction

**Figure 1:**
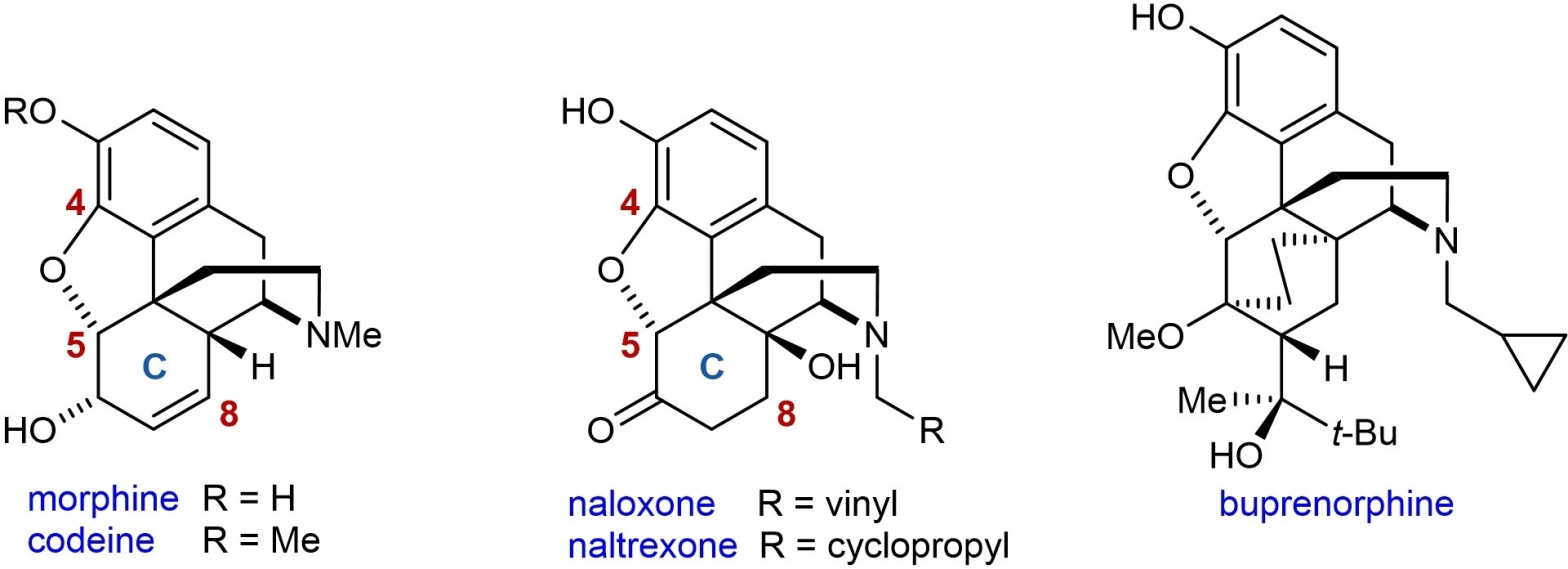
Representative morphinan opioids.

Morphinan opioids (Figure 1 shows representative examples), typified by its most well‐known member morphine,^[1]^ are important medicines for the treatment of pain and other disorders because of their effect on the opioid receptors in the central nervous system. However, many opioids possess significant side effects that include respiratory depression, sedation, and constipation. They can be highly addictive, which leads to abuse and significant numbers of overdose deaths.[^1d^, ^1e^, ^2^] Therefore, there has been a long‐standing quest to develop new opioids that retain beneficial therapeutic qualities but with reduced side effects and addictive properties. Extensive research in this area has resulted in the preparation of numerous derivatives, a good understanding of structure‐activity relationships (SAR),[^1e^, ^3^] and a range of compounds used clinically today.

To date, morphinan opioid SAR studies have investigated the nature of the nitrogen substituent (a methyl group in morphine), which is crucial in determining the biological activity, as well as most regions of the 4,5‐epoxymorphinan scaffold.^[3]^ However, there has been relatively little investigation of C8‐substituted derivatives, which are typically prepared from morphinans containing an enone in the C‐ring (see Figure 1 for numbering and labeling).^[4]^ Kotick and co‐workers have reported various functionalizations of morphinan C‐ring enones, such as 1,4‐additions of organometallic reagents, cyclopropanation, and epoxidation, to prepare a range of C8‐substituted derivatives, some of which have interesting biological activities.[^4b^, ^4c^, ^4d^, ^4e^] A limited number of other reports have described the preparation of C8‐substituted derivatives.[^4a^, ^4f^, ^4g^, ^4h^, ^4i^, ^4j^]

The advent of modern methods for the late‐stage functionalization^[5]^ of complex molecules opens up new opportunities to access novel derivatives that previously would have been challenging to prepare, and which could have interesting biological properties. In this context, photochemical hydrogen atom transfer (HAT) catalysis^[6]^ has recently emerged as a powerful tool for the late‐stage functionalization^[5]^ of complex molecules. Using a HAT photocatalyst, mild and selective C−H functionalization of diverse substrates can be achieved through the generation of carbon‐centered radicals, obviating the need for prefunctionalized starting materials.^[6]^ We questioned whether the 1,4‐addition of nucleophilic radicals generated by HAT to morphinan opioids containing an enone in the C‐ring would constitute a versatile method to produce C8‐substituted derivatives, complementary to those described previously.^[4]^ To our knowledge, no prior study has focused on this approach, with the only related precedent being the 1,4‐addition of photochemically generated THF radicals to two morphinan enones, as part of a larger investigation into the photochemistry of structurally modified morphine alkaloids.^[4g]^ Herein, we describe the successful execution of this strategy.

## Results and Discussion

**Figure 2:**
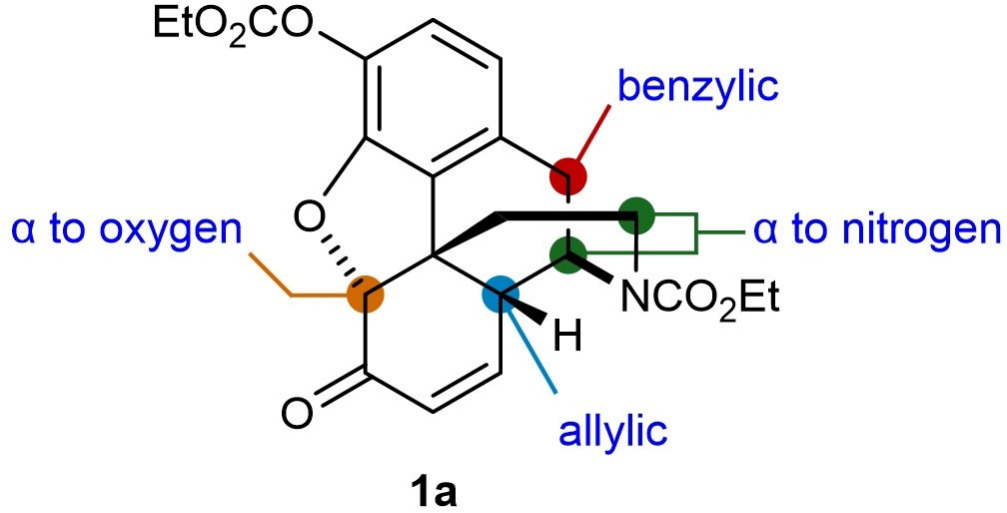
C−H bonds in morphinan opioid **1 a** with potential HAT reactivity.

This study began with attempts to functionalize enone **1 a** with carbon‐centered radicals using tetra‐*n*‐butylammonium decatungstate (TBADT) as the HAT photocatalyst (Figure 2).[^7^, ^8^] TBADT is one of the most versatile HAT photocatalysts, being able to generate radicals from C−H bonds at benzylic and allylic positions, α‐ to heteroatoms, and formyl groups, as well as at secondary or tertiary C−H bonds of simple alkyl groups.^[7]^ However, we were mindful that this very same versatility could present challenges of functional group incompatibility with a highly functionalized substrate such as **1 a**, which contains several different types of C−H bonds that could also potentially react with TBADT (Figure 2). Nevertheless, investigating these issues of functional group tolerance are exactly the types of studies required to inform greater application of methods for late‐stage modification of highly functionalized molecules.

An investigation of reaction conditions established that irradiation of a solution of **1 a** and 3‐phenylpropanal^[9]^ (5.0 equiv.) in MeCN (0.4 M concentration) in the presence of TBADT (5 mol%) at room temperature under argon for 16 h with blue LEDs gave the 1,4‐addition product **2 aa** in 67 % isolated yield (Table 1, entry 1).^[10]^ The setting employed for the blue LED source has an emission spectrum with a small peak at ca. 390 nm (see Supporting Information for details). Changing the light source to a UV LED lamp (set at 370 nm, see Supporting Information for details) reduced the yield to 54 % (Table 1, entry 2). Reducing the catalyst loading, reaction time, or concentration had detrimental effects on the yield (Table 1, entries 3–5). No reaction was observed in the absence of TBADT (Table 1, entry 6). Increasing the quantity of 3‐phenylpropanal to 10.0 equiv. increased the yield to 74 % (Table 1, entry 7), while using 2.0 equiv. resulted in a decreased yield of 43 % (Table 1, entry 8). A reaction conducted under an air atmosphere gave essentially identical results (entry 9) to that conducted under argon (Table 1, entry 1). The inclusion of H_2_O (10 equiv.) had a negative effect on the yield (Table 1, entry 10), while other HAT photocatalysts such as eosin Y[^11^, ^12^] and anthraquinone^[13]^ were ineffective, leading to significant recovered starting materials (Table 1, entries 11–13).

**Table 1:** Evaluation of reaction conditions.^[a]^

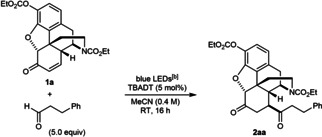		
Entry	Deviation from Conditions	Yield of **2 aa** [%]^[c]^
1	None	67
2	UV lamp (370 nm)	54
3	1 mol% TBADT	32
4	Reaction time of 5 h	28
5	Concentration of 0.1 M	54
6	No TBADT	no reaction
7	Using 10.0 equiv. of 3‐phenylpropanal	74
8	Using 2.0 equiv. of 3‐phenylpropanal	43
9	In air	69
10	In presence of H_2_O (10 equiv.)	42
11	Eosin Y (5 mol%) instead of TBADT	<10^[d]^
12	Anthraquinone (5 mol%)	<10 ^[d]^
13	Anthraquinone (5 mol%) with UV lamp (370 nm)	<10 ^[d]^

[a] Reactions were conducted using 0.30 mmol of **1 a** in MeCN (0.75 mL) under an argon atmosphere. [b] The blue LED source used has an emission spectrum with a small peak at ca. 390 nm (see Supporting Information for details). [c] Yield after isolation by column chromatography. [d] Only trace quantities of **2 aa** were detected in the crude reaction mixture, which was not purified.

The conditions of Table 1, entry 1 were selected to explore the reaction of enone **1 a** with other radical donors (Table 2). Other aliphatic aldehydes^[9]^ also reacted successfully to give **2 ab**–**2 ae**. In the case of 3‐(benzyloxy)propanal, the reaction was sluggish and **2 ac** was obtained in only 21 % yield. The reaction was much more efficient using cyclohexanecarboxaldehyde, which gave **2 ad** in 70 % yield. This experiment also led to the formation of a small quantity of **2 ak** (ca. 10 %, not isolated) resulting from decarbonylation of the initially formed acyl radical and subsequent 1,4‐addition.^[9a]^ Other acyl radical precursors such as 4‐substituted benzaldehydes^[9]^ (**2 af** and **2 ag**), and formamide^[14]^ (**2 ah**) also reacted successfully. Other radical precursors such as 1,3‐benzodioxole (**2 ai**)[^15^, ^16^] and cyclohexane (**2 ka**) can also be employed. With DMF, C−H functionalization occurred at one of the methyl groups to give **2 aj** in 31 % yield. For **2 ak**, 10 equiv. of cyclohexane were employed to achieve an acceptable yield. *tert*‐Butyl methyl ether also reacted successfully to give **2 al** in 34 % yield when UV irradiation was employed, as no conversion was obtained using blue LEDs. Aside from the products, the remaining mass balance in these reactions consisted mostly of unreacted starting materials. Although the yields of some of these reactions are modest, the ability to modify a complex, highly functionalized substrate such as **1 a** containing several potential competing sites of C−H functionalization (Figure 2) is notable. At present, there are relatively few such examples of TBADT‐catalyzed functionalizations of highly functionalized molecules.^[17]^

**Table 2:**
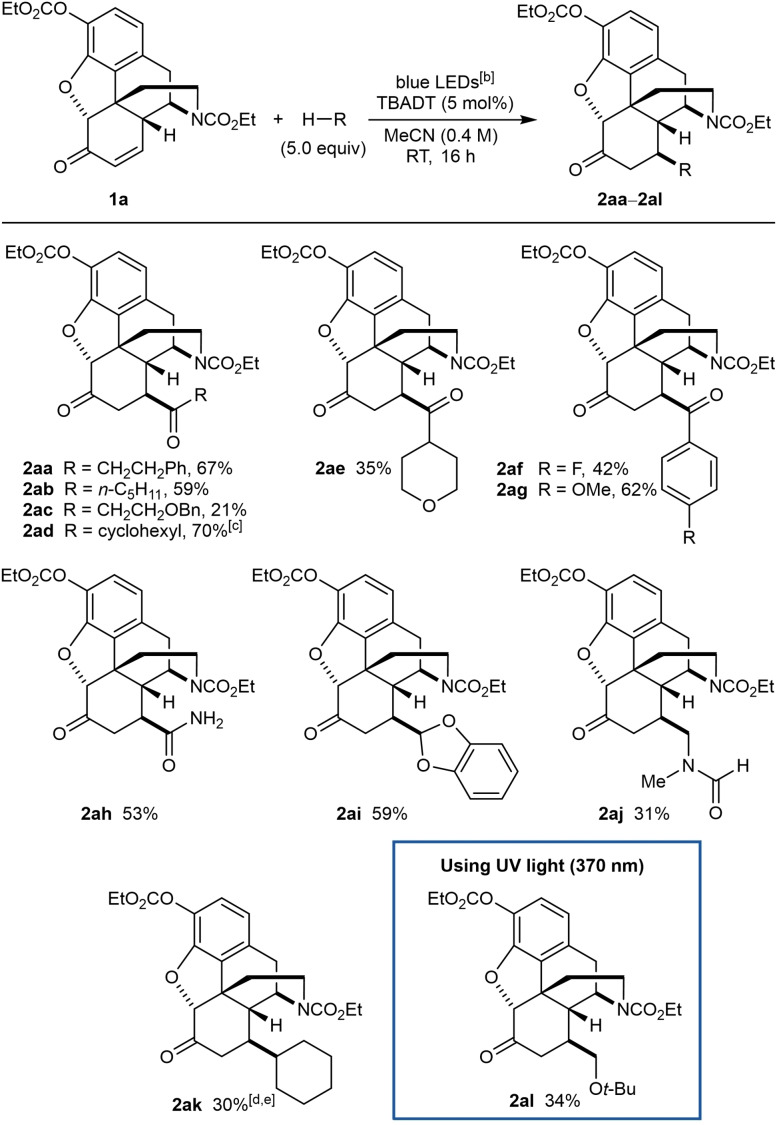
Scope of radical precursors.^[a]^

[a] Reactions were conducted using 0.30 mmol of **1 a**. Yields are of isolated products. [b] The blue LED source used has an emission spectrum with a small peak at ca. 390 nm (see Supporting Information for details). [c] Approximately 10 % of **2 ak** was also detected in the crude reaction mixture. [d] Using 10 equiv. of cyclohexane. [e] Conducted in MeCN (0.75 mL) and DCE (0.20 mL) to increase solubility.

We next investigated the use of other morphinan opioids containing an enone in the C‐ring and found that analogues of **1 a** containing alternative protecting groups on the phenol (TBS; **2 ba** or methyl; **2 ca** and **2 da**) and/or nitrogen atom (cyano; **2 da**) reacted successfully with 3‐phenylpropanal using blue LEDs to give products **2 ba**–**2 da** in 36–63 % yield (Table 3). In addition, under UV irradiation, codeinone (**1 e**) reacted with 3‐phenylpropanal (**2 ea**), 4‐methoxybenzaldehyde (**2 eg**), and 1,3‐benzodioxole (**2 ei**). Unfortunately, attempts to functionalize 14‐hydroxycodeinone (**1 f**) or enone **1 g** using blue LEDs or UV irradiation were not successful, and gave mostly recovered starting materials. In the case of **1 f**, poor solubility in MeCN may have been a contributing factor to the lack of reactivity.

**Table 3:**
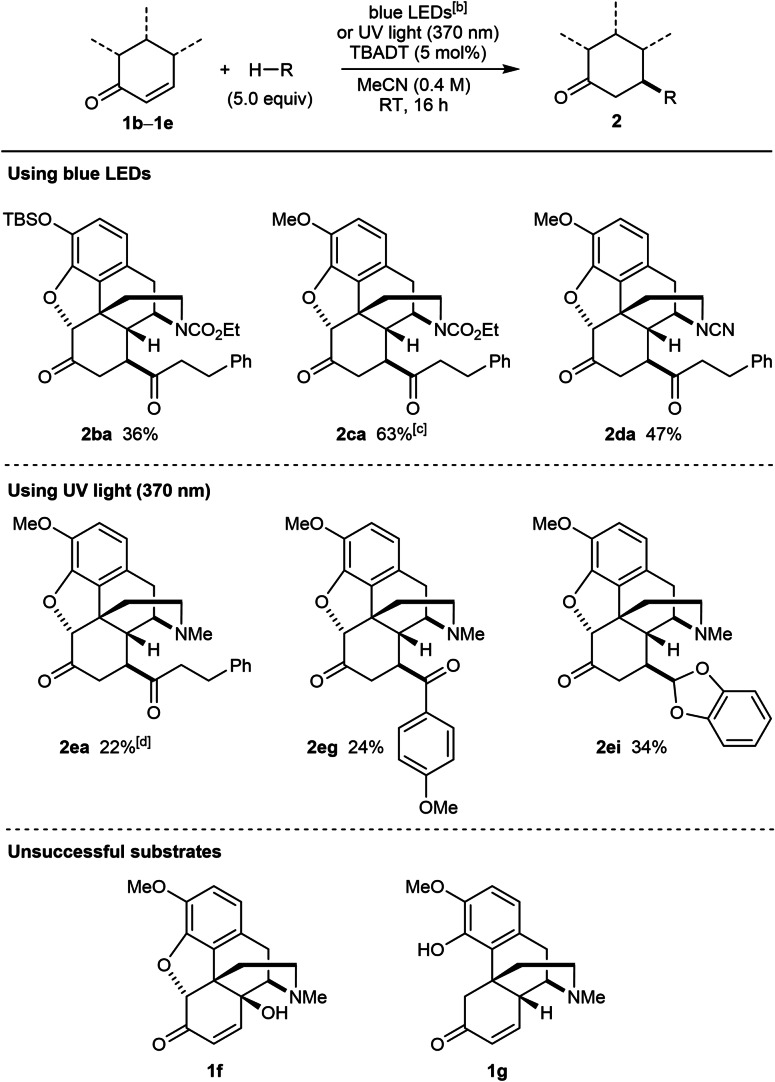
Scope of opioid radical acceptor.^[a]^

[a] Unless otherwise stated, reactions were conducted using 0.30 mmol of **1** in MeCN (3 mL). Yields are of isolated products. [b] The blue LED source used has an emission spectrum with a small peak at ca. 390 nm (see Supporting Information for details). [c] Conducted using 0.17 mmol of **1 c**. [d] Conducted using 0.18 mmol of **1 c**.

**Scheme 1:**
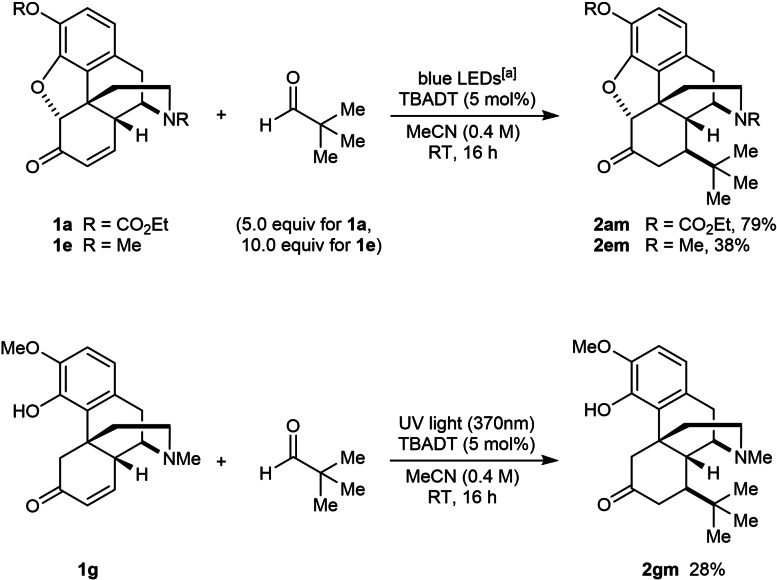
Decarbonylative radical additions using trimethylacetaldehyde. [a] The blue LED source used has an emission spectrum with a small peak at ca. 390 nm (see Supporting Information for details).

The use of trimethylacetaldehyde as the radical precursor led to decarbonylation of the initially formed acyl radical to give a *tert*‐butyl radical,^[9a]^ which added to enone **1 a** to give **2 am** in 79 % yield (Scheme 1). An analogous reaction with codeinone (**1 e**) gave **2 em** in 38 % yield. Interestingly, even though our previous attempts to functionalize enone **1 g** failed (Table 3), **1 g** reacted successfully with trimethylacetaldehyde under UV irradiation to give **2 gm**, albeit in a modest 28 % yield.

**Scheme 2:**
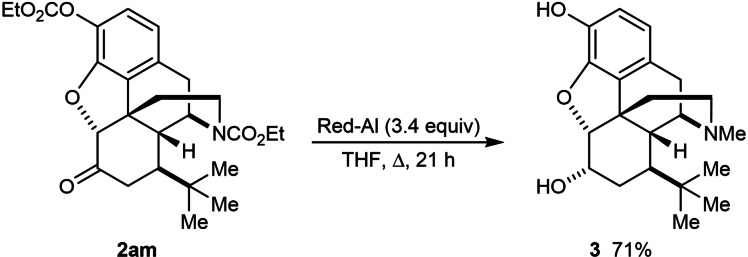
Removal of the ethoxycarbonyl groups in **2 am**.

Finally, to demonstrate the removal of the ethoxycarbonyl groups present in many of the products to give native functionality present in opiates, **2 am** was treated with sodium bis(2‐methoxyethoxy)aluminum hydride (Red‐Al) (Scheme 2). This experiment resulted in deprotection of the carbonate to the free phenol, conversion of the carbamate to an *N*‐methyl group, and stereoselective reduction of the ketone to give **3** in 71 % yield.

## Conclusion

In conclusion, we have reported the synthesis of new C8‐functionalized morphinan opioids by the TBADT‐catalyzed addition of photochemically generated carbon‐centered radicals to substrates containing an enone in the morphinan C‐ring. Despite the substrates containing several C−H bonds that could potentially react with TBADT, the products can be obtained in appreciable to good yields. This work demonstrates the power of late‐stage functionalization in the modification of complex substrates for the preparation of novel analogues.

## Conflict of interest

The authors declare no conflict of interest.

1

## Supporting information

As a service to our authors and readers, this journal provides supporting information supplied by the authors. Such materials are peer reviewed and may be re‐organized for online delivery, but are not copy‐edited or typeset. Technical support issues arising from supporting information (other than missing files) should be addressed to the authors.

Supporting Information

## Data Availability

The data that support the findings of this study are available in the supplementary material of this article and at: https://doi.org/10.17639/nott.7212.
