# Supplementary material for: Synthesis of New Morphinan Opioids by TBADT‐Catalyzed Photochemical Functionalization at the Carbon Skeleton
Source: Chemistry. 2022 Jul 13;28(50):e202201478. doi: 10.1002/chem.202201478 (PMC9544987; doi:10.1002/chem.202201478)
Supplement: Supplementary file 1 — Supporting Information [file CHEM-28-0-s001.pdf]

Chemistry–A European Journal

Supporting Information

Synthesis of New Morphinan Opioids by TBADT-Catalyzed Photochemical Functionalization at the Carbon Skeleton

Dmitry Gorbachev, Elliot Smith, Stephen P. Argent, Graham N. Newton, and Hon Wai Lam*

General Information 2
Preparation of Substrates for TBADT-catalyzed Radical Addition 4
General Procedure: TBADT-catalyzed Radical Additions to Enones 11
Product Derivatization 26
NMR Spectra of New Compounds 27
References 55

General Information

All commercially available reagents were used as received unless otherwise stated. TBADT was prepared according to literature procedure.[1] Petrol refers to Sigma-Aldrich product 24587 (petroleum ether boiling point 40−60 °C). Thin layer chromatography (TLC) was performed on Merck DF-Alufoilien 60F254 0.2 mm precoated plates. Compounds were visualized by exposure to UV light or by dipping the plates into solutions of potassium permanganate or vanillin followed by gentle heating. Flash column chromatography was carried out using silica gel (Fisher Scientific 60 Å particle size 35-70 micron or Fluorochem 60 Å particle size 40-63 micron). Melting points were recorded on a Gallenkamp melting point apparatus and are uncorrected. The solvent of recrystallization is reported in parentheses. Infrared (IR) spectra were recorded on Bruker platinum alpha FTIR spectrometer on the neat compound using the attenuated total reflectance technique. NMR spectra were acquired on Bruker Ascend 400 or Ascend 500 spectrometers. ^{1}H and ^{13}C NMR spectra were referenced to external tetramethylsilane via the residual protonated solvent (^{1}H) or the solvent itself (^{13}C). ^{19}F NMR spectra were referenced through the solvent lock (^{2}H) signal according to the IUPAC-recommended secondary referencing method following Bruker protocols. All chemical shifts are reported in parts per million (ppm). For $CDCl_3$, the shifts are referenced to 7.26 ppm for ^{1}H NMR spectroscopy and 77.16 ppm for ^{13}C NMR spectroscopy. For DMSO-d_6, the shifts are referenced to 2.50 ppm for ^{1}H NMR spectroscopy and 39.52 ppm for ^{13}C NMR spectroscopy. Coupling constants (*J*) are quoted to the nearest 0.1 Hz. For ^{13}C NMR spectroscopy, assignments were made using the DEPT sequence with secondary pulses at 90° and 135°. High resolution mass spectra were recorded on a Bruker MicrOTOF instrument using electrospray ionization (ESI) techniques. X-ray diffraction data were collected at 120 K on a XtalLAB PRO MM007 diffractometer using CuKα radiation. All photochemical reactions were performed in an EvoluChem™ PhotoRedOx Box reactor. Reactions under blue LED irradiation were performed using a 40W Kessil H160 Tuna Flora lamp set to BLUE at maximum intensity. The lamp emission spectrum is shown below (https://kessil.com/aquarium/horticulture_H160.php):

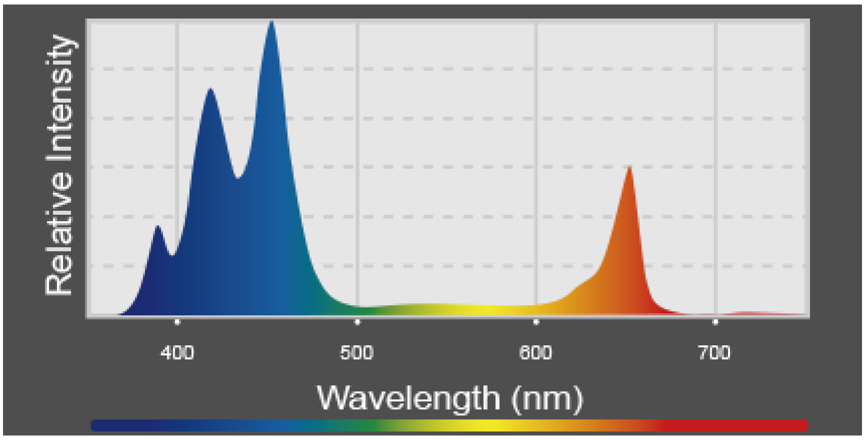

Reactions under UV irradiation were performed using a 40W Kessil PR160L-370 nm lamp set to maximum intensity. The lamp emission spectrum is shown below (https://kessil.com/science/PR160L-370.php):

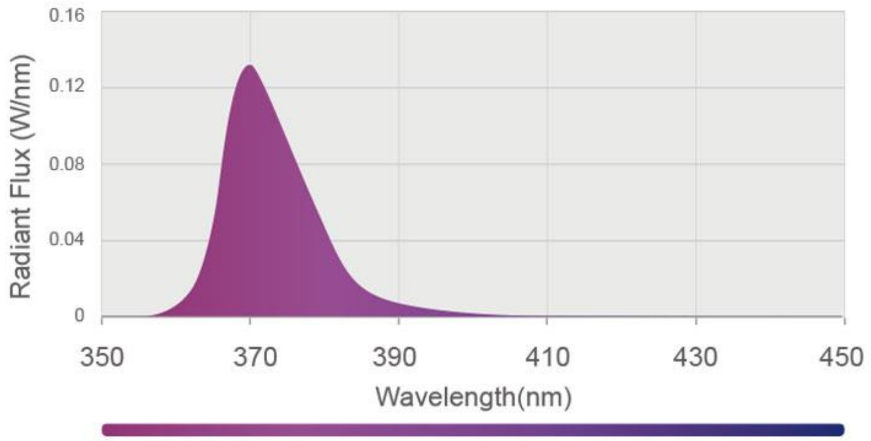

A representative reaction setup is shown below:

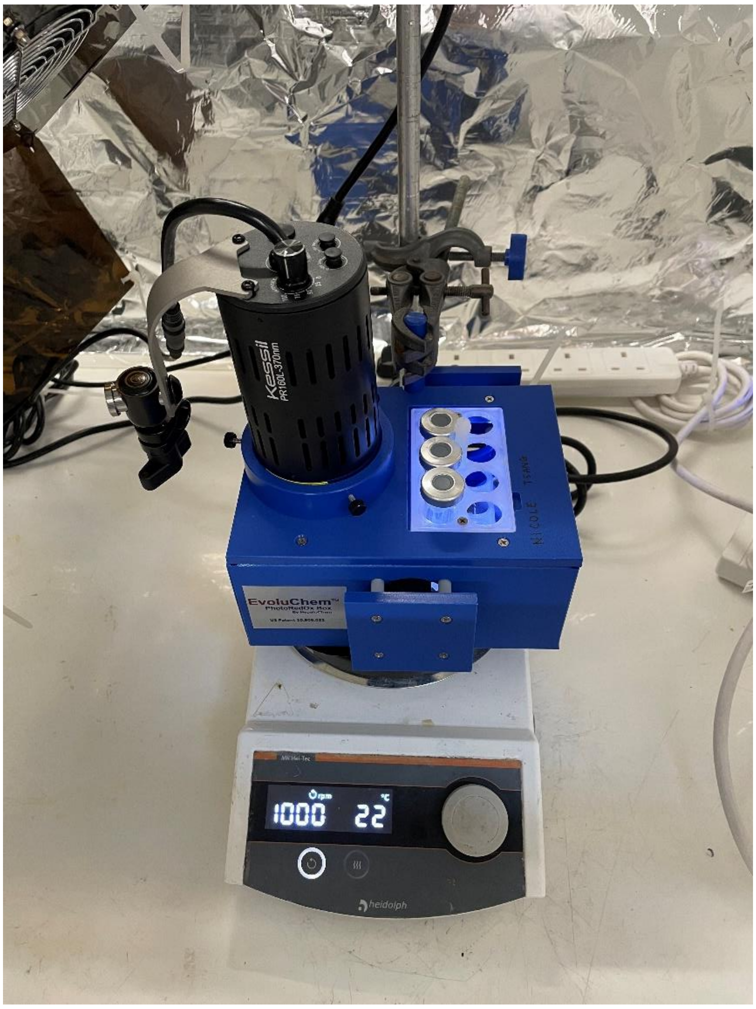

Preparation of Substrates for TBADT-Catalyzed Radical Addition

Ethyl (4*R*,4a*R*,7a*R*,12b*S*)-9-[(ethoxycarbonyl)oxy]-7-oxo-1,2,4,4a,7,7a-hexahydro-3*H*-4,12-methanobenzofuro[3,2-*e*]isoquinoline-3-carboxylate (1a)

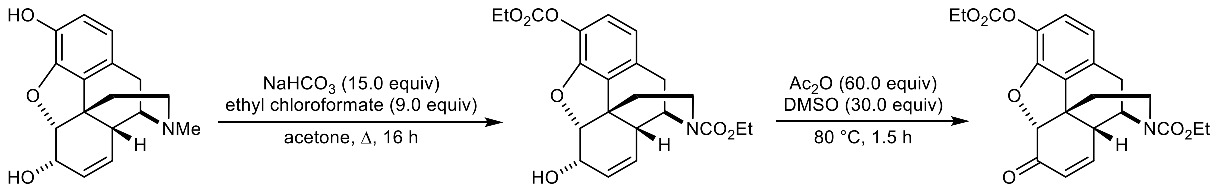

morphine (**S1**) **S2** 99% **1a** 80%

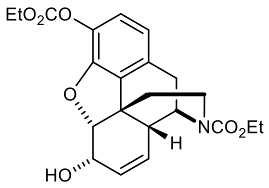

Ethyl (4*R*,4a*R*,7*S*,7a*R*,12b*S*)-9-[(ethoxycarbonyl)oxy]-7-hydroxy-1,2,4,4a,7,7a-hexahydro-3*H*-4,12-methanobenzofuro[3,2-*e*]isoquinoline-3-carboxylate (S2). To a stirred suspension of morphine (**S1**, 5.00 g, 17.5 mmol) and $NaHCO_3$ (22.1 g, 263 mmol) in acetone (50 mL) was added ethyl chloroformate (15.1 mL, 158 mmol) in one portion and the resulting mixture was heated under reflux for 16 h. The reaction was cooled to room temperature, filtered, washing through with acetone (50 mL), and concentrated *in vacuo* to give **S2** as a white solid (7.20 g, 99%), as a 1.3:1 mixture of rotamers that was used without further purification. R_f = 0.37 (50% EtOAc/petrol); m.p. 152-154 °C (Et_2O); $[\alpha]_D^{20.1}$ −244 (*c* 1.00, $CHCl_3$); IR (ATR) 3515 (OH), 2980, 2861, 1747 (C=O), 1688 (C=O), 1492, 1449, 1421, 1389, 1367 cm^{-1}; ^1H NMR (400 MHz, $CDCl_3$) δ 6.84 (1H, d, *J* = 8.2 Hz, Ar**H**), 6.60 (1H, d, *J* = 8.2 Hz, Ar**H**), 5.79 (1H, d, *J* = 8.9 Hz, HOCHC**H**=CH), 5.28 (1H, d, *J* = 8.9 Hz, HOCHCH=C**H**), 5.00-4.91 (1.57H, m, ArOC**H**, and C**H**N of rotamer A), 4.87-4.78 (0.43H, m, C**H**N, rotamer B), 4.39-4.24 (2H, m, $OCO_2C\mathbf{H}_2$), 4.24-4.01 (4H, m, $NCO_2C\mathbf{H}_2$ and $C\mathbf{H}_aH_bN$ and C**H**OH), 3.43 (1H, d, *J* = 11.5 Hz, O**H**), 3.03-2.93 (1H, m, $CH_a\mathbf{H}_bN$), 2.92-2.80 (1H, m, $ArC\mathbf{H}_aH_b$), 2.74 (1H, d, *J* = 18.8 Hz, $ArCH_a\mathbf{H}_b$), 2.60-2.53 (1H, m, C**H**CHN), 1.99-1.90 (2H, m, $C\mathbf{H}_2CH_2N$), 1.37 (3H, t, *J* = 7.2 Hz, $OCO_2CH_2C\mathbf{H}_3$), 1.34-1.23 (3H, m, $NCO_2CH_2C\mathbf{H}_3$); **Rotamer A (Major)** ^{13}C NMR (101 MHz, $CDCl_3$) δ 155.5 (C), 153.1 (C), 148.8 (C), 135.1 (CH), 132.8 (C), 132.0 (C), 131.7 (C), 126.6 (CH), 121.6 (CH), 120.4 (CH), 92.4 (CH), 65.7 (CH), 65.6 (CH_2), 61.7 (CH_2), 50.2 (CH), 43.2 (C), 39.4 (CH), 37.5 (CH_2), 34.9 (CH_2), 29.8 (CH_2), 14.8 (CH_3), 14.3 (CH_3); **Rotamer B (Minor)** ^{13}C NMR (101 MHz, $CDCl_3$) δ 155.2 (C), 153.1 (C), 148.8 (C), 135.4 (CH), 132.8 (C), 131.8 (C), 131.7 (C), 126.5 (CH), 121.6 (CH), 120.4 (CH), 92.4 (CH), 65.7 (CH), 65.6 (CH_2), 61.7 (CH_2), 50.5 (CH), 43.2 (C), 39.5 (CH), 37.4 (CH_2), 34.6 (CH_2), 30.0 (CH_2), 14.8 (CH_3), 14.7 (CH_3); HRMS (ESI) Exact mass calculated for $[C_{22}H_{26}NO_7]^+$ $[M+H]^+$: 416.1704, found 416.1702.

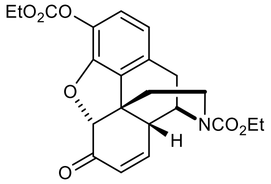

Ethyl (4*R*,4a*R*,7a*R*,12b*S*)-9-[(ethoxycarbonyl)oxy]-7-oxo-1,2,4,4a,7,7a-hexahydro-3*H*-4,12-methanobenzofuro[3,2-*e*]isoquinoline-3-carboxylate (1a). To a stirred solution of **S2** (2.25 g, 5.41 mmol) in Ac_2O (30.7 mL, 325 mmol) at room temperature was added DMSO (11.5 mL, 162 mmol) and the resulting mixture was stirred at 70 °C for 1.5 h. The reaction was cooled to room temperature, diluted with H_2O (100 mL), and extracted with Et_2O (3 × 50 mL). The combined organic extracts were washed with saturated aqueous $NaHCO_3$ solution (3 × 100 mL), brine (100 mL), dried ($MgSO_4$), and concentrated *in vacuo*. The crude product was recrystallized from EtOAc to give **1a** as a colorless solid (1.79 g, 80%), as a 1.9:1 mixture of rotamers. R_f = 0.20 (50% EtOAc/petrol); m.p. 176-177 °C (Et_2O); $[\alpha]_D^{20.1}$ −160 (*c* 1.00, $CHCl_3$); IR (ATR) 2987, 1768 (C=O), 1675 (C=O), 1624 (C=O), 1494, 1420, 1377, 1318, 1232, 1216 cm^{-1}; ^{1}H NMR (400 MHz, $CDCl_3$) δ 6.91 (1H, d, *J* = 8.2 Hz, Ar**H**), 6.70-6.61 (2H, m, Ar**H** and O=CC**H**=CH), 6.13 (1H, dd, *J* = 10.2, 2.9 Hz, O=CCH=C**H**), 5.11-5.01 (0.65H, m, C**H**N, rotamer A), 4.95-4.87 (0.35H, m, C**H**N, rotamer B), 4.76 (1H, s, ArOC**H**), 4.33-4.24 (2H, m, $OCO_2C\mathbf{H}_2$), 4.23-3.98 (3H, m, $NCO_2C\mathbf{H}_2$ and C$\mathbf{H}_a$$H_b$), 3.09-3.02 (1H, m, C**H**CHN), 2.96-2.76 (3H, m, $CH_a\mathbf{H}_b$ and ArC$\mathbf{H}_2$), 2.05-1.87 (2H, m, C$\mathbf{H}_2$$CH_2$N), 1.35 (3H, t, *J* = 7.1 Hz, $OCO_2CH_2C\mathbf{H}_3$), 1.34-1.23 (3H, m, $NCO_2CH_2C\mathbf{H}_3$); **Rotamer A (Major)** ^{13}C NMR (101 MHz, $CDCl_3$) δ 193.0 (C), 155.7 (C), 152.8 (C), 147.9 (C), 147.2 (CH), 133.4 (CH), 133.1 (C), 130.7 (C), 129.0 (C), 122.9 (CH), 120.6 (CH), 88.5 (CH), 65.3 (CH_2), 62.0 (CH_2), 50.2 (CH), 43.7 (C), 40.3 (CH), 38.0 (CH_2), 33.6 (CH_2), 29.5 (CH_2), 14.7 (CH_3), 14.2 (CH_3); **Rotamer B (Minor)** ^{13}C NMR (101 MHz, $CDCl_3$) δ 193.0 (C), 155.7 (C), 152.8 (C), 147.9 (C), 146.9 (CH), 133.4 (CH), 133.1 (C), 130.5 (C), 127.0 (C), 122.9 (CH), 120.6 (CH), 88.5 (CH), 65.3 (CH_2), 62.0 (CH_2), 50.6 (CH), 43.7 (C), 40.3 (CH), 38.0 (CH_2), 33.4 (CH_2), 29.8 (CH_2), 14.7 (CH_3), 14.2 (CH_3); HRMS (ESI) Exact mass calculated for $[C_{22}H_{24}NO_7]^+$ $[M+H]^+$: 414.1547, found 414.1549.

Ethyl (4*R*,4a*R*,7a*R*,12b*S*)-9-[(*tert*-butyldimethylsilyl)oxy]-7-oxo-1,2,4,4a,7,7a-hexahydro-3*H*-4,12-methanobenzofuro[3,2-*e*]isoquinoline-3-carboxylate (1b)

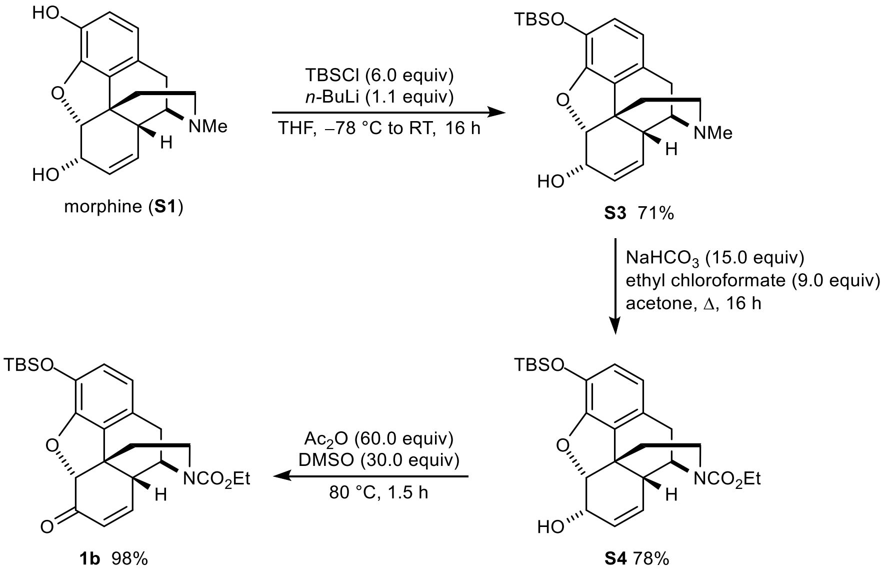

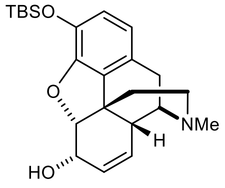

(4*R*,4a*R*,7*S*,7a*R*,12b*S*)-9-[(*tert*-Butyldimethylsilyl)oxy]-3-methyl-2,3,4,4a,7,7a-hexahydro-1*H*-4,12-methanobenzofuro[3,2-*e*]isoquinolin-7-ol (S3).[2] To a stirred suspension of morphine (**S1**, 1.00 g, 3.50 mmol) in THF (12 mL) at −78 °C was added *n*-BuLi (2.44 M in hexanes, 1.58 mL, 3.85 mmol) and the mixture was stirred for 30 min. A solution of TBSCl (3.17 g, 21.0 mmol) in THF (5 mL) was then added and the resulting mixture was stirred for 16 h while allowing it to warm to toom temperature. The reaction was quenched with H_2O (50 mL) and extracted with CH_2Cl_2 (3 × 50 mL). The combined organic extracts were washed with brine (150 mL), dried ($MgSO_4$), and concentrated *in vacuo*. Purification of the residue by column chromatography (5% 2 M NH_3[MeOH]/CH_2Cl_2) gave **S3** as a white solid (988 mg, 71%). The analytical data were consistent with those reported previously.[2]

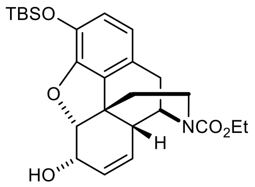

Ethyl (4*R*,4a*R*,7*S*,7a*R*,12b*S*)-9-[(*tert*-butyldimethylsilyl)oxy]-7-hydroxy-1,2,4,4a,7,7a-hexahydro-3*H*-4,12-methanobenzofuro[3,2-*e*]isoquinoline-3-carboxylate (S4). To a stirred suspension of **S3** (875 mg, 2.19 mmol) and $NaHCO_3$ (2.76 g, 32.9 mmol) in acetone (12 mL) was added ethyl chloroformate (1.9 mL, 19.7 mmol) in one portion and the resulting mixture was heated under reflux for 16 h. The reaction was cooled to room temperature, filtered, washing through with acetone (12 mL), and concentrated *in vacuo*. Purification of the residue by column chromatography (50% EtOAc/petrol) gave **S4** as a white solid (780 mg, 78%) as a 1.5:1 mixture of rotamers. R_f = 0.26 (50% EtOAc/petrol); m.p. 127-130 °C (Et_2O); $[\alpha]_D^{20.1}$ −120 (*c* 1.00, $CHCl_3$); IR (ATR) 3421 (OH), 2929, 2856, 1658 (C=O), 1496, 1472, 1447, 1425, 1388, 1372 cm^{-1}; ^1H NMR (400 MHz, $CDCl_3$) δ 6.59

(1H, d, J = 8.1 Hz, Ar**H**), 6.48 (1H, d, J = 8.1 Hz, Ar**H**), 5.71 (1H, dd, J = 9.9, 4.2 Hz, O=CCH=C**H**), 5.32-5.23 (1H, m, O=CC**H**=CH), 4.97-4.87 (0.6H, m, C**H**N, rotamer A), 4.86-4.76 (1.4H, m, C**H**N, rotamer B and ArOC**H**), 4.26-3.93 (4H, m, C**H**OH and C$\mathbf{H}_2$CH$_3$ and C$\mathbf{H}_a$H$_b$N), 3.09-2.92 (1H, m, CH$_a$$\mathbf{H}_b$N), 2.91-2.78 (1H, m, ArC$\mathbf{H}_a$H$_b$), 2.70 (1H, d, J = 18.6 Hz, ArCH$_a$$\mathbf{H}_b$), 2.53-2.47 (1H, m, C**H**CHN), 1.99-1.81 (2H, m, C$\mathbf{H}_2$CH$_2$N), 1.34-1.22 (3H, m, CH$_2$C$\mathbf{H}_3$), 0.98 (9H, s, C(C$\mathbf{H}_3$)$_3$), 0.19 (3H, s, Si(C$\mathbf{H}_3$)$_a$), 0.15 (3H, s, Si(C$\mathbf{H}_3$)$_b$); **Rotamer A (Major)** ^{13}C NMR (101 MHz, CDCl$_3$) δ 155.5 (C), 148.5 (C), 137.7 (C), 134.1 (CH), 130.3 (C), 127.3 (CH), 126.6 (C), 121.6 (CH), 120.2 (CH), 90.8 (CH), 66.2 (CH), 61.6 (CH$_2$), 50.2 (CH), 43.5 (C), 39.7 (CH), 37.5 (CH$_2$), 35.6 (CH$_2$), 29.6 (CH$_2$), 25.7 (3 × CH$_3$), 18.4 (C), 14.8 (CH$_3$), −4.3 (CH$_3$), −4.5 (CH$_3$); **Rotamer B (Minor)** ^{13}C NMR (101 MHz, CDCl$_3$) δ 155.2 (C), 148.5 (C), 137.7 (C), 134.4 (CH), 130.3 (C), 127.1 (CH), 126.4 (C), 121.6 (CH), 120.2 (CH), 90.8 (CH), 66.2 (CH), 61.6 (CH$_2$), 50.5 (CH), 43.5 (C), 39.9 (CH), 37.4 (CH$_2$), 35.3 (CH$_2$), 29.8 (CH$_2$), 25.7 (3 × CH$_3$), 18.4 (C), 14.8 (CH$_3$), −4.3 (CH$_3$), −4.5 (CH$_3$); HRMS (ESI) Exact mass calculated for [C$_{25}$H$_{36}$NO$_5$Si]$^+$ [M+H]$^+$: 458.2357, found 458.2357.

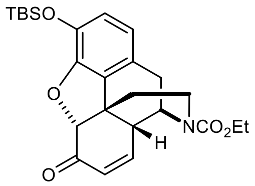

Ethyl (4*R*,4a*R*,7a*R*,12b*S*)-9-[(*tert*-butyldimethylsilyl)oxy]-7-oxo-1,2,4,4a,7,7a-hexahydro-3*H*-4,12-methanobenzofuro[3,2-*e*]isoquinoline-3-carboxylate (1b). To a stirred solution of **S4** (91 mg, 0.20 mmol) in Ac$_2$O (0.60 mL, 6.00 mmol) was added DMSO (0.20 mL, 3.00 mmol) and the resulting mixture was stirred at 70 °C for 1.5 h. The reaction was cooled to room temperature, diluted with H$_2$O (5 mL), and extracted with Et$_2$O (3 × 5 mL). The combined organic extracts were washed with saturated aqueous NaHCO$_3$ solution (3 × 20 mL), brine (20 mL), dried (MgSO$_4$), and concentrated *in vacuo* to give **1b** as a white solid (89 mg, 98%), as a 1.5:1 mixture of rotamers. R$_f$ = 0.24 (50% EtOAc/petrol); m.p. 111-116 °C (Et$_2$O); $[\alpha]_D^{20.1}$ −130 (*c* 1.00, CHCl$_3$); IR (ATR) 2929, 2857, 1674 (C=O), 1659 (C=O), 1496, 1470, 1417, 1317, 1267, 1230 cm^{-1}; ^{1}H NMR (400 MHz, CDCl$_3$) δ 6.67-6.59 (2H, m, Ar**H** and O=CC**H**=CH), 6.54 (1H, d, J = 8.2 Hz, Ar**H**), 6.11 (1H, dd, J = 10.2, 2.8 Hz, O=CCH=C**H**), 5.05-5.00 (0.6H, m, C**H**N, rotamer A), 4.90-4.84 (0.4H, m, C**H**N, rotamer B), 4.65 (1H, s, ArOC**H**), 4.27-3.95 (3H, m, C$\mathbf{H}_2$CH$_3$ and C$\mathbf{H}_a$H$_b$N), 3.05-2.98 (1H, m, C**H**CHN), 2.95-2.83 (1H, m, CH$_a$$\mathbf{H}_b$N), 2.83-2.78 (2H, m, ArC$\mathbf{H}_2$), 2.00-1.82 (2H, m, C$\mathbf{H}_2$CH$_2$N), 1.36-1.22 (3H, m, CH$_2$C$\mathbf{H}_3$), 0.96 (9H, s, C(C$\mathbf{H}_3$)$_3$), 0.18 (3H, s, Si(C$\mathbf{H}_3$)$_a$), 0.12 (3H, s, Si(C$\mathbf{H}_3$)$_b$); **Rotamer A (Major)** ^{13}C NMR (101 MHz, CDCl$_3$) δ 193.9 (C), 155.7 (C), 147.4 (C), 147.1 (CH), 138.1 (C), 133.3 (CH), 128.1 (C), 125.4 (C), 122.9 (CH), 120.5 (CH), 87.7 (CH), 61.9 (CH$_2$), 50.4 (CH), 43.7 (C), 40.5 (CH), 38.1 (CH$_2$), 33.8 (CH$_2$), 29.4 (CH$_2$), 25.8 (3 × CH$_3$), 18.4 (C), 14.8 (CH$_3$), −4.5 (CH$_3$), −4.6 (CH$_3$); **Rotamer B (Minor)** ^{13}C NMR (101 MHz, CDCl$_3$) δ 193.9 (C), 155.7 (C), 147.4 (C), 147.1 (CH), 138.1 (C), 133.3 (CH), 128.1 (C), 125.4 (C), 122.9 (CH), 120.5 (CH), 87.7 (CH), 61.9 (CH$_2$), 50.8 (CH), 43.7 (C), 40.5 (CH), 38.1 (CH$_2$), 33.5 (CH$_2$), 29.4 (CH$_2$), 25.8 (3 × CH$_3$), 18.4 (C), 14.8 (CH$_3$),

-4.5 (CH_3), −4.6 (CH_3); HRMS (ESI) Exact mass calculated for $[C_{25}H_{34}NO_5Si]^+$ $[M+H]^+$: 456.2201, found 456.2202.

Ethyl (4*R*,4a*R*,7a*R*,12b*S*)-9-methoxy-7-oxo-1,2,4,4a,7,7a-hexahydro-3*H*-4,12-methanobenzofuro[3,2-*e*]isoquinoline-3-carboxylate (1c)

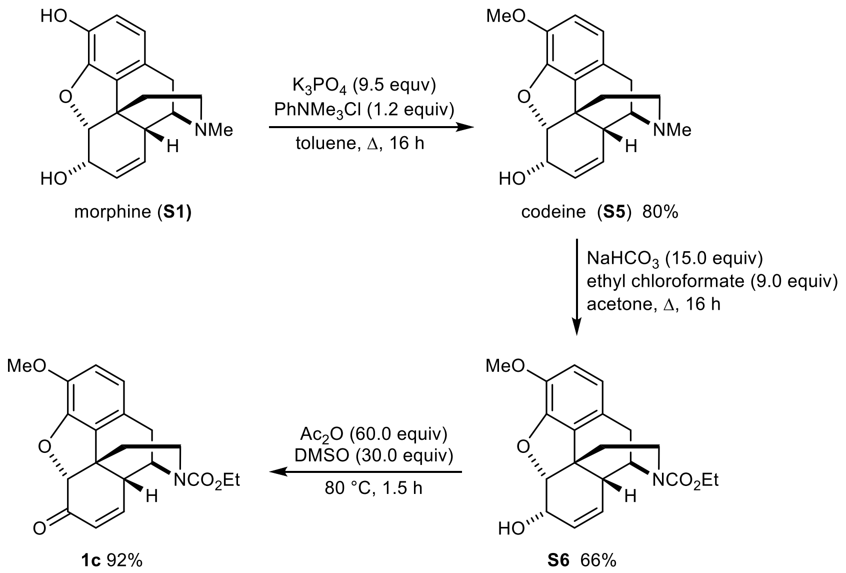

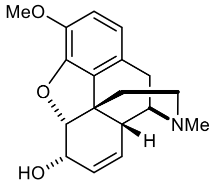

Codeine: (4*R*,4a*R*,7*S*,7a*R*,12b*S*)-9-methoxy-3-methyl-2,3,4,4a,7,7a-hexahydro-1*H*-4,12-methanobenzofuro[3,2-*e*]isoquinolin-7-ol (S5).[3] A mixture of morphine **S1** (2.50 g, 8.75 mmol), K_3PO_4 (17.6 g, 83.1 mmol), trimethylphenylammonium chloride (1.80 g, 10.50 mmol), and toluene (125 mL), was stirred under reflux for 16 h. The reaction was cooled to room temperature, filtered through a short plug of celite, washing through with toluene (50 mL), and concentrated *in vacuo*. Purification of the residue by column chromatography (10% 2 M NH_3[MeOH]/CH_2Cl_2) gave codeine (**S5)** as an off-white foam (2.10 g, 80%). The analytical data were consistent with those reported previously.[3]

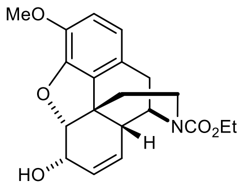

Ethyl (4*R*,4a*R*,7*S*,7a*R*,12b*S*)-7-hydroxy-9-methoxy-1,2,4,4a,7,7a-hexahydro-3*H*-4,12-methanobenzofuro[3,2-*e*]isoquinoline-3-carboxylate (S6).[4] To a stirred suspension of codeine **S5** (641 mg, 2.14 mmol) and $NaHCO_3$ (2.70 g, 32.1 mmol) in acetone (12 mL) was added ethyl chloroformate (1.8 mL, 19.3 mmol) in one portion and the resulting mixture was heated under reflux for 16 h. The reaction was cooled to room temperature, filtered, washing through with acetone (12 mL.) and concentrated *in vacuo*. Purification of the residue by column chromatography (40% EtOAc/petrol) gave **S6** as a white foam (502 mg, 66%). The analytical data were consistent with those reported previously.[4]

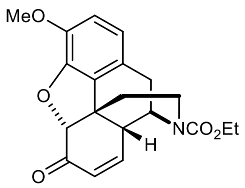

Ethyl (4*R*,4a*R*,7a*R*,12b*S*)-9-methoxy-7-oxo-1,2,4,4a,7,7a-hexahydro-3*H*-4,12-methanobenzofuro[3,2-*e*]isoquinoline-3-carboxylate (1c).[4] To a stirred solution of **S6** (72 mg, 0.20 mmol) in Ac_2O (0.60 mL, 6.00 mmol) was added DMSO (0.20 mL, 3.00 mmol) and the resulting mixture was stirred at 70 °C for 1.5 h. The reaction was cooled to room temperature, diluted with H_2O (5 mL), and extracted with Et_2O (3 × 5 mL). The combined organic extracts were washed with saturated aqueous $NaHCO_3$ solution (3 × 20 mL), brine (20 mL), dried ($MgSO_4$), and concentrated *in vacuo* to give **1c** as a white foam (65 mg, 92%). The analytical data were consistent with those reported previously.[4]

(4*R*,4a*R*,7a*R*,12b*S*)-9-Methoxy-7-oxo-1,2,4,4a,7,7a-hexahydro-3*H*-4,12-methanobenzofuro[3,2-*e*]isoquinoline-3-carbonitrile (1d)

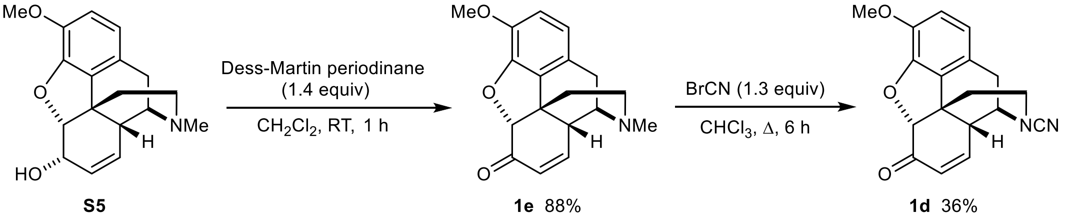

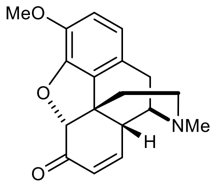

(4*R*,4a*R*,7a*R*,12b*S*)-9-Methoxy-3-methyl-2,3,4,4a-tetrahydro-1*H*-4,12-methanobenzofuro[3,2-*e*]isoquinolin-7(7a*H*)-one (1e).[2] To a stirred solution of **S5** (1.4 g, 4.72 mmol) in CH_2Cl_2 (60 mL) was added Dess-Martin Periodinane (2.80 g, 6.61 mmol) in one portion and the resulting mixture was stirred at room temperature for 1 h. 1 M Aqueous NaOH solution (60 mL) was then added and the mixture was stirred for a further 1 h. The layers were separated, and aqueous layer was extracted with CH_2Cl_2 (60 mL). The combined organic layers were washed with brine (100 mL), dried (Na_2SO_4), and concentrated *in vacuo* to give **1e** as an off-white foam (1.12 g, 80%), which was used in the subsequent step without further purification. The analytical data were consistent with those reported previously.[2]

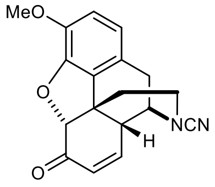

(4*R*,4a*R*,7a*R*,12b*S*)-9-Methoxy-7-oxo-1,2,4,4a,7,7a-hexahydro-3*H*-4,12-methanobenzofuro[3,2-*e*]isoquinoline-3-carbonitrile (1d). A round-bottom flask was charged with **1e** (446 mg, 1.50 mmol), BrCN (207 mg, 1.95 mmol), and $CHCl_3$ (9 mL), and the resulting mixture was stirred under reflux for 6 h. The reaction was cooled to room temperature, diluted with $CHCl_3$ (50 mL), and washed with 1 M aqueous HCl solution (3 × 50 mL), 2 M aqueous NaOH solution (50 mL), H_2O (50 mL), brine (50 mL). The organic solution was dried (Na_2SO_4) and concentrated *in vacuo*. Purification of the residue by column chromatography (60% EtOAc/petrol) gave **1d** as an off-white solid (166 mg, 36%). R_f = 0.25 (60%

EtOAc/petrol); m.p. 155-159 °C (Et_2O); $[\alpha]_D^{20.1}$ –212 (*c* 1.00, $CHCl_3$); IR (ATR) 2935, 2836, 2206 (C≡N), 1670 (C=O), 1503, 1438, 1400, 1320, 1304, 1275 cm^{-1}; ^1H NMR (400 MHz, $CDCl_3$) δ 6.73 (1H, d, *J* = 8.2 Hz, Ar**H**), 6.67 (1H, d, *J* = 8.2 Hz, Ar**H**), 6.56 (1H, dd, *J* = 10.3, 2.1 Hz, O=CC**H**=CH), 6.14 (1H, dd, *J* = 10.3, 2.9 Hz, O=CCH=C**H**), 4.70 (1H, s, ArOC**H**), 4.11 (1H, ddd, *J* = 5.3, 3.4, 1.4 Hz, C**H**N), 3.84 (3H, s, OC$\mathbf{H}_3$), 3.35-3.28 (2H, m, C**H**CHCN and C$\mathbf{H}_a$$H_b$N), 3.22-3.12 (2H, m, C$H_a$$\mathbf{H}_b$N and ArC$\mathbf{H}_a$$H_b$), 2.85 (1H, dd, *J* = 18.5, 4.3 Hz, ArC$H_a$$\mathbf{H}_b$), 2.15 (1H, td, *J* = 12.7, 5.5 Hz, C$\mathbf{H}_a$$H_b$$CH_2$N), 1.94 (1H, ddd, *J* = 13.0, 4.0, 1.5 Hz, C$H_a$$\mathbf{H}_b$$CH_2$N); ^{13}C NMR (101 MHz, $CDCl_3$) δ 193.4 (C), 145.4 (CH), 145.1 (C), 143.4 (C), 133.7 (CH), 127.2 (C), 123.6 (C), 120.8 (CH), 117.4 (C), 115.6 (CH), 87.5 (CH), 57.2 (CH), 56.9 (CH_3), 43.9 (CH_2), 42.8 (C), 39.8 (CH), 32.7 (CH_2), 28.6 (CH_2); HRMS (ESI) Exact mass calculated for $[C_{18}H_{17}N_2O_3]^+$ $[M+H]^+$: 309.1234, found 309.1232.

(4b*S*,8a*R*,9*R*)-4-Hydroxy-3-methoxy-11-methyl-9,10-dihydro-5*H*-9,4b-(epiminoethano)phenanthren-6(8a*H*)-one (1g)[5]

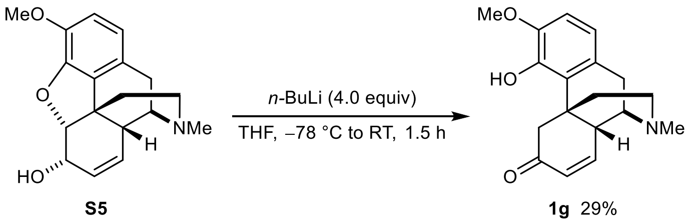

To a stirred solution of **S5** (1.70 g, 5.68 mmol) in THF (71 mL) at −78 °C was added *n*-BuLi (2.38 M in hexanes, 9.56 mL, 22.7 mmol) and the resulting mixture was stirred at −78 °C for 1 h. The reaction was warmed to room temperature and stirred for a further 30 min. The reaction was quenched with H_2O (30 mL) and extracted with CH_2Cl_2 (3 × 15 mL). The combined organic extracts were washed with brine (30 mL), dried (Na_2SO_4), and concentrated *in vacuo*. Purification of the residue by column chromatography (10% 2 M NH_3[MeOH]/CH_2Cl_2) gave **1g** as a beige foam (494 mg, 29%). The analytical data were consistent with those reported previously.[5]

General Procedure: TBADT-Catalyzed Radical Additions to Enones

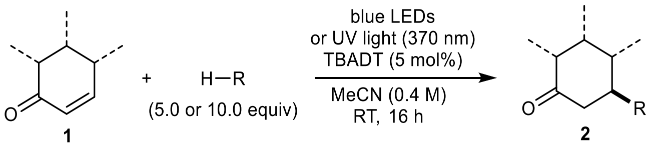

An oven-dried microwave vial fitted with a magnetic stirrer bar was charged with the appropriate enone (0.30 mmol) and TBADT (50.0 mg, 0.015 mmol). The vial was capped with a crimp cap seal and flushed with argon (5 min). Freshly degassed MeCN (0.75 mL) and the appropriate radical donor (1.50 mmol or 3.00 mmol) were added, the cap was sealed with PTFE tape, and the contents were stirred at room temperature for 16 h under blue LED irradiation using a A160WE Kessil Tuna Blue lamp or UV irradiation using a 40W Kessil PR160L-370 nm lamp. The reaction mixture was concentrated *in vacuo* and purified by column chromatography to give the addition product **2**.

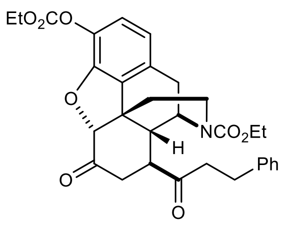

Ethyl (4*R*,4a*R*,5*S*,7a*R*,12b*S*)-9-[(ethoxycarbonyl)oxy]-7-oxo-5-(3-phenylpropanoyl)-1,2,4,4a,5,6,7,7a-octahydro-3*H*-4,12-methanobenzofuro[3,2-*e*]isoquinoline-3-carboxylate (2aa). Prepared according to the General Procedure using enone **1a** (124 mg, 0.30 mmol) and 3-phenylpropanal (0.20 mL, 1.50 mmol) as the radical donor, under blue LED irradiation. Purification by column chromatography (50% EtOAc/petrol) gave **2aa** (110 mg, 67%) as a white foam, as a *ca.* 1:1 mixture of rotamers. R_f = 0.33 (50% EtOAc/petrol); m.p. 133-138 °C (Et_2O); $[\alpha]_D^{20.1}$ −180 (*c* 1.00, $CHCl_3$); IR (ATR) 2929, 2109, 1760 (C=O), 1732 (C=O), 1687 (C=O), 1624 (C=O), 1495, 1443, 1370, 1316 cm^{-1}; ^1H NMR (400 MHz, $CDCl_3$) δ 7.34-7.13 (5H, m, $C_6\mathbf{H}_5$), 6.99 (1H, d, *J* = 8.2 Hz, Ar**H**), 6.71 (1H, d, *J* = 8.2 Hz, Ar**H**), 4.77-4.66 (1.5H, m, ArOC**H** and C**H**N, rotamer A), 4.58-4.53 (0.5H, m, C**H**N, rotamer B), 4.39-4.31 (2H, m, $OCO_2C\mathbf{H}_2$), 4.26-3.94 (3H, m, $NCO_2C\mathbf{H}_2$ and $C\mathbf{H}_aH_bN$), 3.02-2.88 (2H, m, $C\mathbf{H}_2CH_2Ph$), 2.88-2.55 (6H, m, $CH_2C\mathbf{H}_2Ph$ and $ArC\mathbf{H}_2$ and $CH_a\mathbf{H}_bN$ and O=$CCH_2C\mathbf{H}$), 2.45-2.26 (3H, m, O=$CC\mathbf{H}_2CH$ and C**H**CHN), 2.09-1.97 (1H, m, $C\mathbf{H}_aH_bCH_2N$), 1.96-1.85 (1H, m, $CH_a\mathbf{H}_bCH_2N$), 1.40 (3H, t, *J* = 7.1 Hz, $OCO_2CH_2C\mathbf{H}_3$), 1.37-1.25 (3H, m, $NCO_2CH_2C\mathbf{H}_3$); **Rotamer A** ^{13}C NMR (101 MHz, $CDCl_3$) δ 207.6 (C), 203.9 (C), 176.1 (C), 155.1 (C), 152.9 (C), 147.9 (C), 140.4 (C), 133.5 (C), 130.4 (C), 128.8 (2 × CH), 128.4 (CH), 126.5 (2 × CH), 123.3 (CH), 120.6 (CH), 91.4 (CH), 65.3 (CH_2), 61.9 (CH_2), 48.4 (CH), 48.0 (CH), 46.9 (C), 44.8 (CH_2), 42.6 (CH), 42.0 (CH_2), 37.8 (CH_2), 35.0 (CH_2), 29.5 (CH_2), 28.8 (CH_2), 14.8 (CH_3), 14.3 (CH_3); **Rotamer B** ^{13}C NMR (101 MHz, $CDCl_3$) δ 207.9 (C), 204.1 (C), 176.1 (C), 155.5 (C), 152.9 (C), 147.9 (C), 140.4 (C), 133.5 (C), 130.7 (C), 128.8 (2 × CH), 128.4 (CH), 126.5 (2 × CH), 123.3 (CH), 120.6 (CH), 91.4 (CH), 65.3 (CH_2), 61.9 (CH_2), 48.5 (CH), 48.2 (CH), 46.9 (C), 44.8 (CH_2), 42.6 (CH), 42.0 (CH_2), 38.0 (CH_2), 35.0 (CH_2), 29.7 (CH_2), 28.8 (CH_2), 14.8 (CH_3),

14.3 (CH_3); HRMS (ESI) Exact mass calculated for $[C_{31}H_{34}NO_8]^+$ $[M+H]^+$: 548.2279, found 548.2270.

Vapor diffusion of Et_2O into a solution of **2aa** in *t*-BuOH gave crystals that were suitable for X-ray crystallography:

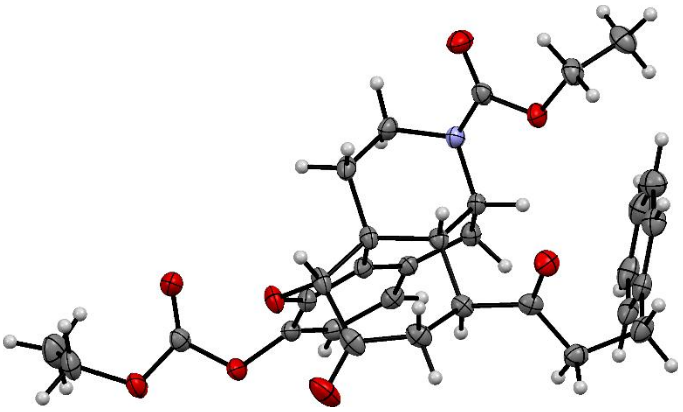

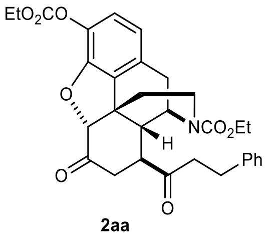

2aa
CCDC 2149928

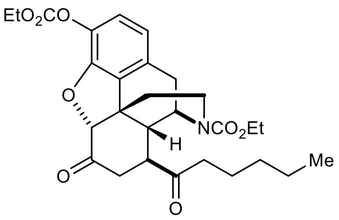

Ethyl (4*R*,4a*R*,5*S*,7a*R*,12b*S*)-9-[(ethoxycarbonyl)oxy]-5-hexanoyl-7-oxo-1,2,4,4a,5,6,7,7a-octahydro-3*H*-4,12-methanobenzofuro[3,2-*e*]isoquinoline-3-carboxylate (2ab). Prepared according to the General Procedure using enone **1a** (124 mg, 0.30 mmol) and hexanal (0.18 mL, 1.50 mmol) as the radical donor, under blue LED irradiation. Purification by column chromatography (50% EtOAc/petrol) gave **2ab** (91 mg, 59%) as a white foam, as a *ca.* 1:1 mixture of rotamers. R_f = 0.41 (50% EtOAc/petrol); m.p. 181-183 °C (Et_2O); $[\alpha]_D^{20.1}$ −152 (*c* 1.00, $CHCl_3$); IR (ATR) 2928, 1760 (C=O), 1732 (C=O), 1687 (C=O), 1624 (C=O), 1496, 1442, 1423, 1370, 1312 cm^{-1}; ^{1}H NMR (400 MHz, $CDCl_3$) δ 6.98 (1H, d, *J* = 8.3 Hz, Ar**H**), 6.73 (1H, d, *J* = 8.3 Hz, Ar**H**), 4.75 (1H, s, ArOC**H**), 4.70-4.65 (0.5H, m, C**H**N, rotamer A), 4.60-4.53 (0.5H, m, C**H**N, rotamer B), 4.39-4.25 (2H, m, $OCO_2C\mathbf{H_2}$), 4.22-3.91 (3H, m, $NCO_2C\mathbf{H_2}$ and $C\mathbf{H_a}H_bN$), 2.91-2.64 (4H, m, $CH_a\mathbf{H_b}N$ and $ArC\mathbf{H_2}$ and $O{=}CCH_2C\mathbf{H}$), 2.53-2.26 (5H, m, C**H**CHN and $O{=}CC\mathbf{H_2}CH_2$ and $O{=}CC\mathbf{H_2}CH$), 2.02 (1H, td, *J* = 12.4, 5.3 Hz, $C\mathbf{H_a}H_bCH_2N$), 1.92-1.83 (1H, m, $CH_a\mathbf{H_b}CH_2N$), 1.63-1.50 (2H, m, $O{=}CCH_2C\mathbf{H_2}$), 1.38 (3H, t, *J* = 7.1 Hz, $OCO_2CH_2C\mathbf{H_3}$), 1.31-1.22 (7H, m, $NCO_2CH_2C\mathbf{H_3}$ and $C\mathbf{H_2}C\mathbf{H_2}CH_3$), 0.92-0.75 (3H, m, $(CH_2)_4C\mathbf{H_3}$); **Rotamer A** ^{13}C NMR (101 MHz, $CDCl_3$) δ 208.6 (C), 204.2 (C), 155.0 (C), 153.0 (C), 148.0 (C), 133.6 (C), 130.4 (C), 127.1 (C), 123.3 (CH), 120.6 (CH), 91.4 (CH), 65.3 (CH_2), 61.9 (CH_2), 48.4 (CH), 47.7 (CH), 46.9 (C), 43.5 (CH_2), 42.9 (CH), 42.4 (CH_2), 37.8 (CH_2), 34.9 (CH_2), 31.3 (CH_2), 29.1 (CH_2), 23.1 (CH_2), 22.5 (CH_2), 14.8 (CH_3), 14.3 (CH_3), 14.0 (CH_3); **Rotamer B** ^{13}C NMR (101 MHz, $CDCl_3$) δ 208.9 (C), 204.4 (C), 155.5 (C), 153.0 (C), 148.0 (C), 133.6 (C), 130.7 (C), 127.1 (C), 123.3 (CH), 120.6 (CH), 91.4 (CH), 65.3 (CH_2), 61.9 (CH_2), 48.6 (CH), 47.7 (CH), 46.9 (C), 43.5 (CH_2), 43.1 (CH), 42.4 (CH_2), 38.1

(CH_2), 35.0 (CH_2), 31.3 (CH_2), 29.1 (CH_2), 23.1 (CH_2), 22.5 (CH_2), 14.8 (CH_3), 14.3 (CH_3), 14.0 (CH_3); HRMS (ESI) Exact mass calculated for $[C_{28}H_{36}NO_8]^+$ $[M+H]^+$: 514.2435, found 514.2430.

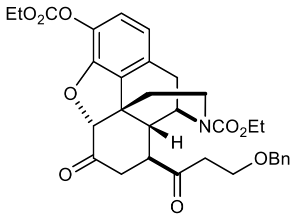

Ethyl (4*R*,4a*R*,5*S*,7a*R*,12b*S*)-5-[3-(benzyloxy)propanoyl]-9-[(ethoxycarbonyl)oxy]-7-oxo-1,2,4,4a,5,6,7,7a-octahydro-3*H*-4,12-methanobenzofuro[3,2-*e*]isoquinoline-3-carboxylate (2ac). Prepared according to the General Procedure using enone **1a** (124 mg, 0.30 mmol) and 3-(benzyloxy)propanal (0.23 mL, 1.50 mmol) as the radical donor, under blue LED irradiation. Purification by column chromatography (50% EtOAc/petrol) gave **2ac** (36 mg, 21%) as an off-white foam, as a *ca.* 1:1 mixture of rotamers. R_f = 0.21 (50% EtOAc/petrol); m.p. 158-163 °C (Et_2O); $[\alpha]_D^{20.1}$ −165 (*c* 1.00, $CHCl_3$); IR (ATR) 2929, 1762 (C=O), 1685 (C=O), 1623 (C=O), 1495, 1443, 1425, 1370, 1316, 1232 cm^{-1}; ^1H NMR (400 MHz, $CDCl_3$) δ 7.37-7.23 (5H, m, $C_6\mathbf{H}_5$), 6.96 (1H, d, *J* = 8.2 Hz, Ar**H**), 6.63 (1H, d, *J* = 8.2 Hz, Ar**H**), 4.78-4.70 (1.5H, m, ArOC**H**, and C**H**N of rotamer A), 4.67-4.60 (0.5H, m, C**H**N, rotamer B) 4.50-4.42 (2H, m, OC$\mathbf{H}_2$Ph), 4.37-4.29 (2H, m, $OCO_2C\mathbf{H}_2$), 4.22-3.93 (3H, m, $NCO_2C\mathbf{H}_2$ and C$\mathbf{H}_a$$H_b$N), 3.78-3.65 (2H, m, C$\mathbf{H}_2$OBn), 2.84-2.61 (6H, m, CH$_a$$\mathbf{H}_b$N and O=CC$\mathbf{H}_2$$CH_2$ and ArC$\mathbf{H}_2$CH and O=CCH_2C**H**), 2.56-2.35 (3H, m, O=CC$\mathbf{H}_2$CH and C**H**CHN), 2.06-1.96 (1H, m, C$\mathbf{H}_a$$H_b$$CH_2$N), 1.92-1.84 (1H, m, CH$_a$$\mathbf{H}_b$$CH_2$N), 1.39 (3H, t, *J* = 7.1 Hz, $OCO_2CH_2C\mathbf{H}_3$), 1.35-1.17 (3H, m, $NCO_2CH_2C\mathbf{H}_3$); **Rotamer A** ^{13}C NMR (101 MHz, $CDCl_3$) δ 207.6 (C), 204.0 (C), 155.4 (C), 153.0 (C), 147.9 (C), 137.8 (C), 133.5 (C), 130.9 (C), 128.6 (2 × CH), 128.0 (2 × CH), 127.9 (CH), 127.0 (C), 123.2 (CH), 120.7 (CH), 91.5 (CH), 73.6 (CH_2), 65.7 (CH_2), 65.3 (CH_2), 61.8 (CH_2), 48.5 (CH), 48.4 (CH), 46.9 (C), 42.9 (CH_2), 42.5 (CH), 41.9 (CH_2), 38.1 (CH_2), 35.1 (CH_2), 28.8 (CH_2), 14.8 (CH_3), 14.3 (CH_3); **Rotamer B** ^{13}C NMR (101 MHz, $CDCl_3$) δ 207.4 (C), 204.0 (C), 155.4 (C), 153.0 (C), 147.9 (C), 137.8 (C), 133.5 (C), 130.9 (C), 128.6 (2 × CH), 128.0 (2 × CH), 127.9 (CH), 127.0 (C), 123.2 (CH), 120.7 (CH), 91.5 (CH), 73.6 (CH_2), 65.6 (CH_2), 65.3 (CH_2), 61.8 (CH_2), 48.5 (CH), 48.4 (CH), 46.9 (C), 42.7 (CH_2), 42.5 (CH), 41.9 (CH_2), 37.9 (CH_2), 34.9 (CH_2), 28.8 (CH_2), 14.8 (CH_3), 14.3 (CH_3); HRMS (ESI) Exact mass calculated for $[C_{32}H_{36}NO_9]^+$ $[M+H]^+$: 578.2385, found 578.2382.

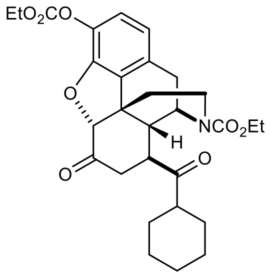

Ethyl (4*R*,4a*R*,5*S*,7a*R*,12b*S*)-5-(cyclohexanecarbonyl)-9-[(ethoxycarbonyl)oxy]-7-oxo-1,2,4,4a,5,6,7,7a-octahydro-3*H*-4,12-methanobenzofuro[3,2-*e*]isoquinoline-3-carboxylate (2ad). Prepared according to the General Procedure using enone **1a** (124 mg, 0.30 mmol) and cyclohexanecarbaldehyde (0.18 mL, 1.50 mmol) as the radical donor, under blue LED irradiation. Purification by column chromatography (50% EtOAc/petrol) gave **2ad** (109 mg, 70%) as a white foam, as a *ca.* 1:1 mixture of rotamers. R_f = 0.37

(50% EtOAc/petrol); m.p. 150-155 °C (Et_2O); $[\alpha]_D^{20.1}$ −212 (*c* 1.00, $CHCl_3$); IR 2928, 2855, 1762 (C=O), 1733 (C=O), 1690 (C=O), 1624 (C=O), 1496, 1443, 1371, 1313 cm^{-1}; ^1H NMR (400 MHz, $CDCl_3$) δ 7.02 (1H, d, *J* = 8.2 Hz, Ar**H**), 6.77 (1H, d, *J* = 8.2 Hz, Ar**H**), 4.79 (1H, s, ArOC**H**), 4.68-4.63 (0.5H, m, C**H**N, rotamer A), 4.57-4.53 (0.5H, m, C**H**N, rotamer B), 4.40-4.32 (2H, m, $OCO_2C\mathbf{H}_2$), 4.23-3.96 (3H, m, $NCO_2C\mathbf{H}_2$ and $C\mathbf{H}_aH_bN$), 2.93-2.68 (4H, m, $CH_a\mathbf{H}_bN$ and $ArC\mathbf{H}_2$ and C**H**CHN), 2.62-2.39 (3H, m, $O{=}CC\mathbf{H}_2CH$ and $O{=}CC\mathbf{H}(CH_2)_2$), 2.39-2.28 (1H, m, $O{=}CCH_2C\mathbf{H}$), 2.10-2.01 (1H, m, $C\mathbf{H}_aH_bCH_2N$), 1.95-1.86 (1H, m, $CH_a\mathbf{H}_bCH_2N$), 1.84-1.59 (4H, m, $C\mathbf{H}_2(CH_2)_3C\mathbf{H}_2$), 1.41 (3H, t, *J* = 7.1 Hz, $OCO_2CH_2C\mathbf{H}_3$), 1.35-1.23 (9H, m, $NCO_2CH_2C\mathbf{H}_3$ and $CH_2(C\mathbf{H}_2)_3CH_2$); **Rotamer A (Major)** ^{13}C NMR (101 MHz, $CDCl_3$) δ 211.7 (C), 204.5 (C), 155.3 (C), 153.0 (C), 148.0 (C), 133.6 (C), 130.8 (C), 127.1 (C), 123.4 (CH), 120.6 (CH), 91.5 (CH), 65.4 (CH_2), 61.9 (CH_2), 51.4 (CH), 48.7 (CH), 47.0 (C), 46.6 (CH), 43.0 (CH), 42.8 (CH_2), 38.1 (CH_2), 35.2 (CH_2), 29.1 (CH_2), 28.3 (CH_2), 25.7 (2 × CH_2), 25.5 (2 × CH_2), 14.8 (CH_3), 14.3 (CH_3); **Rotamer B (Minor)** ^{13}C NMR (101 MHz, $CDCl_3$) δ 211.7 (C), 204.5 (C), 155.3 (C), 153.0 (C), 148.0 (C), 133.6 (C), 130.8 (C), 127.1 (C), 123.4 (CH), 120.6 (CH), 91.5 (CH), 65.4 (CH_2), 61.9 (CH_2), 51.1 (CH), 48.6 (CH), 47.0 (C), 46.3 (CH), 43.0 (CH), 42.8 (CH_2), 38.0 (CH_2), 35.2 (CH_2), 29.1 (CH_2), 28.3 (CH_2), 25.7 (2 × CH_2), 25.5 (2 × CH_2), 14.8 (CH_3), 14.3 (CH_3); HRMS (ESI) Exact mass calculated for $[C_{29}H_{36}NO_8]^+$ $[M+H]^+$: 526.2435, found 526.2424.

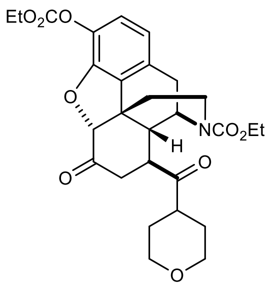

Ethyl (4*R*,4a*R*,5*S*,7a*R*,12b*S*)-9-[(ethoxycarbonyl)oxy]-7-oxo-5-(tetrahydro-2*H*-pyran-4-carbonyl)-1,2,4,4a,5,6,7,7a-octahydro-3*H*-4,12-methanobenzofuro[3,2-*e*]isoquinoline-3-carboxylate (2ae). Prepared according to the General Procedure using enone **1a** (124 mg, 0.30 mmol) and tetrahydro-2*H*-pyran-4-carbaldehyde (0.16 mL, 1.50 mmol) as the radical donor, under blue LED irradiation. Purification by column chromatography (50% EtOAc/petrol) gave **2ae** (55 mg, 35%) as a yellow foam, as a 1.5:1 mixture of rotamers. R_f = 0.19 (50% EtOAc/petrol); m.p. 161-165 °C (Et_2O); $[\alpha]_D^{20.1}$ −155 (*c* 1.00, $CHCl_3$); IR 2931, 2851, 1762 (C=O), 1732 (C=O), 1689 (C=O), 1624 (C=O), 1495, 1443, 1371, 1314 cm^{-1}; ^1H NMR (400 MHz, $CDCl_3$) δ 6.99 (1H, d, *J* = 8.3 Hz, Ar**H**), 6.74 (1H, d, *J* = 8.3 Hz, Ar**H**), 4.77 (1H, s, ArOC**H**), 4.65-4.59 (0.6H, m, C**H**N, rotamer A), 4.54-4.46 (0.4H, m, C**H**N, rotamer B), 4.38-4.27 (2H, m, $OCO_2C\mathbf{H}_2$), 4.21-4.05 (2H, m, $NCO_2C\mathbf{H}_2$), 4.03-3.87 (3H, m, $C\mathbf{H}_aH_bN$ and $(C\mathbf{H}_aH_b)_2O$), 3.47-3.29 (2H, m, $(CH_a\mathbf{H}_b)_2O$), 2.86-2.67 (4H, m, $ArC\mathbf{H}_2$ and $CH_a\mathbf{H}_bN$ and C**H**CHN), 2.61-2.35 (4H, m, $O{=}CC\mathbf{H}_2$ and $O{=}CCH_2C\mathbf{H}$ and $O{=}CC\mathbf{H}(CH_2)_2$), 2.10-1.97 (1H, m, $C\mathbf{H}_aH_bCH_2N$), 1.93-1.83 (1H, m, $CH_a\mathbf{H}_bCH_2N$), 1.70-1.59 (4H, m, $(C\mathbf{H}_aH_b)_2CH_2O$), 1.38 (3H, t, *J* = 7.1 Hz, $OCO_2CH_2C\mathbf{H}_3$), 1.30-1.22 (3H, m, $NCO_2CH_2C\mathbf{H}_3$); **Rotamer A (Major)** ^{13}C NMR (101 MHz, $CDCl_3$) δ 209.5 (C), 204.1 (C),

155.4 (C), 152.9 (C), 148.0 (C), 133.6 (C), 130.4 (C), 127.0 (C), 123.4 (CH), 120.6 (CH), 91.4 (CH), 67.0 (2 × CH_2), 65.4 (CH_2), 61.9 (CH_2), 48.6 (CH), 48.1 (CH), 47.0 (C), 45.9 (CH), 43.0 (CH), 42.7 (CH_2), 38.0 (CH_2), 35.1 (CH_2), 29.0 (CH_2), 27.9 (2 × CH_2), 14.7 (CH_3), 14.3 (CH_3); **Rotamer B (Minor)** ^{13}C NMR (101 MHz, $CDCl_3$) δ 209.5 (C), 203.9 (C), 155.1 (C), 152.9 (C), 148.0 (C), 133.6 (C), 130.2 (C), 127.0 (C), 123.4 (CH), 120.6 (CH), 91.4 (CH), 67.0 (2 × CH_2), 65.4 (CH_2), 61.9 (CH_2), 48.6 (CH), 48.1 (CH), 47.0 (C), 45.9 (CH), 43.0 (CH), 42.7 (CH_2), 37.8 (CH_2), 35.1 (CH_2), 29.2 (CH_2), 27.7 (2 × CH_2), 14.7 (CH_3), 14.3 (CH_3); HRMS (ESI) Exact mass calculated for $[C_{28}H_{34}NO_9]^+$ $[M+H]^+$: 528.2228, found 528.2216.

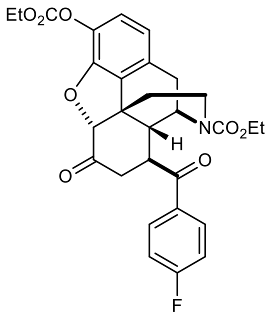

Ethyl (4*R*,4a*R*,5*S*,7a*R*,12b*S*)-9-[(ethoxycarbonyl)oxy]-5-(4-fluorobenzoyl)-7-oxo-1,2,4,4a,5,6,7,7a-octahydro-3*H*-4,12-methanobenzofuro[3,2-*e*]isoquinoline-3-carboxylate (2af). Prepared according to the General Procedure using enone **1a** (124 mg, 0.30 mmol) and 4-fluorobenzaldehyde (0.16 mL, 1.50 mmol) as the radical donor, under blue LED irradiation. Purification by column chromatography (50% EtOAc/petrol) gave **2af** (67 mg, 42%) as a white foam, as a 1.5:1 mixture of rotamers. R_f = 0.33 (50% EtOAc/petrol); m.p. 143-145 °C (Et_2O); $[\alpha]_D^{20.1}$ −206 (*c* 1.00, $CHCl_3$); IR 2935, 1760 (C=O), 1731 (C=O), 1680 (C=O), 1623 (C=O), 1595, 1496, 1444, 1425, 1371 cm^{-1}; ^{1}H NMR (400 MHz, $CDCl_3$) δ 7.92-7.84 (2H, m, Ar**H**), 7.19-7.08 (2H, m, Ar**H**), 7.01 (1H, d, *J* = 8.2 Hz, Ar**H**), 6.73 (1H, d, *J* = 8.2 Hz, Ar**H**), 4.83 (1H, s, ArOC**H**), 4.66-4.59 (0.6H, m, C**H**N, rotamer A), 4.56-4.49 (0.4H, m, C**H**N, rotamer B), 4.37-4.29 (2H, m, $OCO_2C\mathbf{H_2}$), 4.19-3.93 (3H, m, $NCO_2C\mathbf{H_2}$ and $C\mathbf{H_a}H_bN$), 3.31-3.17 (1H, m, $O{=}CCH_2C\mathbf{H}$), 3.05-2.94 (1H, m, C**H**CHN), 2.81-2.57 (4H, m, $CH_a\mathbf{H_b}N$ and $ArC\mathbf{H_2}$ and $O{=}CC\mathbf{H_a}H_b$), 2.56-2.47 (1H, m, $O{=}CCH_a\mathbf{H_b}$), 2.09 (1H, td, *J* = 12.6, 5.3 Hz, $C\mathbf{H_a}H_bCH_2N$), 1.96-1.86 (1H, m, $CH_a\mathbf{H_b}CH_2N$), 1.39 (3H, t, *J* = 7.1 Hz, $OCO_2CH_2C\mathbf{H_3}$), 1.29-1.22 (3H, m, $NCO_2CH_2C\mathbf{H_3}$); **Rotamer A (Major)** ^{13}C NMR (101 MHz, $CDCl_3$) δ 204.5 (C), 196.7 (C), 166.5 (d, *J* = 257.4 Hz, C), 155.0 (C), 153.0 (C), 148.0 (C), 133.6 (C), 132.1 (C), 131.3 (2 × CH), 130.4 (C), 127.1 (C), 123.4 (CH), 120.64 (CH), 116.5 (d, *J* = 22.0 Hz, 2 × CH), 91.5 (CH), 65.4 (CH_2), 61.8 (CH_2), 48.3 (CH), 47.1 (C), 43.4 (CH), 43.3 (CH_2), 42.2 (CH), 37.9 (CH_2), 35.2 (CH_2), 28.9 (CH_2), 14.7 (CH_3), 14.3 (CH_3); **Rotamer B (Minor)** ^{13}C NMR (101 MHz, $CDCl_3$) δ 204.5 (C), 196.9 (C), 166.5 (d, *J* = 257.4 Hz, C), 155.4 (C), 153.0 (C), 148.0 (C), 133.6 (C), 132.1 (C), 131.3 (2 × CH), 130.7 (C), 127.1 (C), 123.4 (CH), 120.6 (CH), 116.5 (d, *J* = 22.0 Hz, 2 × CH), 91.5 (CH), 65.4 (CH_2), 61.8 (CH_2), 48.5 (CH), 47.1 (C), 43.4 (CH), 43.3 (CH_2), 42.4 (CH), 38.1 (CH_2), 35.2 (CH_2), 29.0 (CH_2), 14.7 (CH_3), 14.3 (CH_3); ^{19}F NMR (276 MHz, $CDCl_3$) δ –102.8; HRMS (ESI) Exact mass calculated for $[C_{29}H_{29}FNO_8]^+$ $[M+H]^+$: 538.1872, found 538.1870.

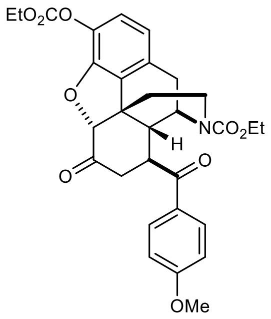

Ethyl (4*R*,4a*R*,5*S*,7a*R*,12b*S*)-9-[(ethoxycarbonyl)oxy]-5-(4-methoxybenzoyl)-7-oxo-1,2,4,4a,5,6,7,7a-octahydro-3*H*-4,12-methanobenzofuro[3,2-*e*]isoquinoline-3-carboxylate (2ag). Prepared according to the General Procedure using enone **1a** (124 mg, 0.30 mmol) and 4-methoxybenzaldehyde (0.18 mL, 1.50 mmol) as the radical donor, under blue LED irradiation. Purification by column chromatography (50% EtOAc/petrol) gave **2ag** (102 mg, 62%) as a white foam, as a 1.5:1 mixture of rotamers. R_f = 0.25 (50% EtOAc/petrol); m.p. 198-201 °C (Et_2O); $[\alpha]_D^{20.1}$ −175 (*c* 1.00, $CHCl_3$); IR 2923, 2853, 1761 (C=O), 1731 (C=O), 1688 (C=O), 1669 (C=O), 1598, 1573, 1511, 1496 cm^{-1}; ^1H NMR (400 MHz, $CDCl_3$) δ 7.85 (2H, d, *J* = 8.9 Hz, Ar**H**), 7.04 (1H, d, *J* = 8.3 Hz, Ar**H**), 6.98-6.90 (2H, m, Ar**H**), 6.76 (1H, d, *J* = 8.3 Hz, Ar**H**), 4.85 (1H, s, ArOC**H**), 4.69-4.62 (0.6H, m, C**H**N, rotamer A), 4.58-4.53 (0.4H, m, C**H**N, rotamer B), 4.37 (2H, q, *J* = 7.1 Hz, $OCO_2C\mathbf{H}_2$), 4.22-3.96 (3H, m, $NCO_2C\mathbf{H}_2$ and $C\mathbf{H}_aH_bN$), 3.89 (3H, s, $OC\mathbf{H}_3$), 3.34-3.21 (1H, m, C**H**CHN), 3.07-2.98 (1H, m, $O{=}CCH_2C\mathbf{H}$), 2.84-2.61 (4H, m, $CH_a\mathbf{H}_bN$ and $ArC\mathbf{H}_2$ and $O{=}CC\mathbf{H}_aH_b$), 2.58-2.48 (1H, m, $O{=}CCH_a\mathbf{H}_b$), 2.12 (1H, dt, *J* = 12.7, 6.4 Hz, $C\mathbf{H}_aH_bCH_2N$), 1.99-1.90 (1H, m, $CH_a\mathbf{H}_bCH_2N$), 1.43 (3H, t, *J* = 7.1 Hz, $OCO_2CH_2C\mathbf{H}_3$), 1.34-1.24 (3H, m, $NCO_2CH_2C\mathbf{H}_3$); **Rotamer A (Major)** ^{13}C NMR (101 MHz, $CDCl_3$) δ 205.0 (C), 196.8 (C), 164.6 (C), 155.3 (C), 148.1 (C), 133.6 (C), 131.0 (2 × CH), 130.7 (C), 128.7 (C), 127.3 (C), 123.3 (CH), 121.8 (C), 120.6 (CH), 114.4 (2 × CH), 91.6 (CH), 65.4 (CH_2), 61.8 (CH_2), 55.7 (CH_3), 48.4 (CH), 47.1 (C), 43.7 (CH), 43.6 (CH_2), 41.8 (CH), 38.1 (CH_2), 35.2 (CH_2), 29.1 (CH_2), 14.8 (CH_3), 14.3 (CH_3); **Rotamer B (Minor)** ^{13}C NMR (101 MHz, $CDCl_3$) δ 204.8 (C), 196.7 (C), 164.6 (C), 155.3 (C), 148.1 (C), 133.6 (C), 130.9 (2 × CH), 130.7 (C), 128.7 (C), 127.3 (C), 123.3 (CH), 121.8 (C), 120.6 (CH), 114.4 (2 × CH), 91.6 (CH), 65.4 (CH_2), 61.8 (CH_2), 55.7 (CH_3), 48.4 (CH), 47.1 (C), 43.7 (CH), 43.6 (CH_2), 41.8 (CH), 37.9 (CH_2), 35.2 (CH_2), 29.1 (CH_2), 14.8 (CH_3), 14.3 (CH_3); HRMS (ESI) Exact mass calculated for $[C_{30}H_{32}NO_9]^+$ $[M+H]^+$: 550.2072, found 550.2056.

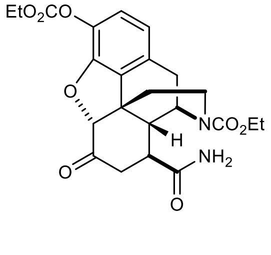

Ethyl (4*R*,4a*R*,5*S*,7a*R*,12b*S*)-5-carbamoyl-9-[(ethoxycarbonyl)oxy]-7-oxo-1,2,4,4a,5,6,7,7a-octahydro-3*H*-4,12-methanobenzofuro[3,2-*e*]isoquinoline-3-carboxylate (2ah). Prepared according to the General Procedure using enone **1a** (124 mg, 0.30 mmol) and formamide (0.06 mL, 1.50 mmol) as the radical donor, under blue LED irradiation. Purification by column chromatography (90% EtOAc/CH_2Cl_2) gave **2ah** as a white foam (73 mg, 53%), as a 2.3:1 mixture of rotamers. R_f = 0.16 (90% EtOAc/CH_2Cl_2); m.p. 182-184 °C (Et_2O); $[\alpha]_D^{20.1}$ −149 (*c* 1.00, $CHCl_3$); IR 3350 (NH), 3203 (NH), 2980, 1762 (C=O), 1731 (C=O), 1667 (C=O), 1623 (C=O), 1496, 1442, 1371 cm^{-1}; ^1H NMR (400 MHz, $CDCl_3$) δ 6.90 (1H, d, *J* = 8.1 Hz, Ar**H**), 6.78 (0.7H, s, $N\mathbf{H}_aH_b$,

rotamer A), 6.65 (1H, d, J = 8.1 Hz, Ar**H**), 6.57 (0.3H, s, N**H**$_a$H$_b$, rotamer B), 6.25 (0.7H, s, NH$_a$**H**$_b$, rotamer A), 5.68 (0.3H, s, NH$_a$**H**$_b$, rotamer B), 4.75-4.60 (2H, m, C**H**N and ArOC**H**), 4.34-4.20 (2H, m, OCO$_2$C**H**$_2$), 4.17-4.02 (1H, m, C**H**$_a$H$_b$N), 3.99-3.74 (2H, m, NCO$_2$C**H**$_2$), 3.14-2.84 (1H, m, ArC**H**$_a$H$_b$), 2.80-2.56 (4H, m, ArCH$_a$**H**$_b$ and CH$_a$**H**$_b$N and O=CC**H**$_a$H$_b$ and O=CCH$_2$C**H**), 2.50-2.37 (1H, m, O=CCH$_a$**H**$_b$), 2.18-1.96 (2H, m C**H**CHN and C**H**$_a$H$_b$CH$_2$N), 1.86-1.79 (1H, m, CH$_a$**H**$_b$CH$_2$N), 1.36 (3H, t, J = 7.1 Hz, OCO$_2$CH$_2$C**H**$_3$), 1.30-1.09 (3H, m, NCO$_2$CH$_2$C**H**$_3$); **Rotamer A (Major)** ^{13}C NMR (101 MHz, CDCl$_3$) δ 205.7 (C), 173.4 (C), 155.5 (C), 153.0 (C), 147.9 (C), 133.3 (C), 131.0 (C), 127.3 (C), 123.1 (CH), 120.4 (CH), 91.3 (CH), 65.5 (CH$_2$), 62.0 (CH$_2$), 48.7 (CH), 46.7 (C), 43.1 (CH$_2$), 43.0 (CH), 42.5 (CH), 37.9 (CH$_2$), 34.8 (CH$_2$), 28.6 (CH$_2$), 14.6 (CH$_3$), 14.2 (CH$_3$); **Rotamer B (Major)** ^{13}C NMR (101 MHz, CDCl$_3$) δ 205.7 (C), 172.9 (C), 155.2 (C), 153.0 (C), 147.9 (C), 133.3 (C), 131.0 (C), 127.3 (C), 123.1 (CH), 120.4 (CH), 91.3 (CH), 65.5 (CH$_2$), 61.9 (CH$_2$), 48.7 (CH), 46.7 (C), 43.1 (CH$_2$), 43.0 (CH), 42.5 (CH), 37.9 (CH$_2$), 34.6 (CH$_2$), 29.0 (CH$_2$), 14.8 (CH$_3$), 14.2 (CH$_3$); HRMS (ESI) Exact mass calculated for [C$_{23}$H$_{27}$N$_2$O$_8$]$^+$ [M+H]$^+$: 459.1762, found 459.1747.

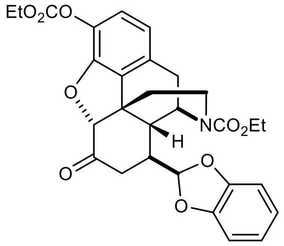

Ethyl (4*R*,4a*R*,5*S*,7a*R*,12b*S*)-5-(benzo[*d*][1,3]dioxol-2-yl)-9-[(ethoxycarbonyl)oxy]-7-oxo-1,2,4,4a,5,6,7,7a-octahydro-3*H*-4,12-methanobenzofuro[3,2-*e*]isoquinoline-3-carboxylate (2ai). Prepared according to the General Procedure using enone **1a** (124 mg, 0.30 mmol) and 1,3-benzodioxole (0.17 mL, 1.50 mmol) as the radical donor, under blue LED irradiation. Purification by column chromatography (50% EtOAc/petrol) gave **2ai** (95.0 mg, 59%) as a white foam, as a *ca.* 1:1 mixture of rotamers. R$_f$ = 0.27 (50% EtOAc/petrol); m.p. 149-154 °C (Et$_2$O); $[\alpha]_D^{20.1}$ −199 (*c* 1.00, CHCl$_3$); IR (ATR) 2922, 1761 (C=O), 1733 (C=O), 1684 (C=O), 1625, 1483, 1442, 1371, 1355, 1312 cm^{-1}; ^{1}H NMR (400 MHz, CDCl$_3$) δ 6.97 (1H, d, J = 8.3 Hz, Ar**H**), 6.86-6.75 (4H, m, Ar**H**), 6.73 (1H, d, J = 8.3 Hz, Ar**H**), 6.25-6.20 (0.5H, m, C**H**O$_2$, rotamer A), 6.11-6.06 (0.5H, m, C**H**O$_2$, rotamer B), 5.26-5.21 (0.5H, m, C**H**N, rotamer A), 5.10-5.05 (0.5H, m, C**H**N, rotamer B), 4.74 (1H, s, ArOC**H**), 4.40-4.24 (2H, m, OCO$_2$C**H**$_2$), 4.21-3.97 (3H, m, NCO$_2$C**H**$_2$ and C**H**$_a$H$_b$N), 3.07-2.95 (1H, m, ArC**H**$_a$H$_b$), 2.87-2.72 (2H, m, ArCH$_a$**H**$_b$ and CH$_a$**H**$_b$N), 2.72-2.58 (2H, m, O=CC**H**$_a$H$_b$CH and C**H**CHN), 2.50-2.30 (1H, m, O=CCH$_a$**H**$_b$CH), 2.08-1.87 (3H, m, C**H**$_2$CH$_2$N and O=CCH$_2$C**H**), 1.37 (3H, t, J = 7.1 Hz, OCO$_2$CH$_2$C**H**$_3$), 1.32-1.10 (3H, m, NCO$_2$CH$_2$C**H**$_3$); **Rotamer A** ^{13}C NMR (101 MHz, CDCl$_3$) δ 204.5 (C), 155.1 (C), 152.9 (C), 148.0 (C), 147.2 (C), 147.0 (C), 133.5 (C), 130.9 (C), 127.0 (C), 123.3 (CH), 122.1 (2 × CH), 120.7 (CH), 110.8 (CH), 108.8 (2 × CH), 91.6 (CH), 65.3 (CH$_2$), 61.9 (CH$_2$), 48.3 (CH), 47.2 (C), 42.0 (CH), 40.3 (CH), 38.7 (CH$_2$), 37.9 (CH$_2$), 35.1 (CH$_2$), 28.9 (CH$_2$), 14.7 (CH$_3$), 14.3 (CH$_3$); **Rotamer B** ^{13}C NMR (101 MHz, CDCl$_3$) δ 204.6 (C), 155.6 (C), 152.9 (C), 148.0 (C), 147.2 (C), 147.0 (C), 133.5 (C), 130.9 (C), 127.0

(C), 123.3 (CH), 122.1 (2 × CH), 120.7 (CH), 110.8 (CH), 108.8 (2 × CH), 91.6 (CH), 65.3 (CH_2), 61.9 (CH_2), 48.6 (CH), 47.2 (C), 42.2 (CH), 40.3 (CH), 39.8 (CH_2), 38.1 (CH_2), 35.2 (CH_2), 29.1 (CH_2), 14.7 (CH_3), 14.3 (CH_3); HRMS (ESI) Exact mass calculated for $[C_{29}H_{30}NO_9]^+$ $[M+H]^+$: 536.1915, found 536.1897.

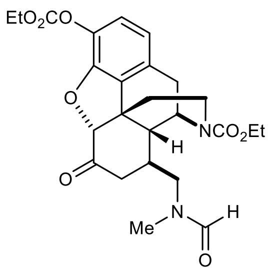

Ethyl (4*R*,4a*R*,5*S*,7a*R*,12b*S*)-9-[(ethoxycarbonyl)oxy]-5-[(*N*-methylformamido)methyl]-7-oxo-1,2,4,4a,5,6,7,7a-octahydro-3*H*-4,12-methanobenzofuro[3,2-*e*]isoquinoline-3-carboxylate (2aj). Prepared according to the General Procedure using enone **1a** (124 mg, 0.30 mmol) and DMF (0.12 mL, 1.50 mmol) as the radical donor, under blue LED irradiation. Purification by column chromatography (10% MeOH/EtOAc) gave **2aj** (45 mg, 31%) as an off-white solid, as a complex mixture of rotamers. R_f = 0.25 (10% MeOH/EtOAc); m.p. 136-137 °C (Et_2O); $[\alpha]_D^{20.1}$ −159 (*c* 1.00, $CHCl_3$); IR (ATR) 2929, 1762 (C=O), 1730 (C=O), 1667 (C=O), 1625 (C=O), 1495, 1443, 1425, 1371, 1318 cm^{-1}; ^1H NMR (400 MHz, DMSO-d_6, 120 °C) δ 8.04 (1H, s, O=C**H**), 6.99 (1H, d, *J* = 8.3 Hz, Ar**H**), 6.78 (1H, d, *J* = 8.3 Hz, Ar**H**), 5.01 (1H, s, ArOC**H**), 4.94 (1H, s, C**H**N), 4.29 (2H, q, *J* = 7.1 Hz, $OCO_2C\mathbf{H_2}$), 4.15 (2H, q, *J* = 7.1 Hz, $NCO_2C\mathbf{H_2}$), 3.96 (1H, dd, *J* = 13.9, 5.3 Hz, $C\mathbf{H}_aH_bNCO_2$), 3.56-3.30 (2H, m, $C\mathbf{H_2}NCH_3$), 3.08-2.82 (4H, m, $ArC\mathbf{H}_aH_b$ and $NC\mathbf{H_3}$), 2.79-2.61 (2H, m, $ArCH_a\mathbf{H}_b$ and $CH_a\mathbf{H}_bNCO_2$), 2.48-2.34 (2H, m, $O{=}CC\mathbf{H}_aH_b$ and C**H**CHN), 2.23-2.03 (2H, m, $O{=}CCH_a\mathbf{H}_b$ and $C\mathbf{H}_aH_bCH_2N$), 1.67 (1H, ddd, *J* = 12.7, 4.0, 1.5 Hz, $CH_a\mathbf{H}_bCH_2N$), 1.61-1.44 (1H, m, $C\mathbf{H}CH_2N$), 1.33 (3H, t, *J* = 7.1 Hz, $OCO_2CH_2C\mathbf{H_3}$), 1.26 (3H, t, *J* = 7.1 Hz, $NCO_2CH_2C\mathbf{H_3}$); ^{13}C NMR (101 MHz, DMSO-d_6, 120 °C) δ 204.3 (C), 162.4 (CH), 154.1 (C), 151.4 (C), 146.9 (C), 132.2 (C), 130.5 (C), 127.1 (C), 121.7 (CH), 119.3 (CH), 90.8 (CH), 64.2 (CH_2), 60.3 (CH_2), 50.2 (CH), 47.6 (C), 46.4 (CH_2), 45.9 (CH), 43.6 (CH_2), 42.4 (CH_2), 41.8 (CH_2), 37.3 (CH_3), 33.9 (CH), 27.6 (CH_2), 13.8 (CH_3), 13.2 (CH_3); HRMS (ESI) Exact mass calculated for $[C_{25}H_{31}N_2O_8]^+$ $[M+H]^+$: 487.2075, found 487.2068.

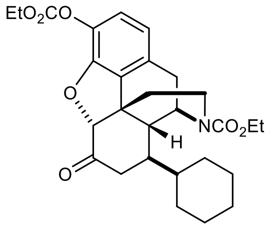

Ethyl (4*R*,4a*R*,5*R*,7a*R*,12b*S*)-5-cyclohexyl-9-[(ethoxycarbonyl)oxy]-7-oxo-1,2,4,4a,5,6,7,7a-octahydro-3*H*-4,12-methanobenzofuro[3,2-*e*]isoquinoline-3-carboxylate (2ak). Prepared according to a slight modification of General Procedure A using enone **1a** (124 mg, 0.30 mmol) and cyclohexane (0.32 mL, 3.00 mmol) as the radical donor under blue LED irradiation, but with the addition of DCE (0.2 mL) to solubilize the reaction mixture. Purification by column chromatography (50% EtOAc/petrol) gave **2ak** (45 mg, 30%) as a white foam, as a 1.5:1 mixture of rotamers. R_f = 0.31 (50% EtOAc/petrol); m.p. 163-167 °C (Et_2O); $[\alpha]_D^{20.1}$ −184 (*c* 1.00, $CHCl_3$); IR (ATR) 2923, 2852, 1762 (C=O), 1729 (C=O), 1687 (C=O), 1496, 1443, 1371, 1318,

1233 cm^{-1}; ^{1}H NMR (400 MHz, $CDCl_3$) δ 6.97 (1H, d, *J* = 8.2 Hz, Ar**H**), 6.72 (1H, d, *J* = 8.2 Hz, Ar**H**), 5.08-4.98 (0.6H, m, C**H**N, rotamer A), 4.93-4.89 (0.4H, m, C**H**N, rotamer B), 4.73 (1H, s, ArOC**H**), 4.39-4.31 (2H, m, $CO_2C\mathbf{H}_2$), 4.24-3.96 (3H, m, $NCO_2C\mathbf{H}_2$ and $C\mathbf{H}_aH_bN$), 2.92-2.72 (3H, m, $CH_a\mathbf{H}_bN$ and $ArC\mathbf{H}_2$), 2.44-2.29 (2H, m, $O{=}CC\mathbf{H}_aH_b$ and C**H**CHN), 2.19-2.08 (1H, m, $O{=}CCH_a\mathbf{H}_b$), 1.98 (1H, td, *J* = 12.4, 5.2 Hz, $C\mathbf{H}_aH_bCH_2N$), 1.92-1.87 (1H, m, $CH_a\mathbf{H}_bCH_2N$), 1.86-1.50 (2H, m, $O{=}CCH_2C\mathbf{H}$ and $C\mathbf{H}(CH_2)_2$), 1.40 (3H, t, *J* = 7.1 Hz, $OCO_2CH_2C\mathbf{H}_3$), 1.37-1.20 (13H, m, $NCO_2CH_2C\mathbf{H}_3$ and $CH(C\mathbf{H}_2)_5$); **Rotamer A (Major)** ^{13}C NMR (101 MHz, $CDCl_3$) δ 206.7 (C), 155.8 (C), 153.1 (C), 148.0 (C), 133.4 (C), 130.9 (C), 127.8 (C), 122.9 (CH), 120.4 (CH), 92.1 (CH), 65.3 (CH_2), 61.8 (CH_2), 48.1 (CH), 47.5 (C), 43.5 (CH), 41.2 (CH_2), 40.9 (CH), 38.1 (CH_2), 37.8 (CH), 35.5 (CH_2), 28.7 (CH_2), 26.9 (CH_2), 26.6 (2 × CH), 26.4 (CH_2), 14.8 (CH_3), 14.3 (CH_3); **Rotamer B (Minor)** ^{13}C NMR (101 MHz, $CDCl_3$) δ 206.7 (C), 155.8 (C), 153.1 (C), 148.0 (C), 133.4 (C), 131.1 (C), 127.8 (C), 122.9 (CH), 120.4 (CH), 92.1 (CH), 65.3 (CH_2), 61.8 (CH_2), 48.1 (CH), 47.5 (C), 44.0 (CH), 41.2 (CH_2), 40.9 (CH), 38.4 (CH_2), 37.4 (CH), 35.5 (CH_2), 28.7 (CH_2), 26.9 (CH_2), 26.6 (2 × CH), 26.4 (CH_2), 14.8 (CH_3), 14.3 (CH_3); HRMS (ESI) Exact mass calculated for $[C_{28}H_{36}NO_7]^+$ $[M+H]^+$: 498.2486, found 498.2474.

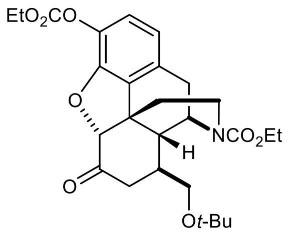

Ethyl (4*R*,4a*R*,5*S*,7a*R*,12b*S*)-5-(*tert*-butoxymethyl)-9-[(ethoxycarbonyl)oxy]-7-oxo-1,2,4,4a,5,6,7,7a-octahydro-3*H*-4,12-methanobenzofuro[3,2-*e*]isoquinoline-3-carboxylate (2al). Prepared according to the General Procedure using enone **1a** (124 mg, 0.30 mmol) and TBME (0.18 mL, 1.50 mmol) as the radical donor, under UV irradiation. Purification by column chromatography (50% EtOAc/petrol) gave **2al** (51 mg, 34%) as an off-white foam, as a *ca* 1:1 mixture of rotamers. R_f = 0.41 (50% EtOAc/petrol); m.p. 170-175 °C (Et_2O); $[\alpha]_D^{20.1}$ −198 (*c* 1.00, $CHCl_3$); IR 2974, 1763 (C=O), 1730 (C=O), 1688 (C=O), 1495, 1443, 1425, 1367, 1317, 1232 cm^{-1}; ^{1}H NMR (400 MHz, $CDCl_3$) δ 6.92 (1H, d, *J* = 8.2 Hz, Ar**H**), 6.68 (1H, d, *J* = 8.2 Hz, Ar**H**), 5.12-5.07 (0.5H, m, C**H**N, rotamer A), 5.05-4.99 (0.5H, m, C**H**N, rotamer B), 4.68 (1H, s, ArOC**H**), 4.38-4.23 (2H, m, $OCO_2C\mathbf{H}_2$), 4.23-3.92 (3H, m, $NCO_2C\mathbf{H}_2$ and $C\mathbf{H}_aH_bN$), 3.47-3.20 (2H, m, $C\mathbf{H}_2OC(CH_3)_3$), 2.92 (1H, dd, *J* = 18.7, 5.8 Hz, $ArC\mathbf{H}_aH_b$), 2.84-2.66 (2H, m, $ArC\mathbf{H}_aH_b$ and $CH_a\mathbf{H}_bN$), 2.47-2.24 (3H, m, $O{=}CC\mathbf{H}_2$ and C**H**CHN), 2.02-1.91 (1H, m, $C\mathbf{H}_aH_bCH_2N$), 1.86 (1H, td, *J* = 12.0, 3.7 Hz, $CH_a\mathbf{H}_bCH_2N$), 1.53-1.42 (1H, m, $C\mathbf{H}CH_2O$), 1.36 (3H, t, *J* = 7.2 Hz, $OCO_2CH_2C\mathbf{H}_3$), 1.31-1.22 (3H, m, $NCO_2CH_2C\mathbf{H}_3$), 1.17 (9H, s, $OC(C\mathbf{H}_3)_3$); **Rotamer A** ^{13}C NMR (101 MHz, $CDCl_3$) δ 206.3 (C), 155.6 (C), 153.0 (C), 148.0 (C), 133.3 (C), 131.4 (C), 127.7 (C), 122.8 (CH), 120.4 (CH), 91.8 (CH), 73.1 (C), 65.2 (CH_2), 62.8 (CH_2), 61.7 (CH_2), 48.7 (CH), 47.2 (C), 44.1 (CH), 43.8 (CH_2), 38.2 (CH_2), 36.9 (CH), 35.2 (CH_2), 29.2 (CH_2), 27.5 (3 × CH_3), 14.9 (CH_3), 14.3 (CH_3); **Rotamer B**

^{13}C NMR (101 MHz, $CDCl_3$) δ 206.3 (C), 155.3 (C), 153.0 (C), 148.0 (C), 133.3 (C), 131.2 (C), 127.7 (C), 122.8 (CH), 120.3 (CH), 91.8 (CH), 73.0 (C), 65.2 (CH_2), 62.6 (CH_2), 61.7 (CH_2), 48.5 (CH), 47.2 (C), 44.1 (CH), 43.8 (CH_2), 38.0 (CH_2), 36.7 (CH), 35.0 (CH_2), 28.9 (CH_2), 27.5 (3 × CH_3), 14.8 (CH_3), 14.3 (CH_3); HRMS (ESI) Exact mass calculated for $[C_{27}H_{36}NO_8]^+$ $[M+H]^+$: 502.2435, found 502.2419.

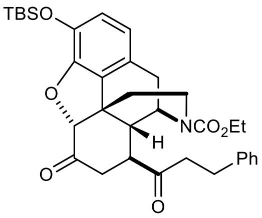

Ethyl (4*R*,4a*R*,5*S*,7a*R*,12b*S*)-9-[(*tert*-butyldimethylsilyl)oxy]-7-oxo-5-(3-phenylpropanoyl)-1,2,4,4a,5,6,7,7a-octahydro-3*H*-4,12-methanobenzofuro[3,2-*e*]isoquinoline-3-carboxylate (2ba). Prepared according to a slight modification of the General Procedure using enone **1b** (59 mg, 0.13 mmol), TBADT (22 mg, 0.0065 mmol), MeCN (0.33 mL), and 3-phenylpropanal (0.09 mL, 0.65 mmol) as the radical donor, under blue LED irradiation. Purification by column chromatography (25% EtOAc/petrol) gave **2ba** (64 mg, 36%) as a white foam, as a *ca.* 1:1 mixture of rotamers. R_f = 0.37 (50% EtOAc/petrol); m.p. 158-164 °C (Et_2O); $[\alpha]_D^{20.1}$ −201 (*c* 1.00, $CHCl_3$); IR (ATR) 2928, 2856, 1688 (C=O), 1633 (C=O), 1606, 1497, 1470, 1426, 1376, 1315 cm^{-1}; ^{1}H NMR (400 MHz, $CDCl_3$) δ 7.29-7.11 (5H, m, $C_6\mathbf{H}_5$), 6.68 (1H, d, *J* = 8.1 Hz, Ar**H**), 6.56 (1H, d, *J* = 8.1 Hz, Ar**H**), 4.69-4.65 (0.5H, m, C**H**N, rotamer A), 4.63 (1H, s, ArOC**H**), 4.53-4.48 (0.5H, m, C**H**N, rotamer B), 4.25-4.10 (2H, m, $CO_2C\mathbf{H}_2$), 4.10-3.90 (1H, m, $C\mathbf{H}_aH_bN$), 2.95-2.87 (2H, m, $O{=}CC\mathbf{H}_2CH_2$), 2.86-2.60 (6H, m, $C\mathbf{H}_2Ph$ and $CH_a\mathbf{H}_bN$ and $ArC\mathbf{H}_2$ and $O{=}CCH_2C\mathbf{H}$), 2.43-2.24 (3H, m, $O{=}CC\mathbf{H}_2CH$ and C**H**CHCN), 1.96 (1H, td, *J* = 12.5, 5.3 Hz, $C\mathbf{H}_aH_bCH_2N$), 1.86-1.76 (1H, m, $CH_a\mathbf{H}_bCH_2N$), 1.31-1.24 (3H, m, $CH_2C\mathbf{H}_3$), 1.01 (9H, s, $C(C\mathbf{H}_3)_3$), 0.29 (3H, s, $Si(C\mathbf{H}_3)_a$), 0.22 (3H, s, $Si(C\mathbf{H}_3)_b$); **Rotamer A** ^{13}C NMR (101 MHz, $CDCl_3$) δ 207.8 (C), 204.8 (C), 155.5 (C), 147.2 (C), 140.5 (C), 138.6 (C), 128.7 (2 × CH), 128.4 (2 × CH), 126.4 (CH), 125.9 (C), 125.2 (C), 123.1 (CH), 120.5 (CH), 90.6 (CH), 61.8 (CH_2), 48.8 (CH), 48.3 (CH), 47.0 (C), 44.9 (CH_2), 42.9 (CH), 42.2 (CH_2), 38.2 (CH_2), 35.2 (CH_2), 29.5 (CH_2), 28.6 (CH_2), 25.8 (3 × CH_3), 18.4 (C), 14.8 (CH_3), −4.4 (CH_3), −4.6 (CH_3); **Rotamer B** ^{13}C NMR (101 MHz, $CDCl_3$) δ 207.8 (C), 204.8 (C), 155.5 (C), 147.2 (C), 140.5 (C), 138.6 (C), 128.7 (2 × CH), 128.4 (2 × CH), 126.4 (CH), 125.9 (C), 125.2 (C), 123.1 (CH), 120.5 (CH), 90.6 (CH), 61.8 (CH_2), 48.6 (CH), 48.3 (CH), 47.0 (C), 44.3 (CH_2), 42.9 (CH), 42.2 (CH_2), 38.1 (CH_2), 35.0 (CH_2), 29.5 (CH_2), 28.6 (CH_2), 25.8 (3 × CH_3), 18.4 (C), 14.8 (CH_3), −4.4 (CH_3), −4.6 (CH_3); HRMS (ESI) Exact mass calculated for $[C_{34}H_{44}NO_6Si]^+$ $[M+H]^+$: 590.2932, found 590.2916.

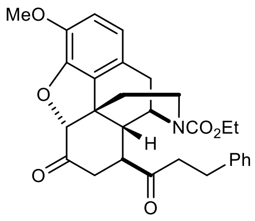

Ethyl (4*R*,4a*R*,5*S*,7a*R*,12b*S*)-9-methoxy-7-oxo-5-(3-phenylpropanoyl)-1,2,4,4a,5,6,7,7a-octahydro-3*H*-4,12-methanobenzofuro[3,2-*e*]isoquinoline-3-carboxylate (2ca). Prepared according to a slight modification of the General Procedure using enone **1c** (60 mg, 0.17 mmol), TBADT (28 mg, 0.0085 mmol), MeCN (0.43 mL), and 3-phenylpropanal (0.11 mL, 0.85 mmol) as the radical donor, under blue LED irradiation. Purification by column chromatography (40% EtOAc/petrol) gave **2ca** (52 mg, 63%) as a white foam, as a *ca.* 1:1 mixture of rotamers. R_f = 0.40 (50% EtOAc/petrol); m.p. 148-152 °C (Et_2O); $[\alpha]_D^{20.1}$ −186 (*c* 1.00, $CHCl_3$); IR (ATR) 2929, 1684 (C=O), 1601, 1502, 1426, 1375, 1315, 1275, 1259, 1234 cm^{-1}; ^1H NMR (400 MHz, $CDCl_3$) δ 7.33-7.12 (5H, m, $C_6\mathbf{H}_5$), 6.76 (1H, d, *J* = 8.2 Hz, Ar**H**), 6.69-6.61 (1H, m, Ar**H**), 4.74-4.65 (1.5H, m ArOC**H** and C**H**N, rotamer A), 4.57-4.49 (0.5H, m, rotamer B), 4.23-4.14 (2H, m, $CO_2C\mathbf{H}_2$), 4.13-3.96 (1H, m, $C\mathbf{H}_aH_bN$), 3.92 (3H, s, $OC\mathbf{H}_3$), 3.00-2.89 (2H, m, $C\mathbf{H}_2CH_2Ph$), 2.85-2.59 (6H, m, $ArC\mathbf{H}_2$ and $CH_2C\mathbf{H}_2Ph$ and $CH_a\mathbf{H}_bN$ and $O{=}CCH_2C\mathbf{H}$), 2.46-2.28 (3H, m, $O{=}CC\mathbf{H}_2CH$ and C**H**CHCN), 2.05-1.97 (1H, m, $C\mathbf{H}_aH_bCH_2N$), 1.92-1.83 (1H, m, $CH_a\mathbf{H}_bCH_2N$), 1.37-1.26 (3H, m, $CH_2C\mathbf{H}_3$); **Rotamer A (Major)** ^{13}C NMR (101 MHz, $CDCl_3$) δ 207.7 (C), 205.2 (C), 155.5 (C), 145.3 (C), 143.4 (C), 140.4 (C), 128.7 (2 × CH), 128.4 (2 × CH), 126.4 (CH), 126.0 (C), 124.8 (C), 120.7 (CH), 115.3 (CH), 91.1 (CH), 61.8 (CH_2), 56.8 (CH_3), 48.6 (CH), 48.3 (CH), 47.0 (C), 44.8 (CH_2), 42.8 (CH), 42.0 (CH_2), 38.2 (CH_2), 35.0 (CH_2), 29.5 (CH_2), 28.5 (CH_2), 14.8 (CH_3); **Rotamer B (Minor)** ^{13}C NMR (101 MHz, $CDCl_3$) δ 208.0 (C), 205.0 (C), 155.2 (C), 145.3 (C), 143.4 (C), 140.4 (C), 128.7 (2 × CH), 128.4 (2 × CH), 126.5 (CH), 126.0 (C), 124.5 (C), 120.7 (CH), 115.3 (CH), 91.1 (CH), 61.8 (CH_2), 56.8 (CH_3), 48.7 (CH), 48.4 (CH), 46.9 (C), 44.2 (CH_2), 42.8 (CH), 42.0 (CH_2), 38.0 (CH_2), 34.8 (CH_2), 29.6 (CH_2), 28.5 (CH_2), 14.8 (CH_3); HRMS (ESI) Exact mass calculated for $[C_{29}H_{32}NO_6]^+$ $[M+H]^+$: 490.2224, found 490.2223.

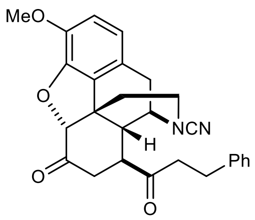

(4*R*,4a*R*,5*S*,7a*R*,12b*S*)-9-Methoxy-7-oxo-5-(3-phenylpropanoyl)-1,2,4,4a,5,6,7,7a-octahydro-3*H*-4,12-methanobenzofuro[3,2-*e*]isoquinoline-3-carbonitrile (2da). Prepared according to the General Procedure using enone **1d** (93 mg, 0.30 mmol) and 3-phenylpropanal (0.20 mL, 1.50 mmol) as the radical donor, under blue LED irradiation. Purification by column chromatography (50% EtOAc/petrol) gave **2da** (62 mg, 47%) as a white foam. R_f = 0.30 (50% EtOAc/petrol); m.p. 177-181 °C (Et_2O); $[\alpha]_D^{20.1}$ −192 (*c* 1.00, $CHCl_3$); IR (ATR) 2930, 2205 (C≡N), 1730 (C=O), 1711 (C=O), 1636, 1606, 1504, 1439, 1392, 1365 cm^{-1}; ^1H NMR (400 MHz, $CDCl_3$) δ 7.32-7.10 (5H, m, $C_6\mathbf{H}_5$), 6.77 (1H, d, *J* = 8.3 Hz, Ar**H**), 6.68 (1H, d, *J* = 8.3 Hz, Ar**H**), 4.72 (1H, s, ArOC**H**), 3.90 (3H, s, $ArOC\mathbf{H}_3$), 3.60 (1H, ddd, *J* = 5.8, 3.1, 1.2 Hz, C**H**N), 3.24 (1H, dd, *J* = 12.9, 3.8 Hz, $ArC\mathbf{H}_aH_b$), 3.05-2.76 (6H, m, $ArCH_a\mathbf{H}_b$ and $O{=}CCH_2C\mathbf{H}$ and $C\mathbf{H}_aH_bN$ and

O=CC**H**$_2$CH$_2$ and C**H**$_a$H$_b$Ph), 2.64 (1H, dt, *J* = 17.3, 7.1 Hz, CH$_a$**H**$_b$Ph), 2.58-2.46 (1H, m, CH$_a$**H**$_b$N), 2.45-2.34 (2H, m, O=CC**H**$_2$CH), 2.33-2.22 (1H, m, C**H**CHN), 2.16 (1H, td, *J* = 12.7, 5.4 Hz, C**H**$_a$H$_b$CH$_2$N), 1.91-1.84 (1H, m, CH$_a$**H**$_b$CH$_2$N); ^{13}C NMR (101 MHz, CDCl$_3$) δ 207.5 (C), 204.2 (C), 145.2 (C), 143.6 (C), 140.2 (C), 128.7 (2 × CH), 128.4 (2 × CH), 126.7 (CH), 125.2 (C), 123.6 (C), 120.8 (CH), 117.3 (C), 115.5 (CH), 90.8 (CH), 56.8 (CH$_3$), 55.1 (CH), 48.1 (CH), 45.8 (C), 44.0 (CH$_2$), 43.8 (CH$_2$), 41.7 (CH$_2$), 41.6 (CH), 33.9 (CH$_2$), 29.5 (CH$_2$), 27.7 (CH$_2$); HRMS (ESI) Exact mass calculated for [C$_{27}$H$_{27}$N$_2$O$_4$]$^+$ [M+H]$^+$: 443.1965, found 443.1960.

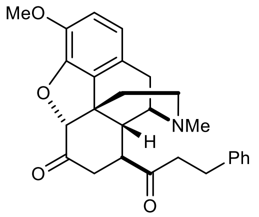

(4*R*,4a*R*,5*S*,7a*R*,12b*S*)-9-Methoxy-3-methyl-5-(3-phenylpropanoyl)-2,3,4,4a,5,6-hexahydro-1*H*-4,12-methanobenzofuro[3,2-*e*]isoquinolin-7(7a*H*)-one (2ea). Prepared according to a slight modification of the General Procedure using enone **1e** (54 mg, 0.18 mmol), TBADT (30 mg, 0.009 mmol), MeCN (0.45 mL), and 3-phenylpropanal (0.24 mL, 1.80 mmol) as the radical donor, under UV irradiation. Purification by column chromatography (5% 2 M NH$_3$[MeOH]/CH$_2$Cl$_2$) gave **2ea** as an off-white solid (17 mg, 22%). R$_f$ = 0.24 (5% 2 M NH$_3$[MeOH]/CH$_2$Cl$_2$); m.p. 130-136 °C (Et$_2$O); $[\alpha]_D^{20.1}$ −165 (*c* 1.00, CHCl$_3$); IR (ATR) 2921, 2849, 1729 (C=O), 1666 (C=O), 1598, 1573, 1507, 1438, 1377, 1313 cm^{-1}; ^{1}H NMR (400 MHz, CDCl$_3$) δ 7.29-7.24 (2H, m, Ar**H**), 7.22-7.13 (3H, m, Ar**H**), 6.71 (1H, d, *J* = 8.2 Hz, Ar**H**), 6.63 (1H, d, *J* = 8.2 Hz, Ar**H**), 4.68 (1H, s, ArOC**H**), 3.90 (3H, s, OC**H**$_3$), 2.98-2.82 (5H, m, O=CC**H**$_2$CH$_2$ and O=CCH$_2$C**H** and C**H**N and C**H**$_a$H$_b$N), 2.76-2.59 (2H, m, C**H**$_2$Ph), 2.58-2.42 (2H, m, ArC**H**$_a$H$_b$ and O=CC**H**$_a$H$_b$CH), 2.41-2.29 (5H, m, ArCH$_a$**H**$_b$ and NC**H**$_3$ and C**H**CHN), 2.23-2.07 (3H, m, CH$_a$**H**$_b$N and O=CCH$_a$**H**$_b$CH and C**H**$_a$H$_b$CH$_2$N), 1.82-1.75 (1H, m, CH$_a$**H**$_b$CH$_2$N); ^{13}C NMR (101 MHz, CDCl$_3$) δ 208.1 (C), 205.2 (C), 145.2 (C), 143.4 (C), 140.6 (C), 128.7 (2 × CH), 128.6 (CH), 126.5 (2 × CH), 120.4 (CH), 115.2 (CH), 91.2 (CH), 57.3 (CH$_3$), 57.0 (CH), 48.6 (CH), 47.0 (CH$_2$), 46.5 (C), 43.9 (CH$_2$), 42.6 (CH$_2$), 42.3 (CH$_3$), 34.9 (CH$_2$), 32.1 (C), 29.9 (CH), 29.4 (CH$_2$), 27.4 (C), 20.2 (CH$_2$); HRMS (ESI) Exact mass calculated for [C$_{27}$H$_{30}$NO$_4$]$^+$ [M+H]$^+$: 432.2169, found 432.2163.

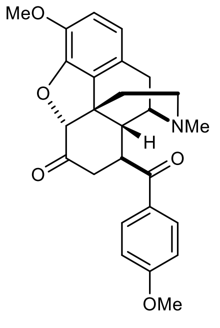

(4*R*,4a*R*,5*S*,7a*R*,12b*S*)-9-Methoxy-5-(4-methoxybenzoyl)-3-methyl-2,3,4,4a,5,6-hexahydro-1*H*-4,12-methanobenzofuro[3,2-*e*]isoquinolin-7(7a*H*)-one (2eg). Prepared according to a slight modification of the General Procedure using enone **1e** (54 mg, 0.18 mmol), TBADT (30 mg, 0.009 mmol), MeCN (0.45 mL), and 4-methoxybenzaldehyde (0.22 mL, 1.80 mmol) as the radical donor, under UV irradiation. Purification by column chromatography (5% 2 M NH$_3$[MeOH]/CH$_2$Cl$_2$) gave **2eg** as an off-white solid (19 mg, 24%). R$_f$ = 0.19 (5% 2 M NH$_3$[MeOH]/CH$_2$Cl$_2$); m.p. 143-145 °C (Et$_2$O); $[\alpha]_D^{20.1}$ −198 (*c* 1.00, CHCl$_3$); IR (ATR) 2921, 2849,

1729 (C=O), 1666 (C=O), 1598, 1573, 1507, 1438, 1377, 1313 cm^{-1}; ^{1}H NMR (400 MHz, $CDCl_3$) δ 7.80 (2H, d, *J* = 9.0 Hz, Ar**H**), 6.90 (2H, d, *J* = 8.9 Hz, Ar**H**), 6.76 (1H, d, *J* = 8.2 Hz, Ar**H**), 6.68 (1H, d, *J* = 8.2 Hz, Ar**H**), 4.78 (1H, s, ArOC**H**), 3.95 (3H, s, OC**H**3), 3.86 (3H, s, OC**H**3), 3.30 (1H, ddd, *J* = 13.3, 11.4, 2.7 Hz, O=CCH2C**H**), 3.17 (1H, dd, *J* = 11.4, 2.7 Hz, C**H**CHN), 3.07-3.02 (1H, m, C**H**N), 2.68 (1H, t, *J* = 13.3 Hz, O=CC**H**aHb), 2.61-2.56 (1H, m, C**H**aHbN), 2.47 (1H, dd, *J* = 13.3, 2.7 Hz, O=CCHa**H**b), 2.35 (3H, s, NC**H**3), 2.25-2.15 (2H, m, CHa**H**bN and ArC**H**aHb), 1.89-1.82 (1H, m, ArCHa**H**b), 1.34-1.27 (2H, m, C**H**2CH2N); ^{13}C NMR (101 MHz, $CDCl_3$) δ 206.5 (C), 197.2 (C), 164.4 (C), 149.8 (C), 145.3 (C), 143.3 (C), 130.9 (2 × CH), 128.9 (C), 120.2 (CH), 116.2 (C), 115.0 (CH), 114.3 (2 × CH), 91.5 (CH), 56.9 (CH_3), 55.9 (CH), 55.7 (CH_3), 53.6 (C), 47.0 (CH_2), 46.9 (CH_2), 43.9 (CH_2), 42.8 (CH_3), 42.0 (CH), 29.8 (CH_2), 22.5 (CH); HRMS (ESI) Exact mass calculated for $[C_{26}H_{28}NO_5]^+$ $[M+H]^+$: 434.1962, found 434.1963.

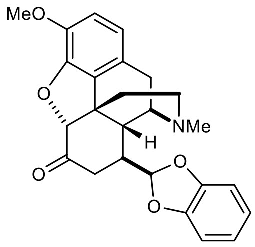

(4*R*,4a*R*,5*S*,7a*R*,12b*S*)-5-(Benzo[*d*][1,3]dioxol-2-yl)-9-methoxy-3-methyl-2,3,4,4a,5,6-hexahydro-1*H*-4,12-methanobenzofuro[3,2-*e*]isoquinolin-7(7a*H*)-one (2ei). Prepared according to a slight modification of the General Procedure using enone **1e** (89 mg, 0.30 mmol) and 1,3-benzodioxole (0.34 mL, 3.00 mmol) as the radical donor, under UV irradiation. Purification by column chromatography (5% 2 M $NH_3[MeOH]/CH_2Cl_2$) gave **2ei** as an off-white solid (43 mg, 34%). R_f = 0.32 (5% 2 M $NH_3[MeOH]/CH_2Cl_2$); m.p. 143-146 °C (Et_2O); $[\alpha]_D^{20.1}$ −201 (*c* 1.00, $CHCl_3$); IR (ATR) 2915, 1729 (C=O), 1608, 1504, 1483, 1437, 1354, 1277, 1234, 1156 cm^{-1}; ^{1}H NMR (400 MHz, $CDCl_3$) δ 6.83-6.75 (4H, m, Ar**H**), 6.73 (1H, d, *J* = 8.2 Hz, Ar**H**), 6.67 (1H, d, *J* = 8.3 Hz, Ar**H**), 6.15 (1H, d, *J* = 3.5 Hz, C**H**O2), 4.70 (1H, s, ArOC**H**), 3.92 (3H, s, ArOC**H**3), 3.56 (1H, dd, *J* = 5.5, 2.9 Hz, C**H**N), 3.10 (1H, d, *J* = 18.6 Hz, ArC**H**aHb), 2.82 (1H, dd, *J* = 11.8, 2.9 Hz, C**H**CHN), 2.65 (1H, dd, *J* = 13.4, 2.6 Hz, O=CC**H**aHb), 2.62-2.56 (1H, m, C**H**aHbN), 2.49-2.36 (5H, m, NC**H**3 and O=CCHa**H**b and ArCHa**H**b), 2.25 (1H, td, *J* = 11.9, 3.3 Hz, CHa**H**bN), 2.14 (1H, td, *J* = 12.0, 4.5 Hz, C**H**aHbCH2N), 2.03-1.93 (1H, m, O=CCH2C**H**), 1.89-1.83 (1H, m, CHa**H**bCH2N); ^{13}C NMR (101 MHz, $CDCl_3$) δ 206.3 (C), 147.3 (C), 147.2 (C), 145.2 (C), 143.0 (C), 127.1 (C), 126.3 (C), 121.9 (CH), 121.9 (CH), 120.2 (CH), 114.9 (CH), 110.5 (CH), 108.7 (CH), 108.6 (CH), 91.6 (CH), 56.9 (CH), 56.9 (CH_3), 47.0 (CH_2), 46.8 (C), 43.5 (CH), 43.2 (CH_3), 40.2 (CH), 39.2 (CH_2), 35.7 (CH_2), 20.0 (CH_2); HRMS (ESI) Exact mass calculated for $[C_{25}H_{26}NO_5]^+$ $[M+H]^+$: 420.1805, found 420.1810.

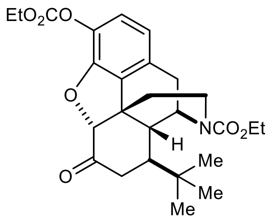

Ethyl (4*R*,4a*R*,5*S*,7a*R*,12b*S*)-5-(*tert*-butyl)-9-[(ethoxycarbonyl)oxy]-7-oxo-1,2,4,4a,5,6,7,7a-octahydro-3*H*-4,12-methanobenzofuro[3,2-*e*]isoquinoline-3-carboxylate (2am). Prepared according to the General Procedure using enone **1a** (124 mg, 0.30 mmol) and pivaldehyde (0.16 mL, 1.50 mmol) as the radical donor, under blue LED irradiation. Purification by column chromatography (50% EtOAc/petrol) gave **2am** (112 mg, 79%) as a white foam, as a 1.5:1 mixture of rotamers. R_f = 0.33 (50% EtOAc/petrol); m.p. 189-192 °C (Et_2O); $[\alpha]_D^{20.1}$ −190 (*c* 1.00, $CHCl_3$); IR (ATR) 2972, 1761 (C=O), 1733 (C=O), 1686 (C=O), 1551, 1484, 1398, 1370, 1317, 1231 cm^{-1}; ^{1}H NMR (400 MHz, $CDCl_3$) δ 6.98 (1H, d, *J* = 8.3 Hz, Ar**H**), 6.74 (1H, d, *J* = 8.2 Hz, Ar**H**), 5.39-5.32 (0.6H, m, C**H**N, rotamer A), 5.25-5.18 (0.4H, m, C**H**N, rotamer B), 4.70 (1H, s, ArOC**H**), 4.41-4.24 (2H, m, OCO_2CH_2), 4.24-3.94 (3H, m, NCO_2CH_2 and CH_aH_bN), 3.13-3.03 (1H, m, $ArCH_aH_b$), 2.87-2.70 (2H, m, $ArCH_aH_b$ and CH_aH_bN), 2.53-2.42 (1H, m, $O{=}CCH_aH_bCH$), 2.38-2.28 (1H, m, C**H**CHN), 2.22-2.11 (1H, m, $O{=}CCH_aH_bCH$), 2.04-1.94 (1H, m, $CH_aH_bCH_2N$), 1.93-1.84 (1H, m, $CH_aH_bCH_2N$), 1.39 (3H, t, *J* = 7.1 Hz, $OCO_2CH_2CH_3$), 1.34-1.24 (4H, m $NCO_2CH_2CH_3$ and $CHC(CH_3)_3$), 1.04 (9H, s, $C(CH_3)_3$, rotamer **A**), 1.03 (9H, s, $C(CH_3)_3$, rotamer **B**); **Rotamer A (Major)** ^{13}C NMR (101 MHz, $CDCl_3$) δ 206.7 (C), 155.5 (C), 153.0 (C), 147.8 (C), 133.2 (C), 131.3 (C), 128.1 (C), 123.0 (CH), 120.3 (CH), 92.1 (CH), 65.2 (CH_2), 61.9 (CH_2), 50.0 (CH), 47.9 (C), 45.8 (CH), 45.0 (CH), 41.8 (CH_2), 37.8 (CH_2), 36.6 (CH_2), 34.1 (C), 29.1 (CH_3 × 3), 29.0 (CH_2), 14.8 (CH_3), 14.3 (CH_3); **Rotamer B (Minor)** ^{13}C NMR (101 MHz, $CDCl_3$) δ 206.5 (C), 155.5 (C), 153.0 (C), 147.8 (C), 133.2 (C), 131.0 (C), 128.1 (C), 122.9 (CH), 120.2 (CH), 92.1 (CH), 65.2 (CH_2), 61.8 (CH_2), 49.9 (CH), 47.8 (C), 45.6 (CH), 45.0 (CH), 41.6 (CH_2), 37.6 (CH_2), 36.4 (CH_2), 34.1 (C), 29.1 (3 × CH_3), 28.9 (CH_2), 14.8 (CH_3), 14.3 (CH_3); HRMS (ESI) Exact mass calculated for $[C_{26}H_{34}NO_7]^+$ $[M+H]^+$: 472.2330, found 472.2331.

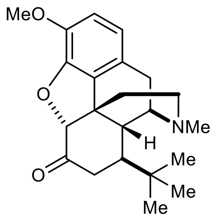

(4*R*,4a*R*,5*S*,7a*R*,12b*S*)-5-(*tert*-Butyl)-9-methoxy-3-methyl-2,3,4,4a,5,6-hexahydro-1*H*-4,12-methanobenzofuro[3,2-*e*]isoquinolin-7(7a*H*)-one (2em). Prepared according to a slight modification of the General Procedure using enone **1e** (54 mg, 0.18 mmol), TBADT (30 mg, 0.009 mmol), MeCN (0.45 mL) and pivaldehyde (0.20 mL, 1.80 mmol) as the radical donor, under UV irradiation. Purification by column chromatography (5% 2 M NH_3[MeOH]/CH_2Cl_2) gave **2em** as an off-white solid (24 mg, 38%). R_f = 0.24 (5% 2 M NH_3[MeOH]/CH_2Cl_2); m.p. 157-161 °C (Et_2O); $[\alpha]_D^{20.1}$ −169 (*c* 1.00, $CHCl_3$); IR (ATR) 2912, 2840, 2800, 1726 (C=O), 1610, 1504, 1438, 1398, 1368, 1334 cm^{-1}; ^{1}H NMR (400 MHz, $CDCl_3$) δ 6.73 (1H, d, *J* = 8.2 Hz, Ar**H**), 6.67 (1H, d, *J* = 8.2 Hz, Ar**H**), 4.67 (1H, s, ArOC**H**), 3.92 (3H, s, OCH_3), 3.60 (1H, dd, *J* = 5.7, 2.7 Hz, C**H**N), 3.01 (1H, d, *J* = 18.5 Hz, $ArCH_aH_b$), 2.60-2.42 (7H, m, NCH_3 and $ArCH_aH_b$ and $O{=}CCH_aH_b$ and CH_aH_bN and C**H**CHN), 2.28-

2.03 (3H, m, O=CCH$_a$**H**$_b$ and CH$_a$**H**$_b$N and C**H**$_a$H$_b$CH$_2$N), 1.84 (1H, ddd, *J* = 12.2, 3.4, 1.7 Hz, CH$_a$**H**$_b$CH$_2$N), 1.31 (1H, td, *J* = 11.3, 3.2 Hz, C**H**C(CH$_3$)$_3$), 0.99 (9H, s, C(C**H**$_3$)$_3$); ^{13}C NMR (101 MHz, CDCl$_3$) δ 208.2 (C), 145.0 (C), 142.8 (C), 128.1 (C), 126.8 (C), 119.8 (CH), 114.5 (CH), 92.0 (CH), 59.1 (CH), 56.9 (CH$_3$), 47.6 (C), 47.0 (CH$_2$), 46.4 (CH), 45.9 (CH), 43.5 (CH$_3$), 42.2 (CH$_2$), 36.9 (CH$_2$), 34.1 (C), 29.3 (3 × CH$_3$), 19.8 (CH$_2$); HRMS (ESI) Exact mass calculated for [C$_{22}$H$_{30}$NO$_3$]$^+$ [M+H]$^+$: 356.2220, found 356.2225.

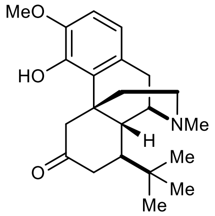

(4b*S*,8*S*,8a*R*,9*R*)-8-(*tert*-Butyl)-4-hydroxy-3-methoxy-11-methyl-8,8a,9,10-tetrahydro-5*H*-9,4b-(epiminoethano)phenanthren-6(7*H*)-one (2gm) Prepared according to a slight modification of the General Procedure using enone **1g** (90 mg, 0.30 mmol) and pivaldehyde (0.33 mL, 3.00 mmol) as the radical donor, under UV irradiation. Purification by column chromatography (7.5% 2 M NH$_3$[MeOH]/CH$_2$Cl$_2$) gave **2gm** as an off-white solid (30 mg, 28%). R$_f$ = 0.39 (10% 2 M NH$_3$[MeOH]/CH$_2$Cl$_2$); m.p. 193-196 °C (Et$_2$O); $[\alpha]_D^{20.1}$ −165 (*c* 1.00, CHCl$_3$); IR (ATR) 2934 (OH), 2837, 1702 (C=O), 1605, 1583, 1483, 1438, 1409, 1367, 1334 cm^{-1}; ^{1}H NMR (400 MHz, CDCl$_3$) δ 6.74-6.62 (2H, m, Ar**H**), 6.06 (1H, br s, O**H**), 4.12 (1H, d, *J* = 18.4 Hz, O=CC**H**$_a$H$_b$C), 3.83 (3H, s, OC**H**$_3$), 3.15-3.07 (1H, m, C**H**N), 3.02-2.96 (2H, m, ArC**H**$_2$), 2.60-2.51 (1H, m, C**H**$_a$H$_b$N), 2.43 (3H, s, NC**H**$_3$), 2.29 (1H, d, *J* = 17.5 Hz, O=CC**H**$_a$H$_b$CH), 2.16-2.07 (2H, m, CH$_a$**H**$_b$N and O=CCH$_a$**H**$_b$C), 2.01 (1H, dd, *J* = 6.5, 3.1 Hz, C**H**CHN), 1.98-1.87 (2H, m, O=CCH$_a$**H**$_b$CH and C**H**$_a$H$_b$CH$_2$N), 1.82 (1H, dt, *J* = 12.7, 7.0 Hz, CH$_a$**H**$_b$CH$_2$N), 1.61 (1H, dd, *J* = 9.3, 6.4 Hz, C**H**C(CH$_3$)$_3$), 0.92 (9H, s, C(C**H**$_3$)$_3$); ^{13}C NMR (101 MHz, CDCl$_3$) δ 214.2 (C), 144.9 (C), 143.4 (C), 131.3 (C), 125.5 (C), 118.8 (CH), 108.8 (CH), 58.9 (CH), 56.1 (CH$_3$), 50.7 (CH$_2$), 47.3 (CH$_2$), 45.4 (CH), 43.4 (CH), 43.2 (CH$_3$), 40.1 (CH$_2$), 38.1 (CH$_2$), 36.8 (C), 35.9 (C), 27.8 (3 × CH$_3$), 23.1 (CH$_2$); HRMS (ESI) Exact mass calculated for [C$_{22}$H$_{32}$NO$_3$]$^+$ [M+H]$^+$: 358.2377, found 358.2368.

Product Derivatization

(4*R*,4a*R*,5*S*,7*S*,7a*R*,12b*S*)-5-(*tert*-Butyl)-3-methyl-2,3,4,4a,5,6,7,7*a*-octahydro-1*H*-4, 12-methanobenzofuro[3,2-*e*]isoquinoline-7,9-diol (3)

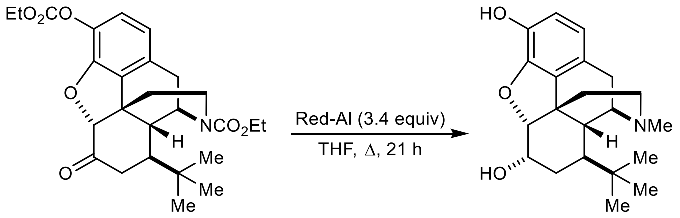

An oven-dried microwave vial fitted with a magnetic stirrer bar was capped with a crimp cap seal and flushed with argon (5 min), then charged with Red-Al (60% wt. in toluene, 0.11 mL, 0.34 mmol). A solution of **2am** (46 mg, 0.10 mmol) in dry THF (0.5 mL) was then added slowly and the resulting mixture was heated under reflux for 21 h. The reaction was cooled to room temperature, quenched carefully with saturated aqueous Rochelle's salt solution (3 mL) and extracted with a 3:1 CH_2Cl_2/MeOH mixture (3 × 5 mL). The combined organic extracts were washed with brine (10 mL), dried (Na_2SO_4), concentrated *in vacuo*, and purified by column chromatography (10% 2 M NH_3[MeOH]/CH_2Cl_2) to give **3** as an off-white foam (25 mg, 71%). R_f = 0.16 (10% 2 M NH_3[MeOH]/CH_2Cl_2); m.p. 190-194 °C (Et_2O); $[\alpha]_D^{20.1}$ −133 (*c* 1.00, $CHCl_3$); IR (ATR) 3286 (OH), 2923, 1503, 1456, 1395, 1365, 1335, 1242, 1175, 1151 cm^{-1}; ^1H NMR (400 MHz, DMSO-d_6) δ 8.76 (1H, br s, ArO**H**), 6.54 (1H, d, *J* = 8.0 Hz, Ar**H**), 6.42 (1H, d, *J* = 8.0 Hz, Ar**H**), 4.45 (1H, d, *J* = 5.3 Hz, CHO**H**), 4.41 (1H, d, *J* = 3.7 Hz, ArOC**H**), 3.68-3.57 (1H, m, C**H**OH), 3.31-3.28 (1H, m, C**H**N), 2.80 (1H, d, *J* = 18.6 Hz, ArC$\mathbf{H}_a$$H_b$), 2.43-2.29 (2H, m, ArC$H_a$$\mathbf{H}_b$ and C$\mathbf{H}_a$$H_b$N), 2.25 (3H, s, NC$\mathbf{H}_3$), 2.09 (1H, td, *J* = 12.0, 3.4 Hz, C$H_a$$\mathbf{H}_b$N), 2.01-1.88 (2H, m, C**H**CHN and C$\mathbf{H}_a$$H_b$$CH_2$N), 1.49-1.41 (1H, m, C$H_a$$\mathbf{H}_b$$CH_2$N), 1.22-1.17 (1H, m, HOCHC$\mathbf{H}_a$$H_b$), 1.01-0.91 (1H, m, HOCHC$H_a$$\mathbf{H}_b$), 0.89-0.84 (10H, m, C**H**C$(CH_3)_3$ and C(C$\mathbf{H}_3$)$_3$); ^{13}C NMR (101 MHz, DMSO-d_6) δ 145.9 (C), 137.7 (C), 130.7 (C), 125.7 (C), 118.0 (CH), 116.7 (CH), 90.7 (CH), 66.1 (CH), 59.9 (CH), 54.9 (C), 45.5 (CH_2), 43.4 (CH_3), 40.4 (CH), 38.6 (CH), 38.3 (CH_2), 35.4 (C), 28.3 (3 × CH_3), 27.1 (CH_2), 19.4 (CH_2); HRMS (ESI) Exact mass calculated for $[C_{21}H_{30}NO_3]^+$ $[M+H]^+$: 344.2220, found 344.2225.

NMR Spectra of New Compounds

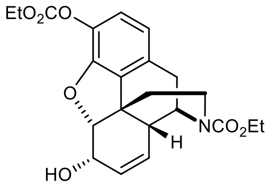

S2
(1.3:1 mixture of rotamers)

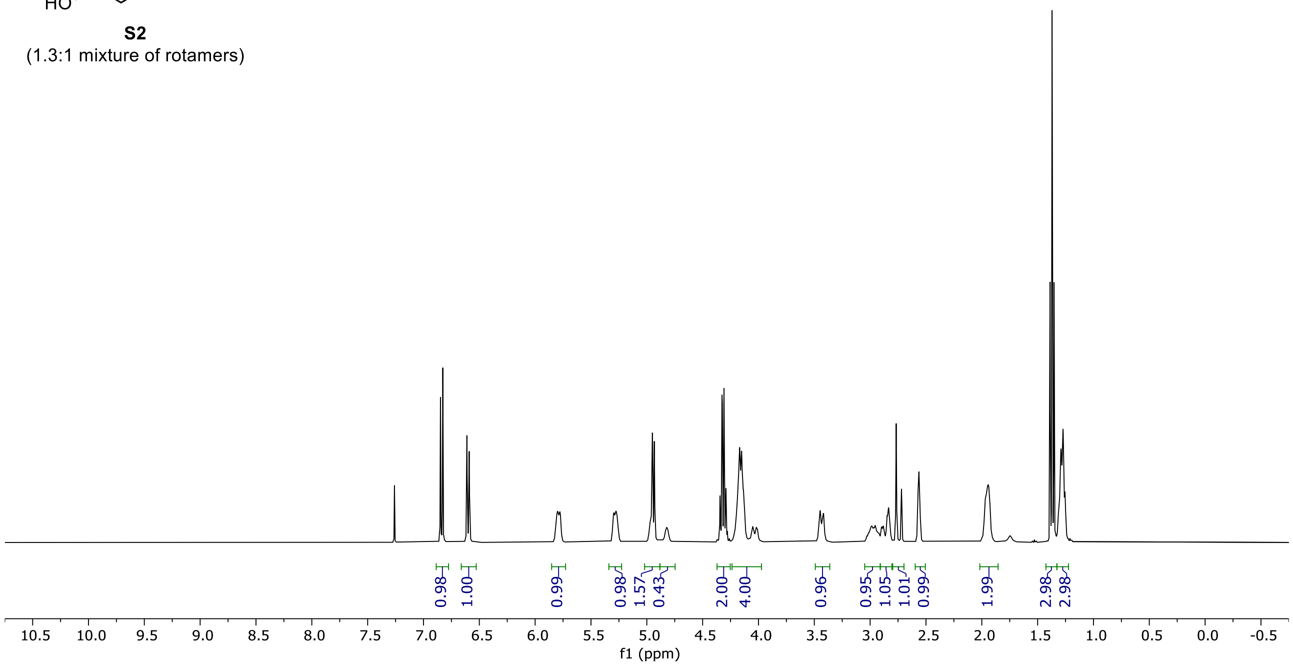

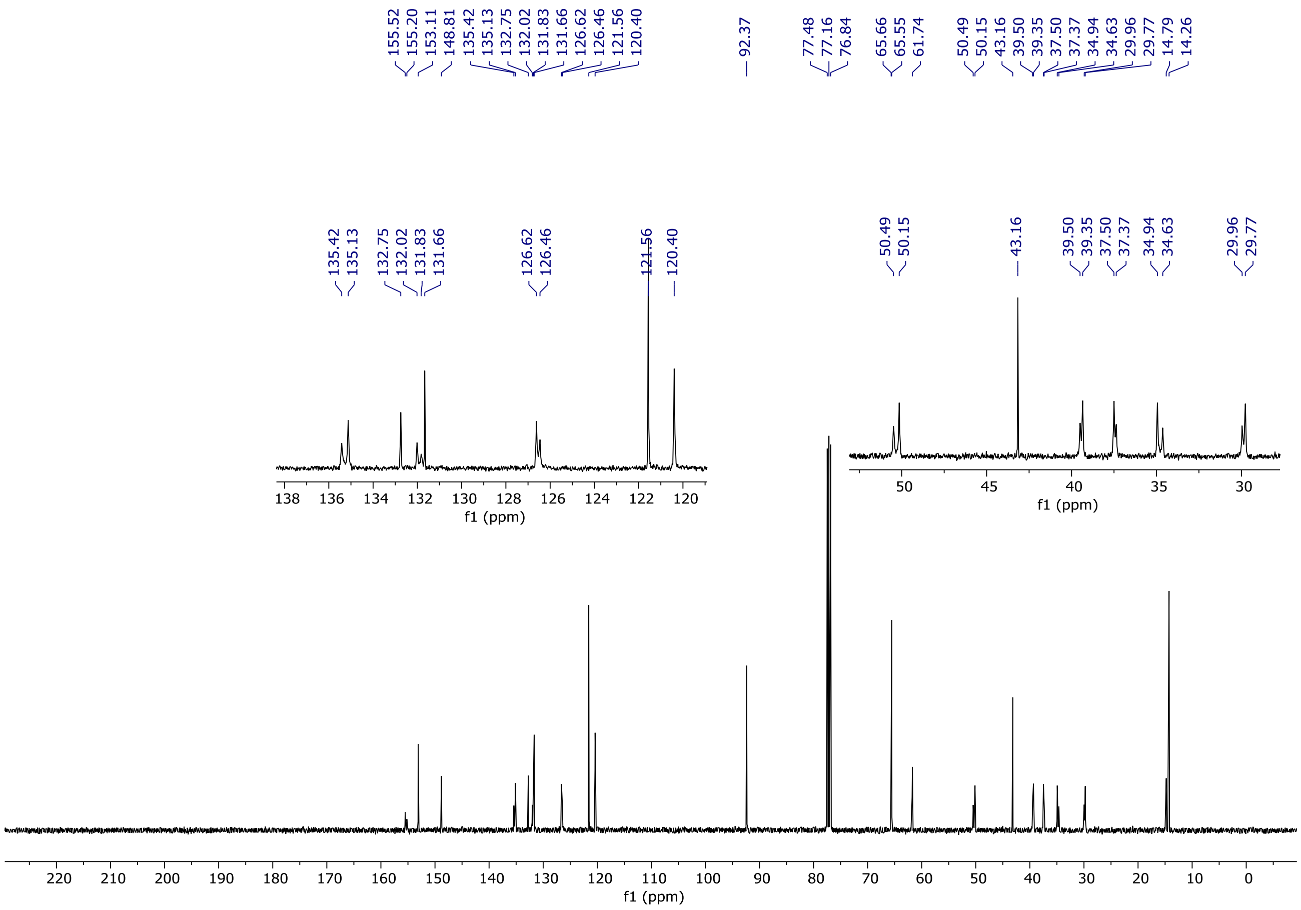

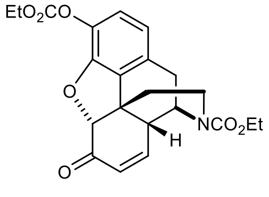

1a
(1.9:1 mixture of rotamers)

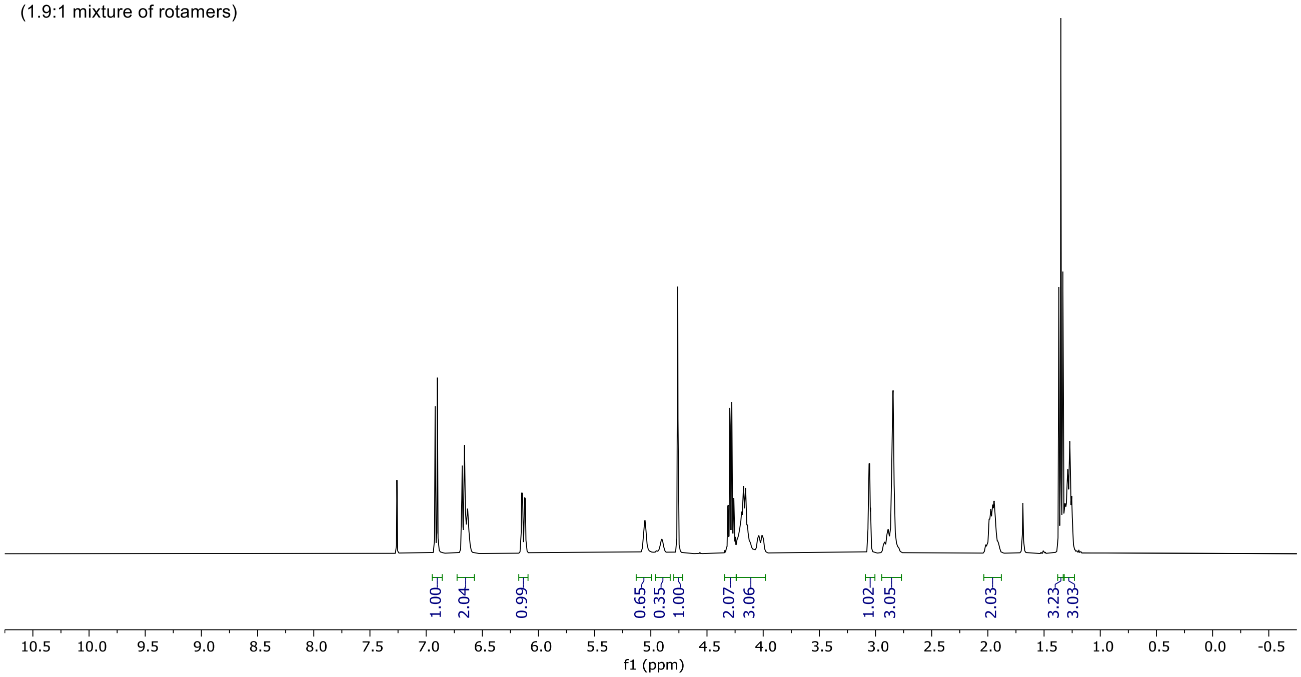

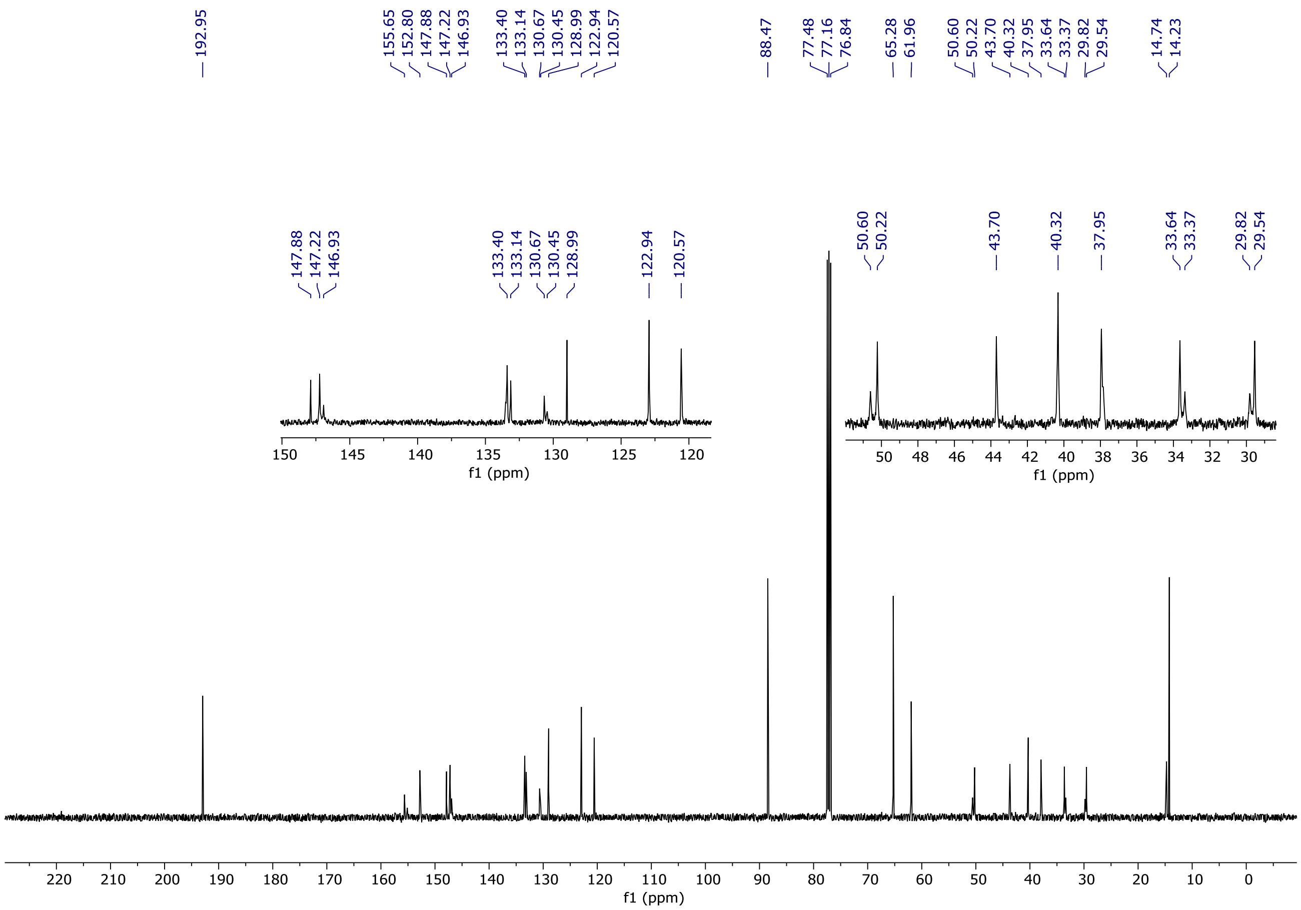

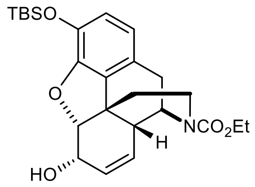

S4
(1.5:1 mixture of rotamers)

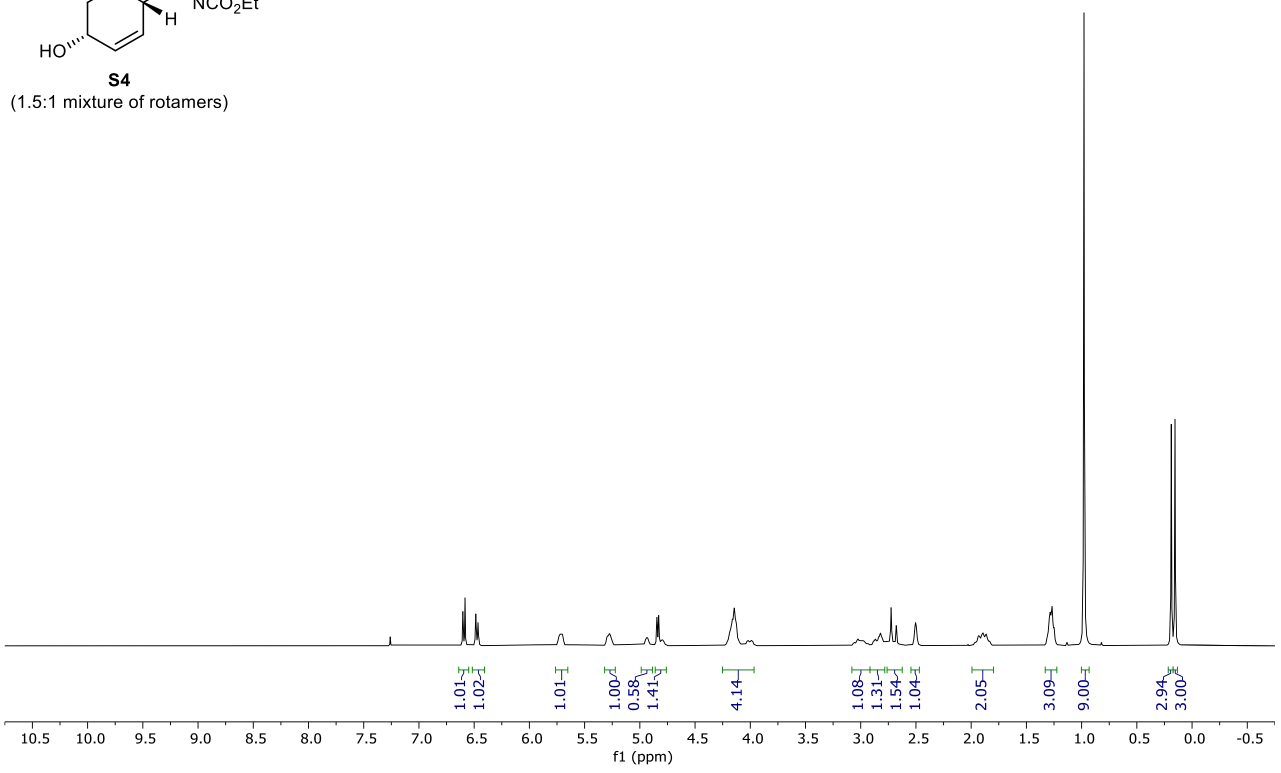

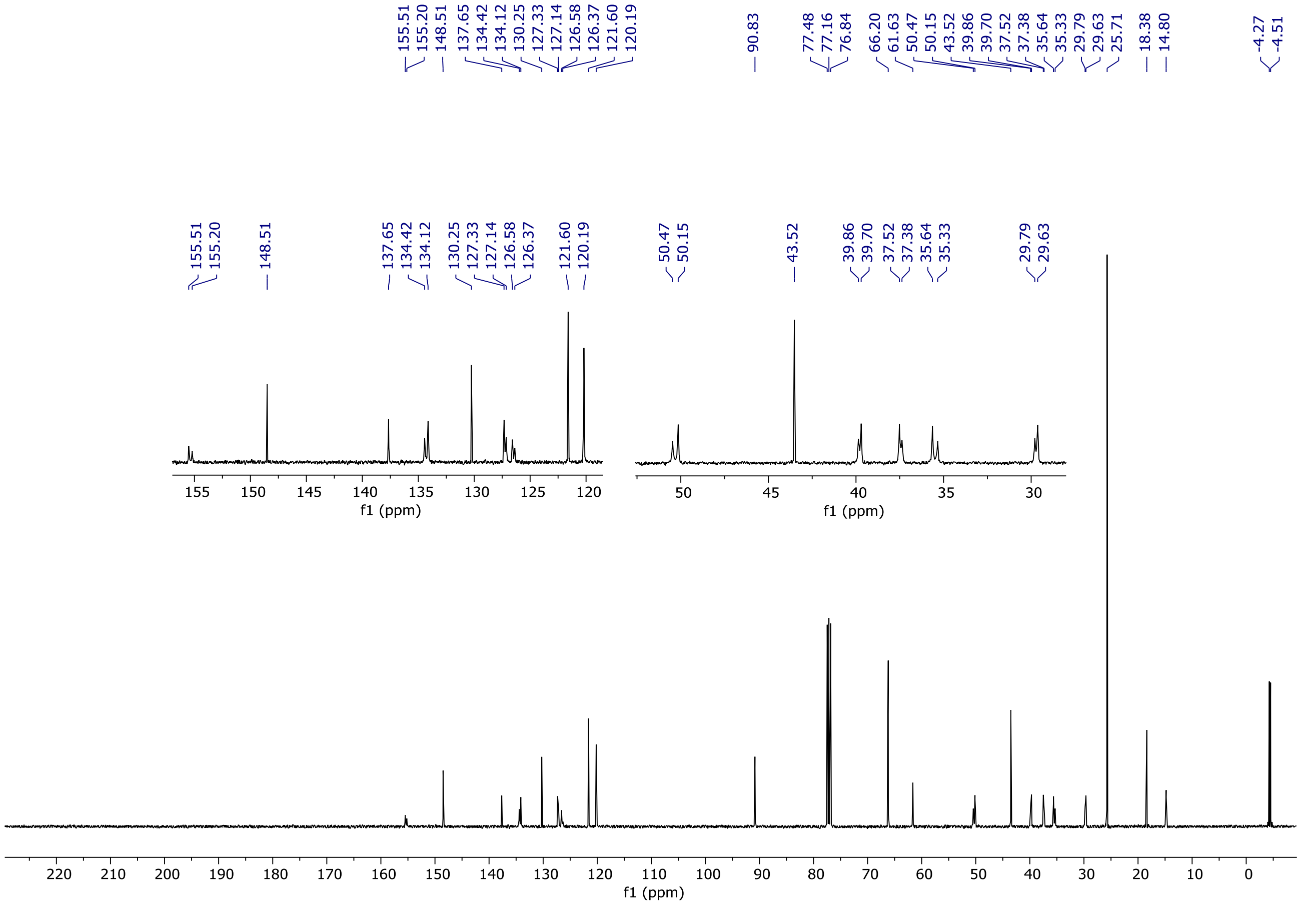

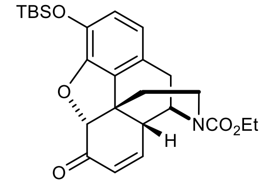

1b
(1.5:1 mixture of rotamers)

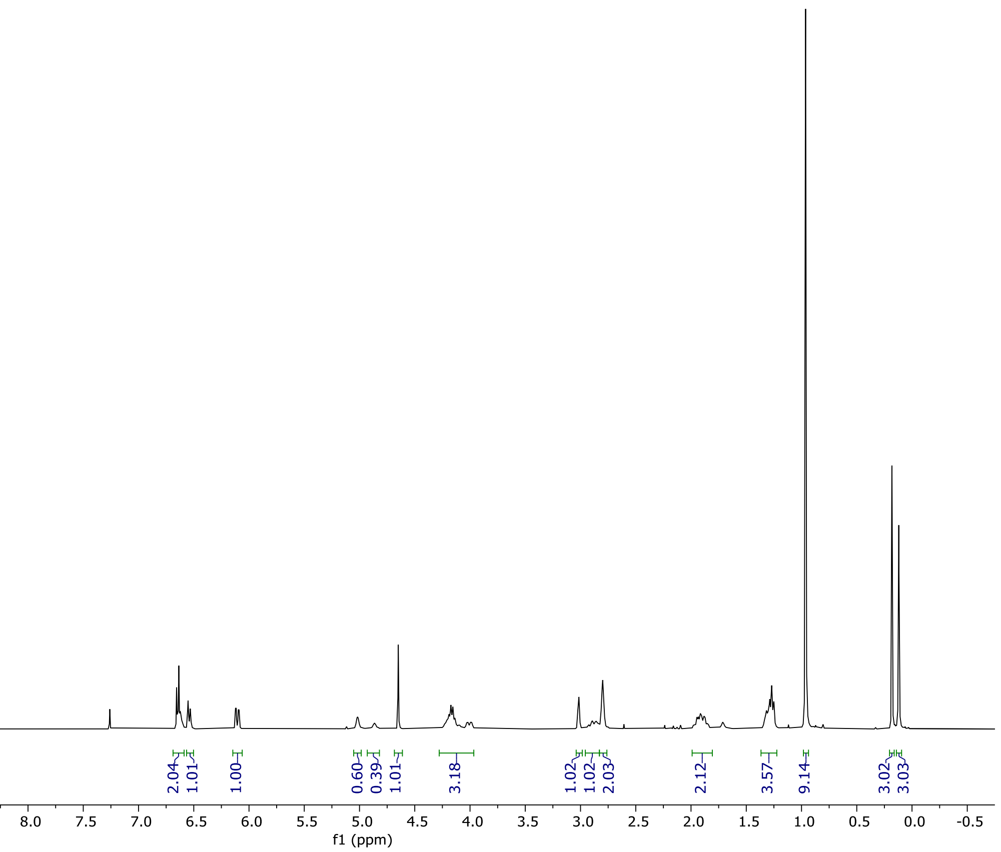

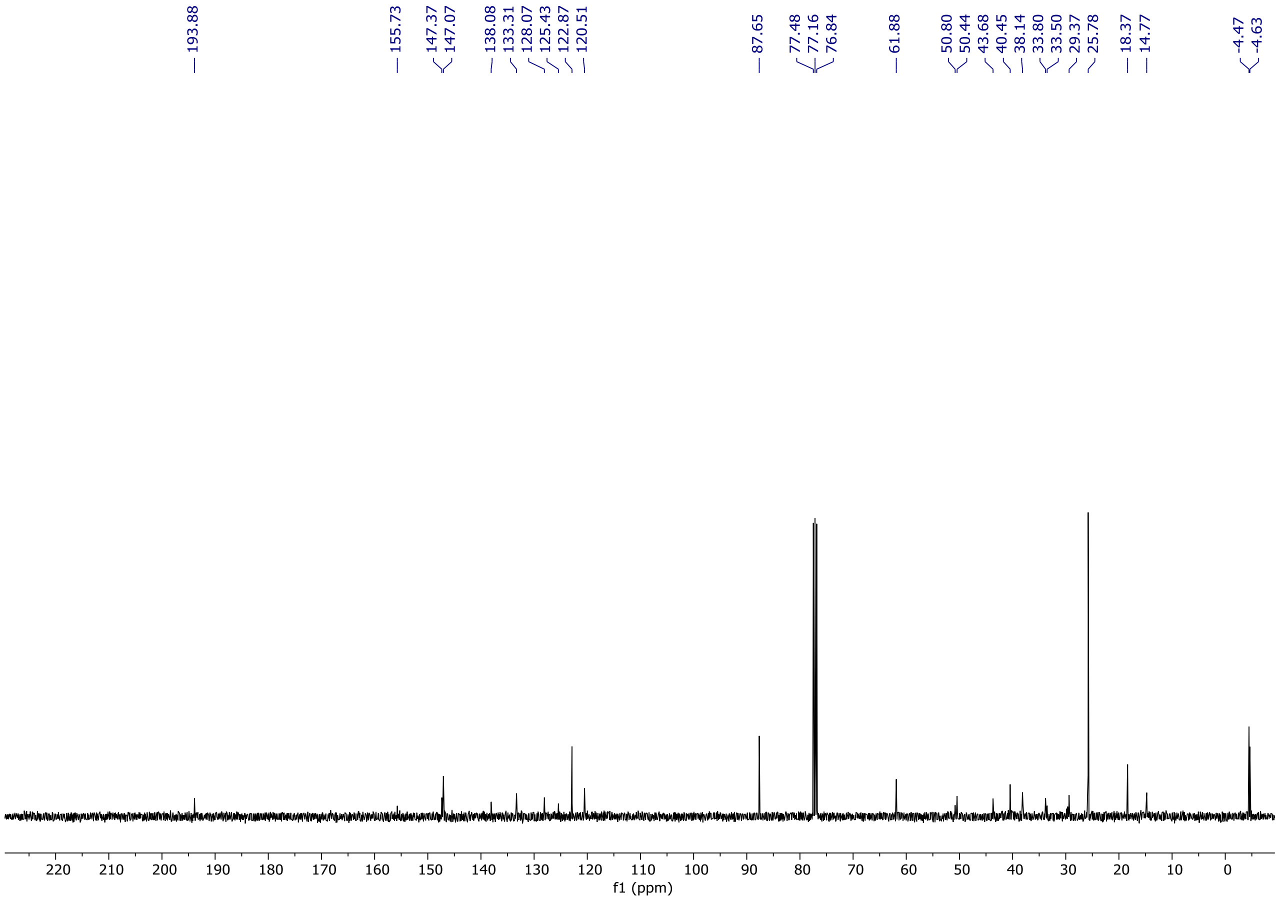

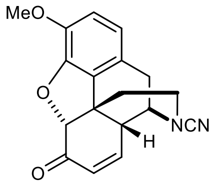
MeO
O
H
NCN
O

1d

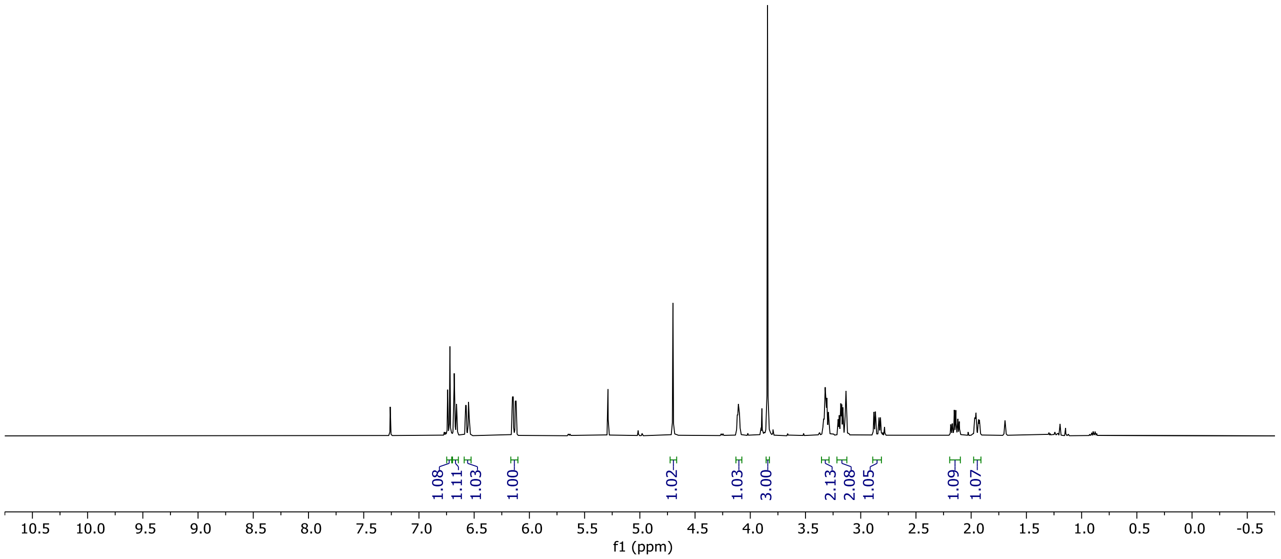
1.08
1.11
1.03
1.00
1.02
1.03
3.00
2.13
2.08
1.05
1.09
1.07
10.5 10.0 9.5 9.0 8.5 8.0 7.5 7.0 6.5 6.0 5.5 5.0 4.5 4.0 3.5 3.0 2.5 2.0 1.5 1.0 0.5 0.0 -0.5
f1 (ppm)

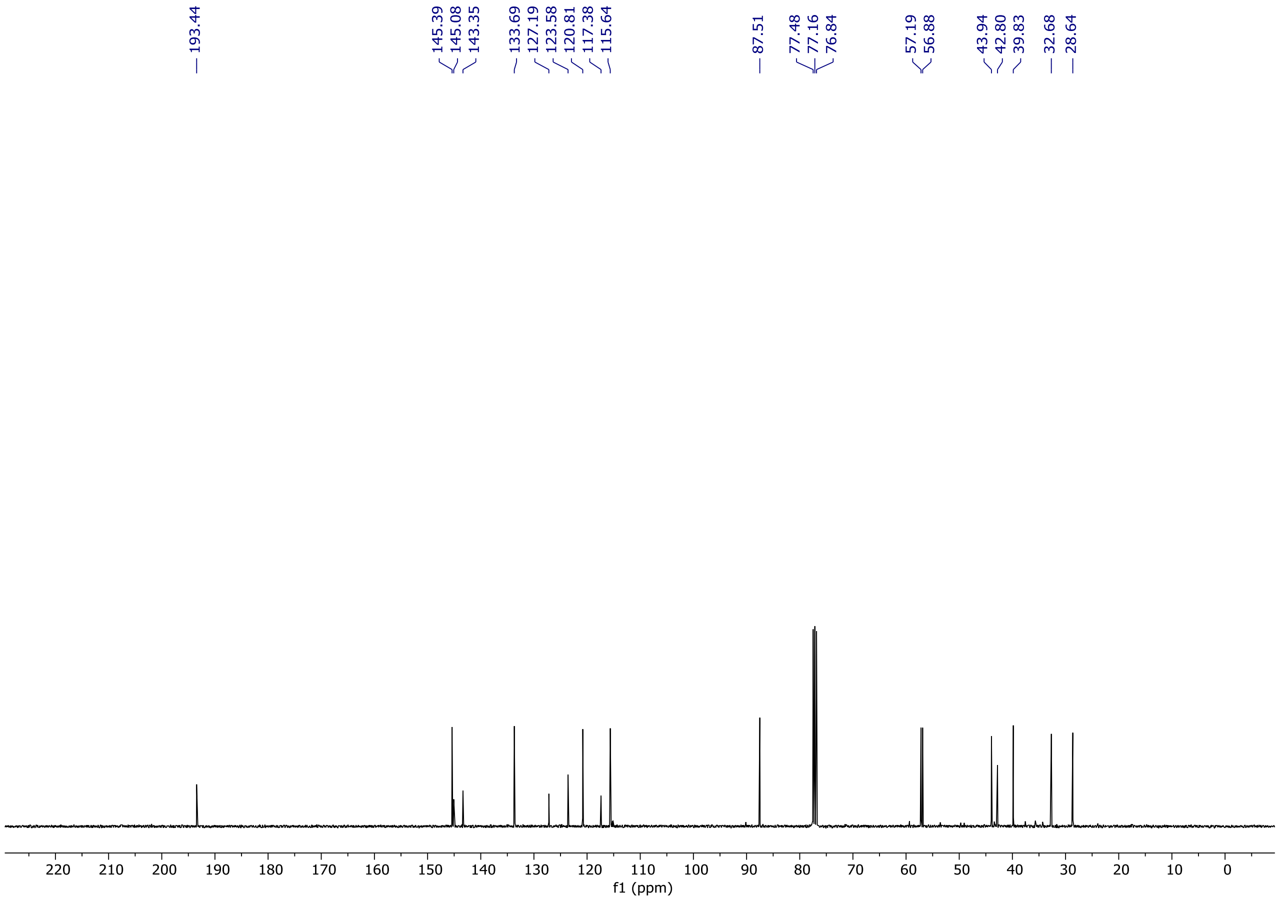
193.44
145.39
145.08
143.35
133.69
127.19
123.58
120.81
117.38
115.64
87.51
77.48
77.16
76.84
57.19
56.88
43.94
42.80
39.83
32.68
28.64
220 210 200 190 180 170 160 150 140 130 120 110 100 90 80 70 60 50 40 30 20 10 0
f1 (ppm)

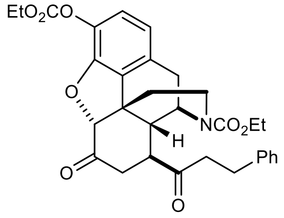

2aa
(*ca.* 1:1 mixture of rotamers)

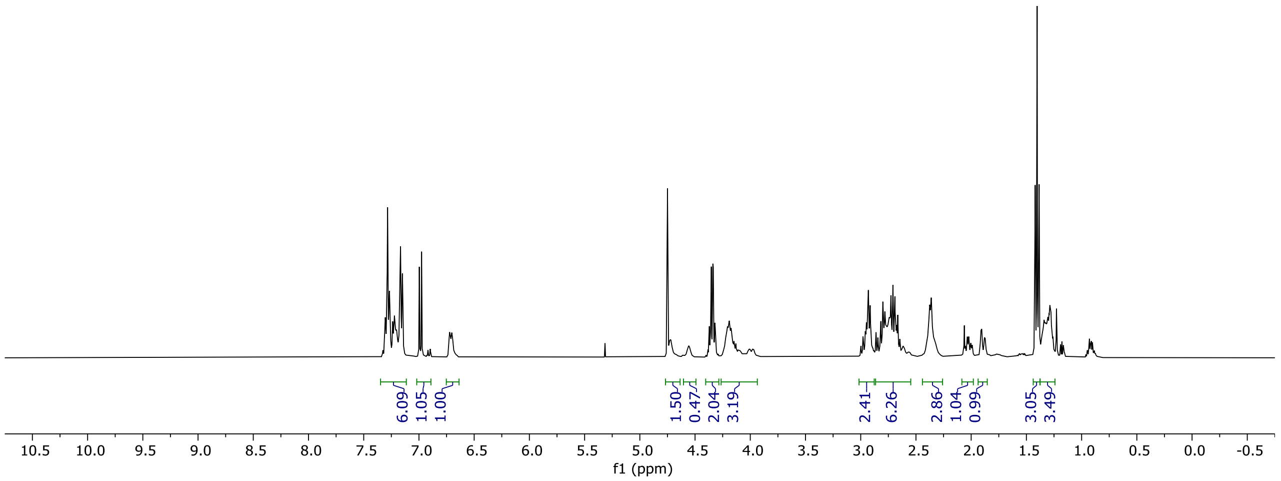

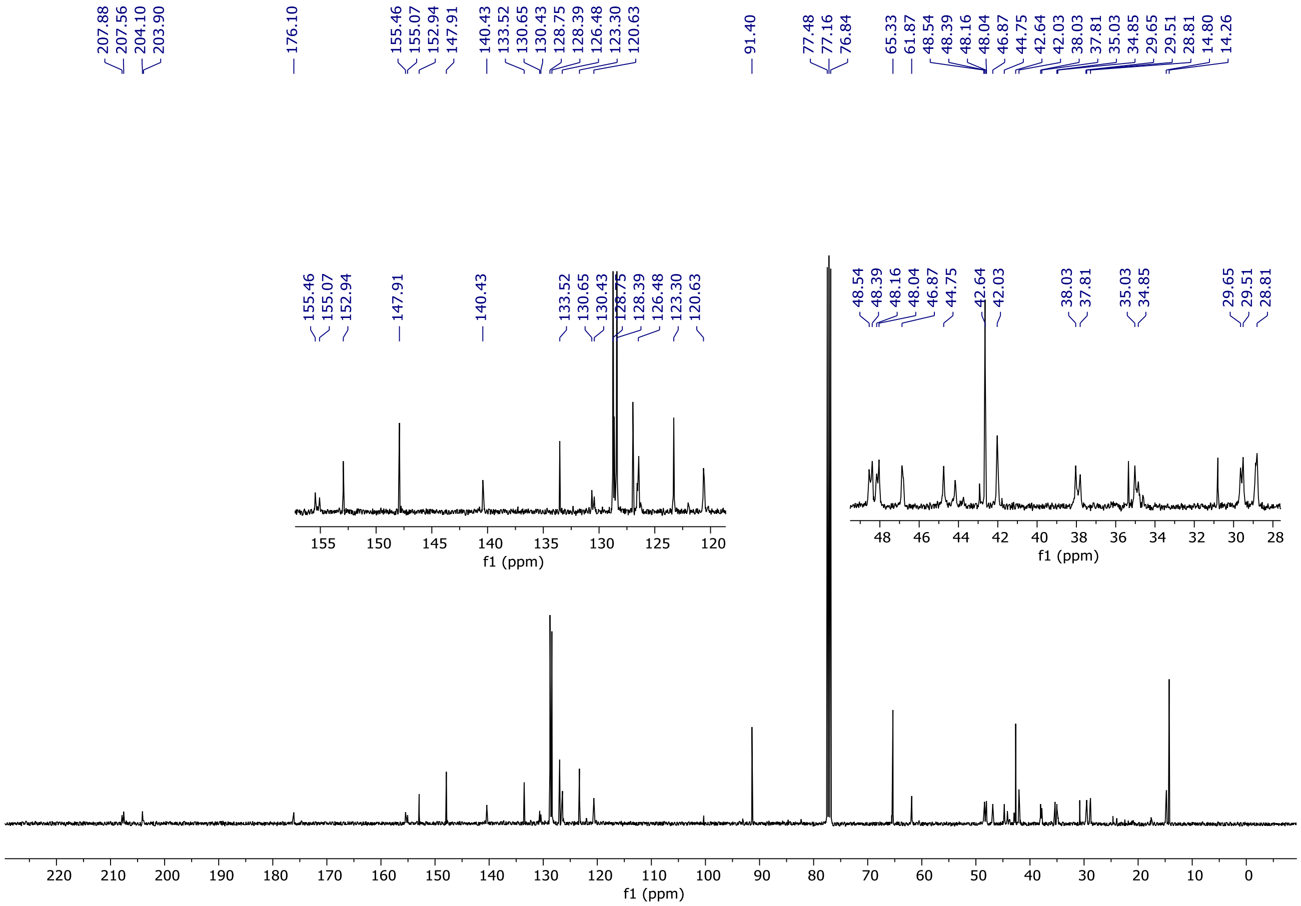

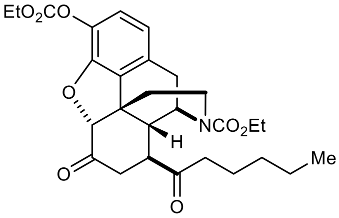

2ab
(*ca.* 1:1 mixture of rotamers)

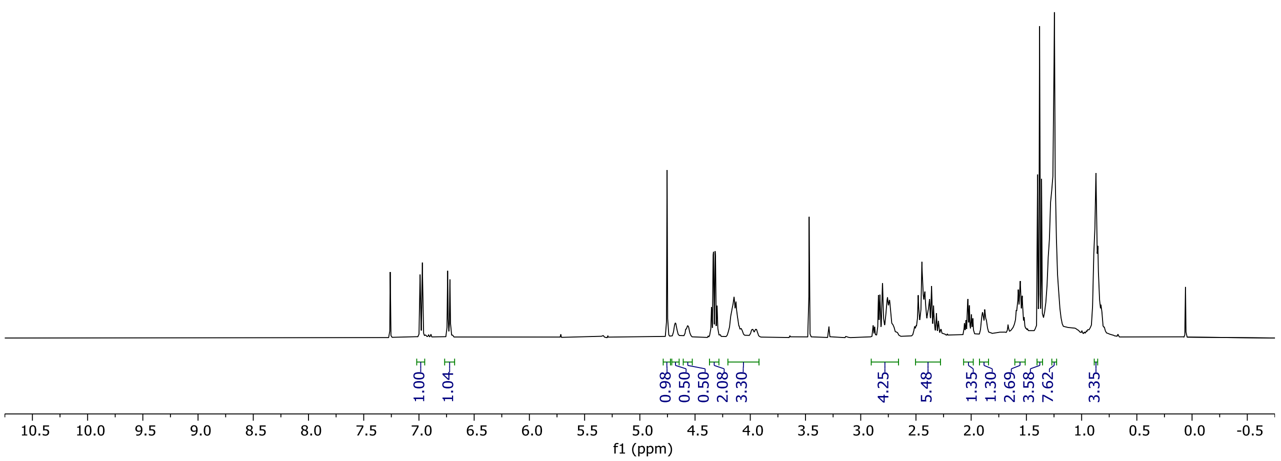

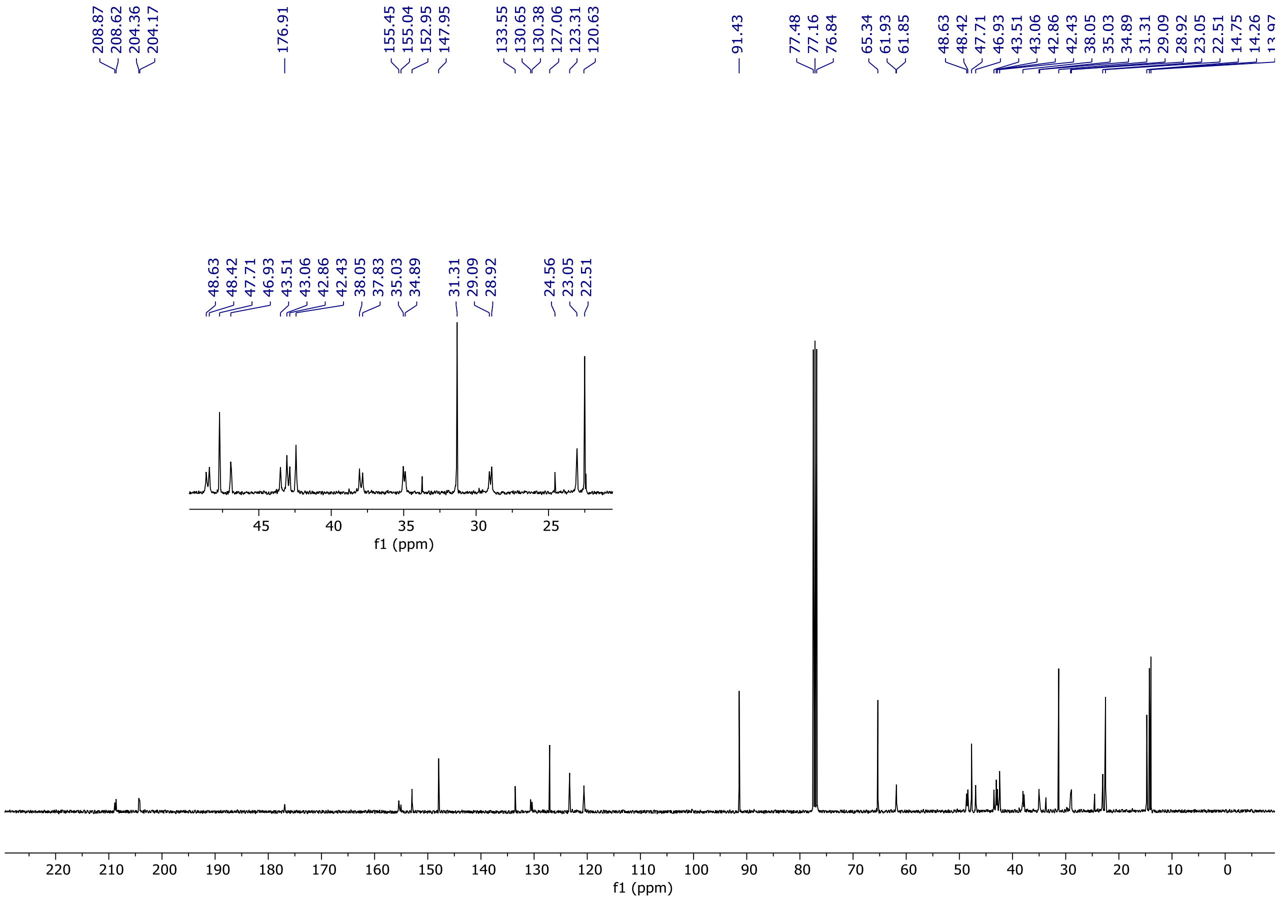

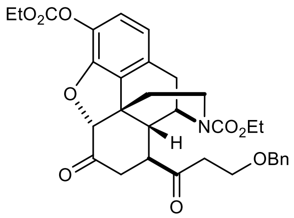

2ac
(*ca.* 1:1 mixture of rotamers)

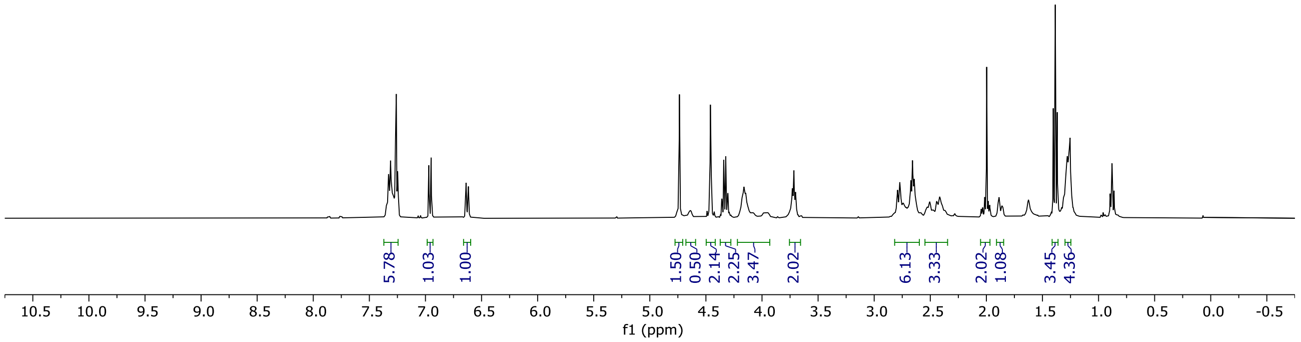

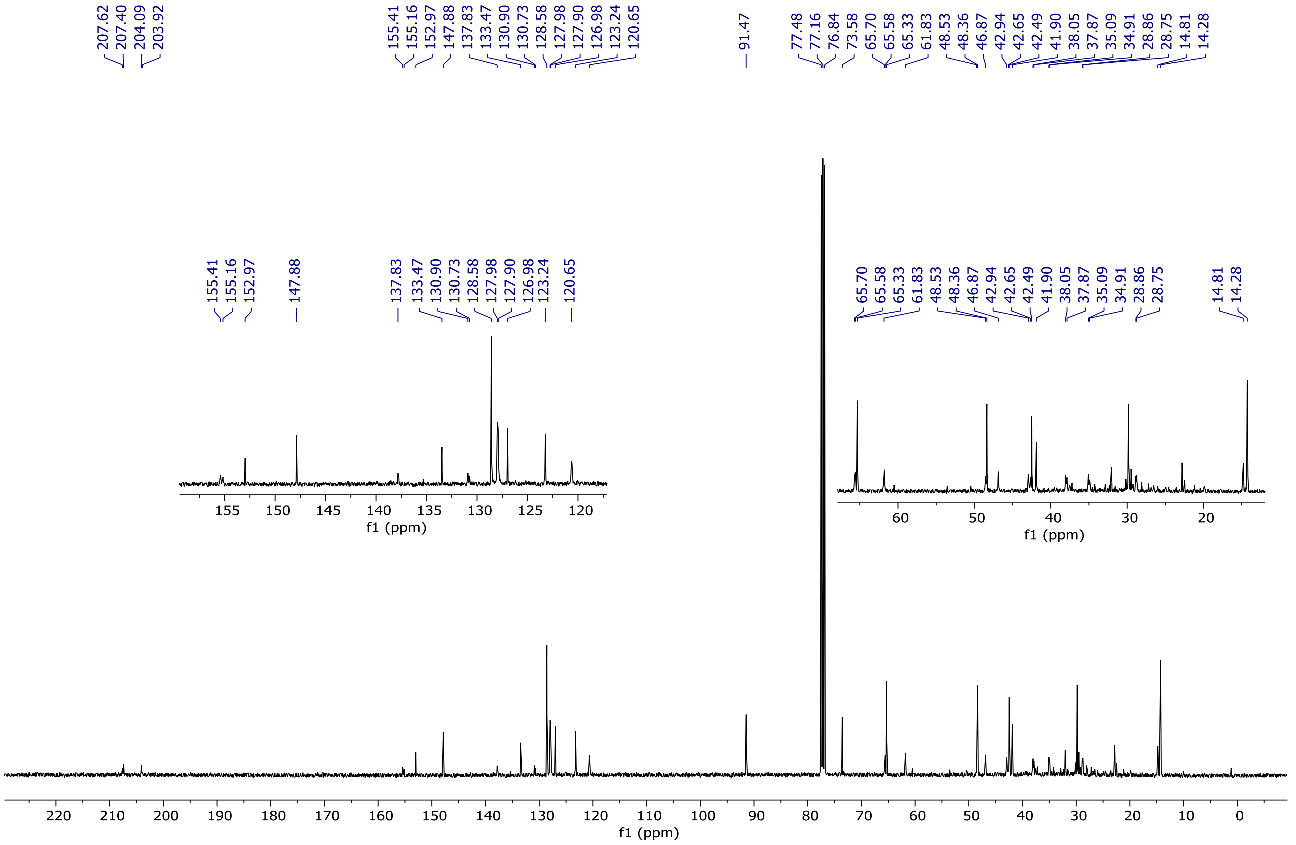

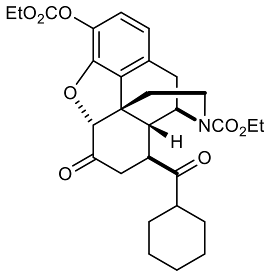

2ad
(*ca.* 1:1 mixture of rotamers)

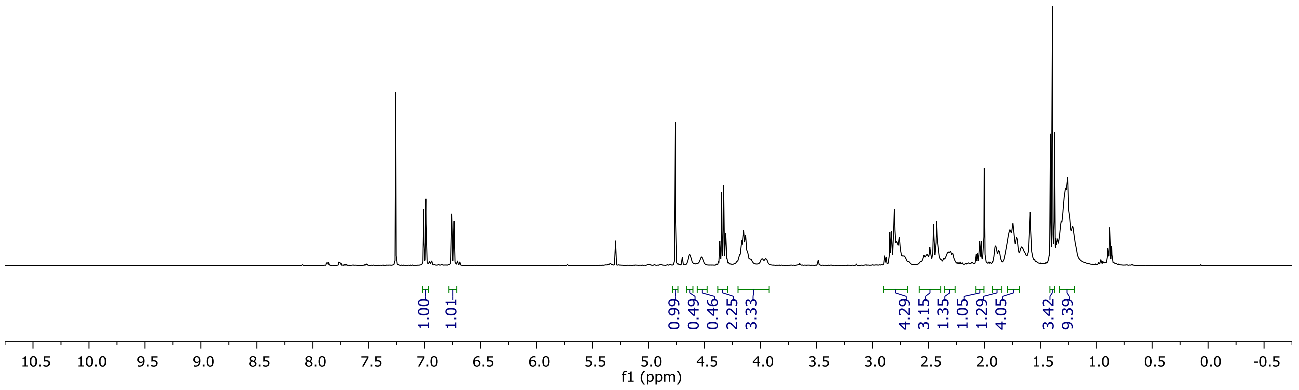

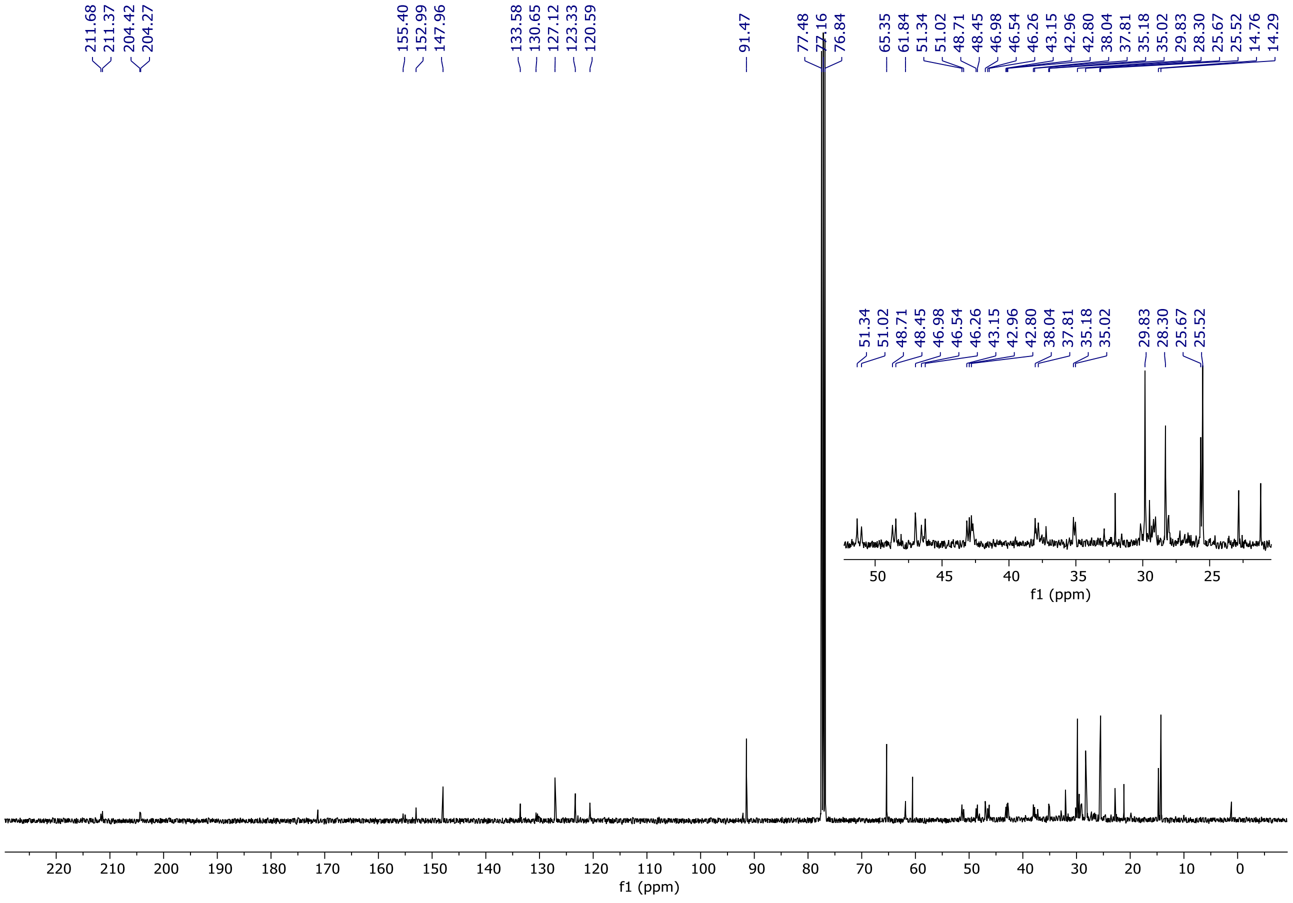

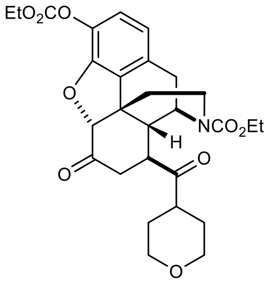

2ae
(1.5:1 mixture of rotamers)

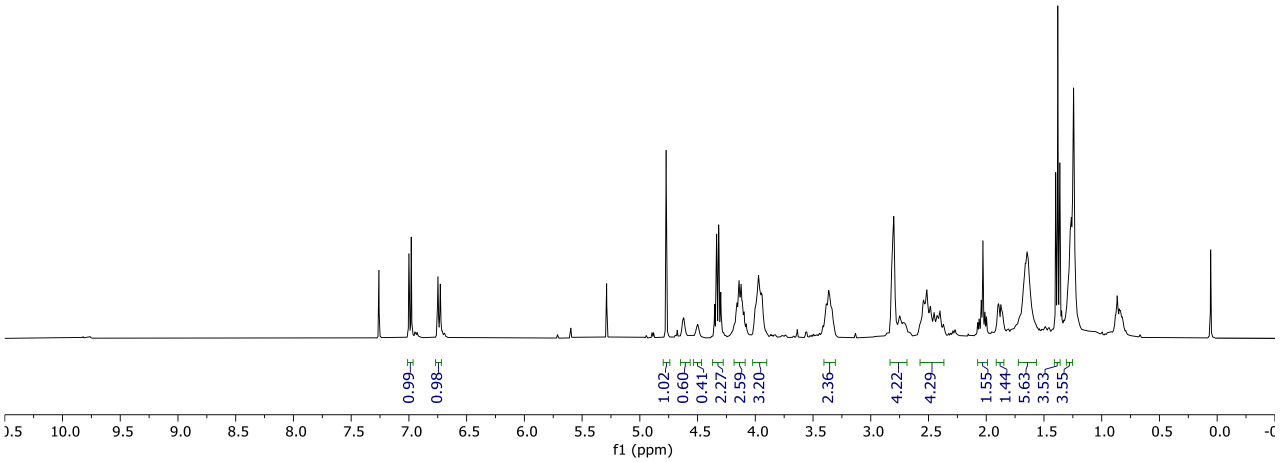

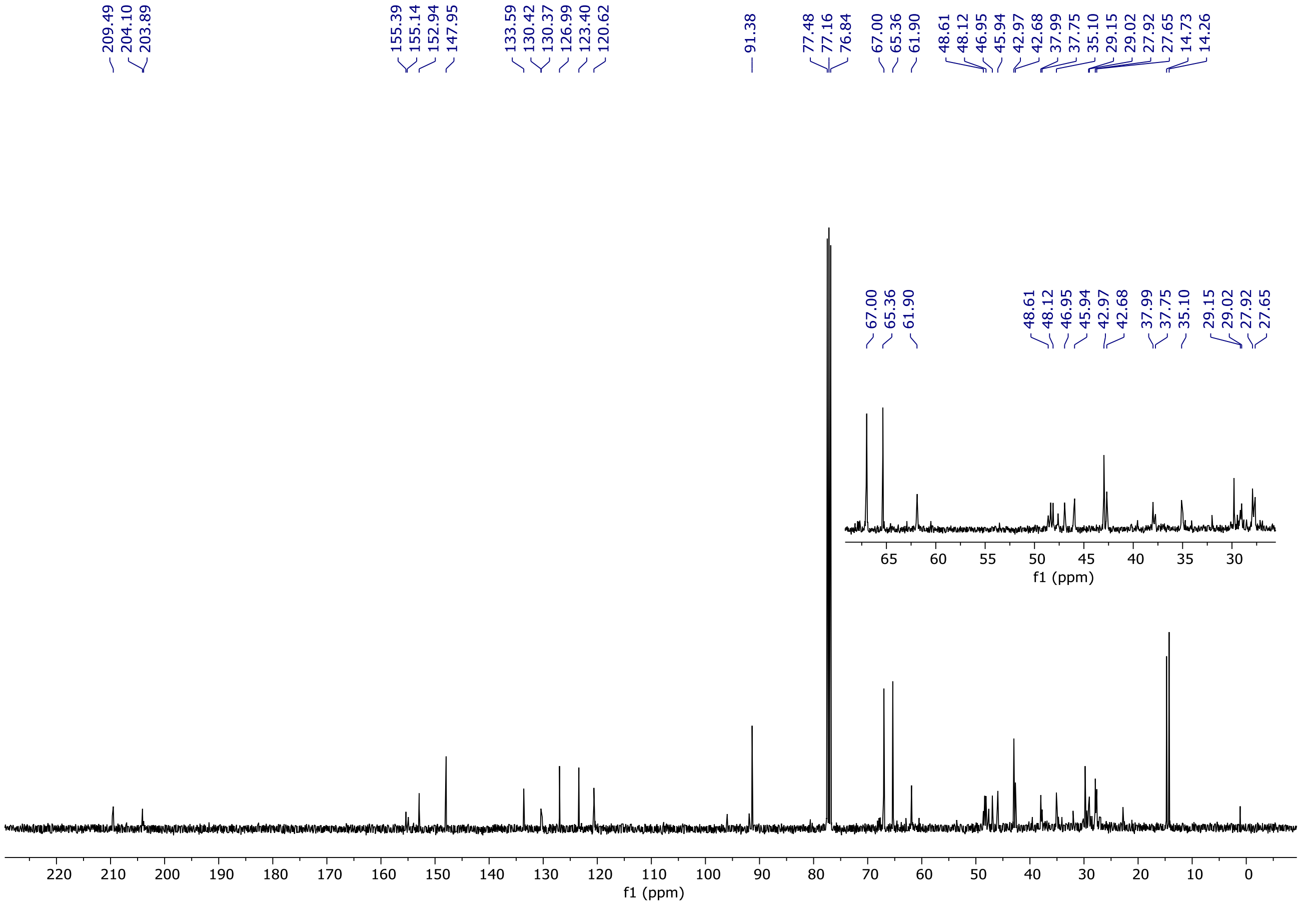

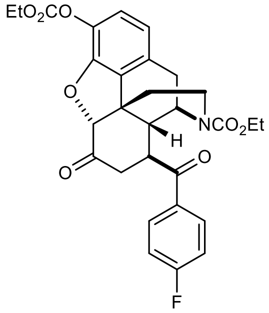

2af
(1.5:1 mixture of rotamers)

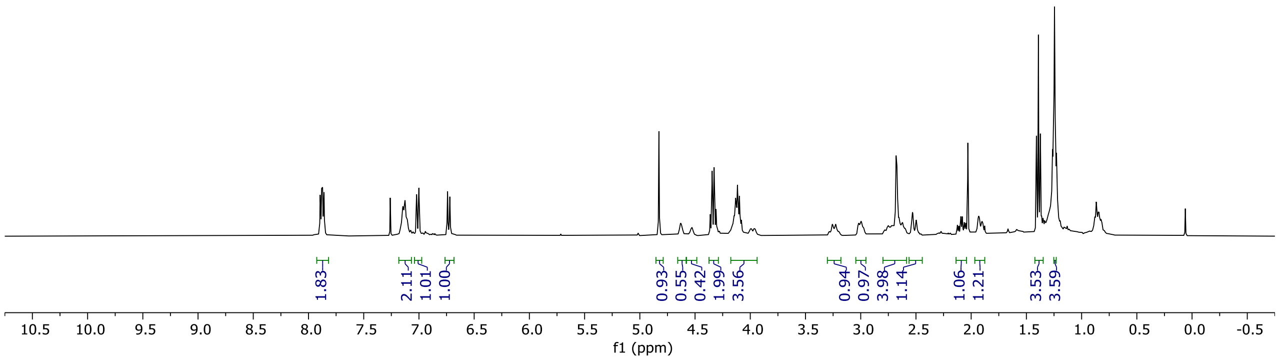

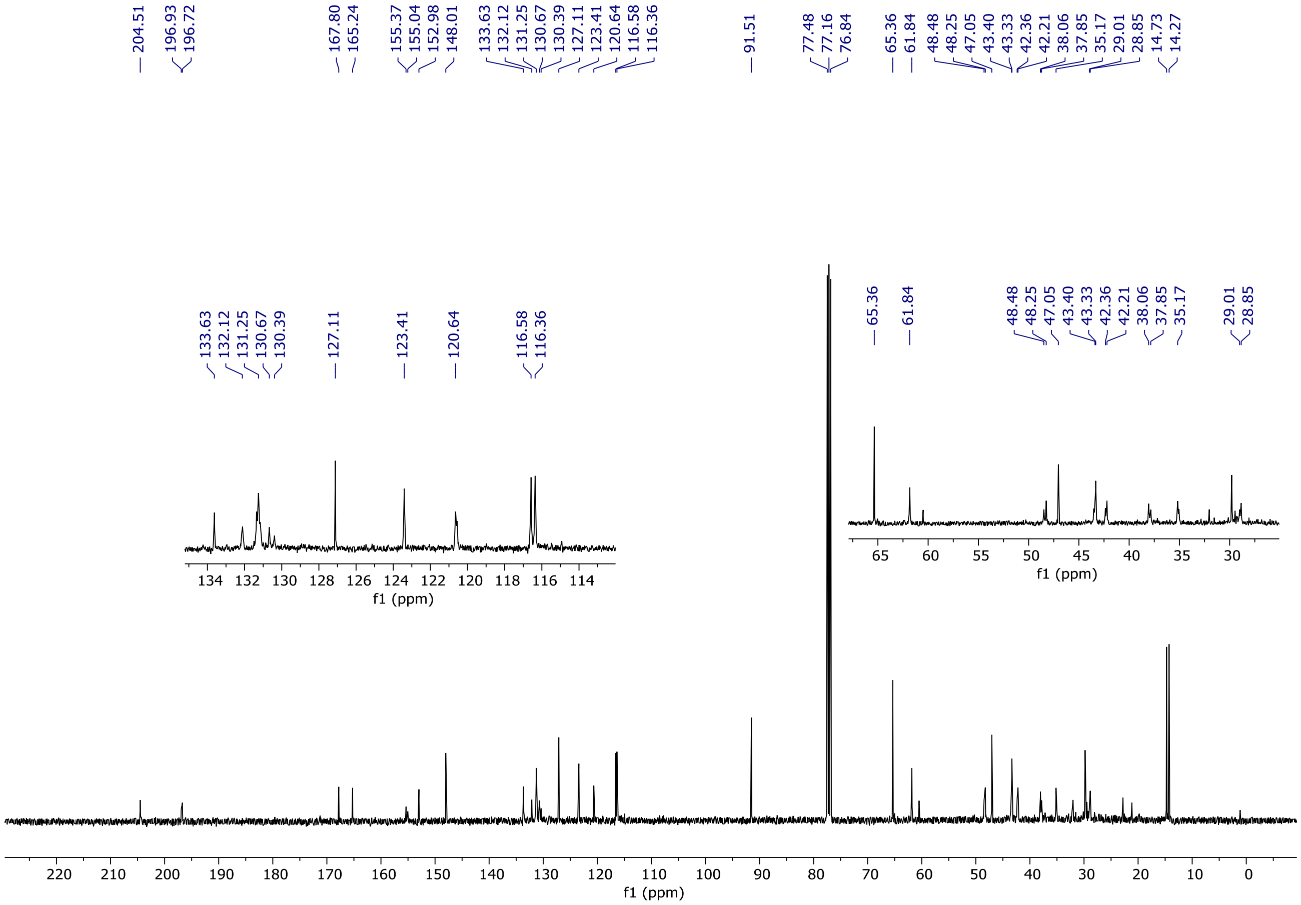

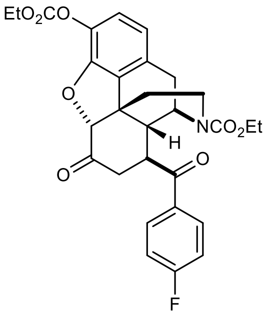

2af
(1.5:1 mixture of rotamers)

^{19}F NMR spectrum

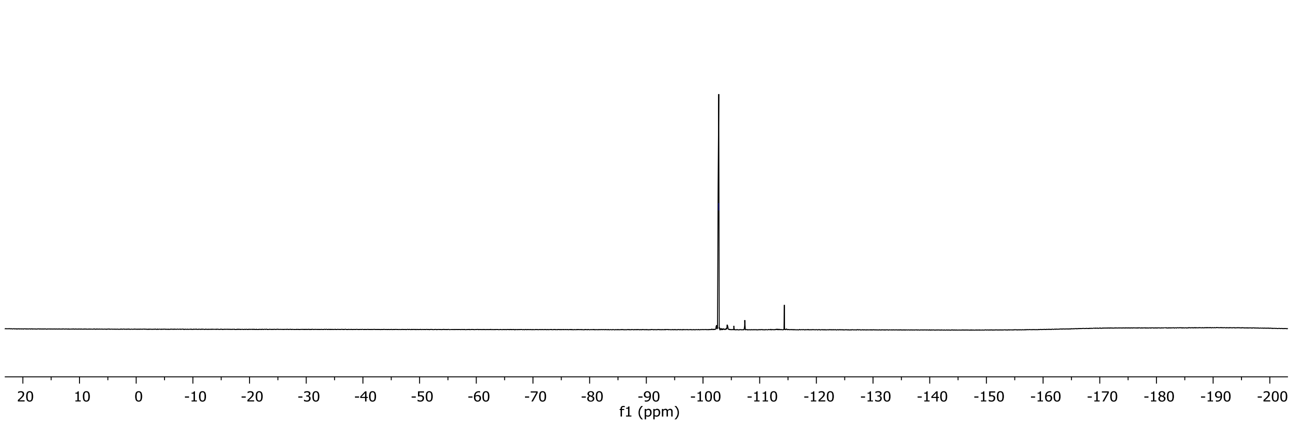

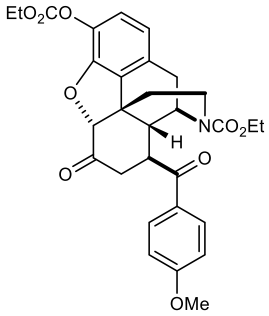

2ag
(1.5:1 mixture of rotamers)

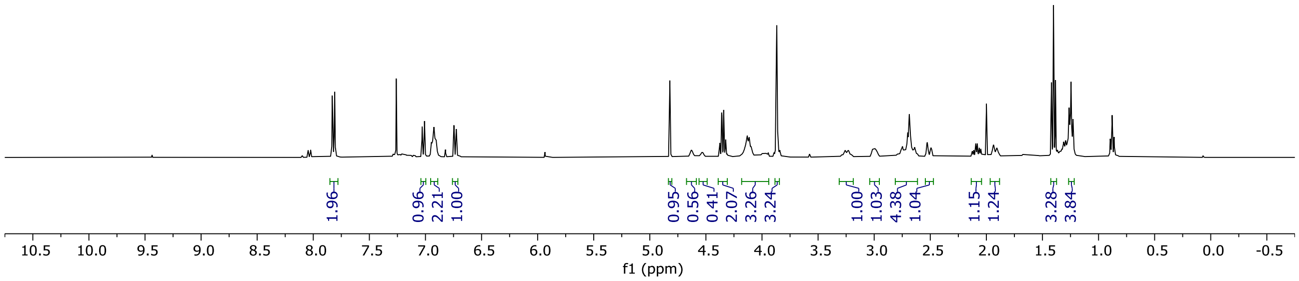

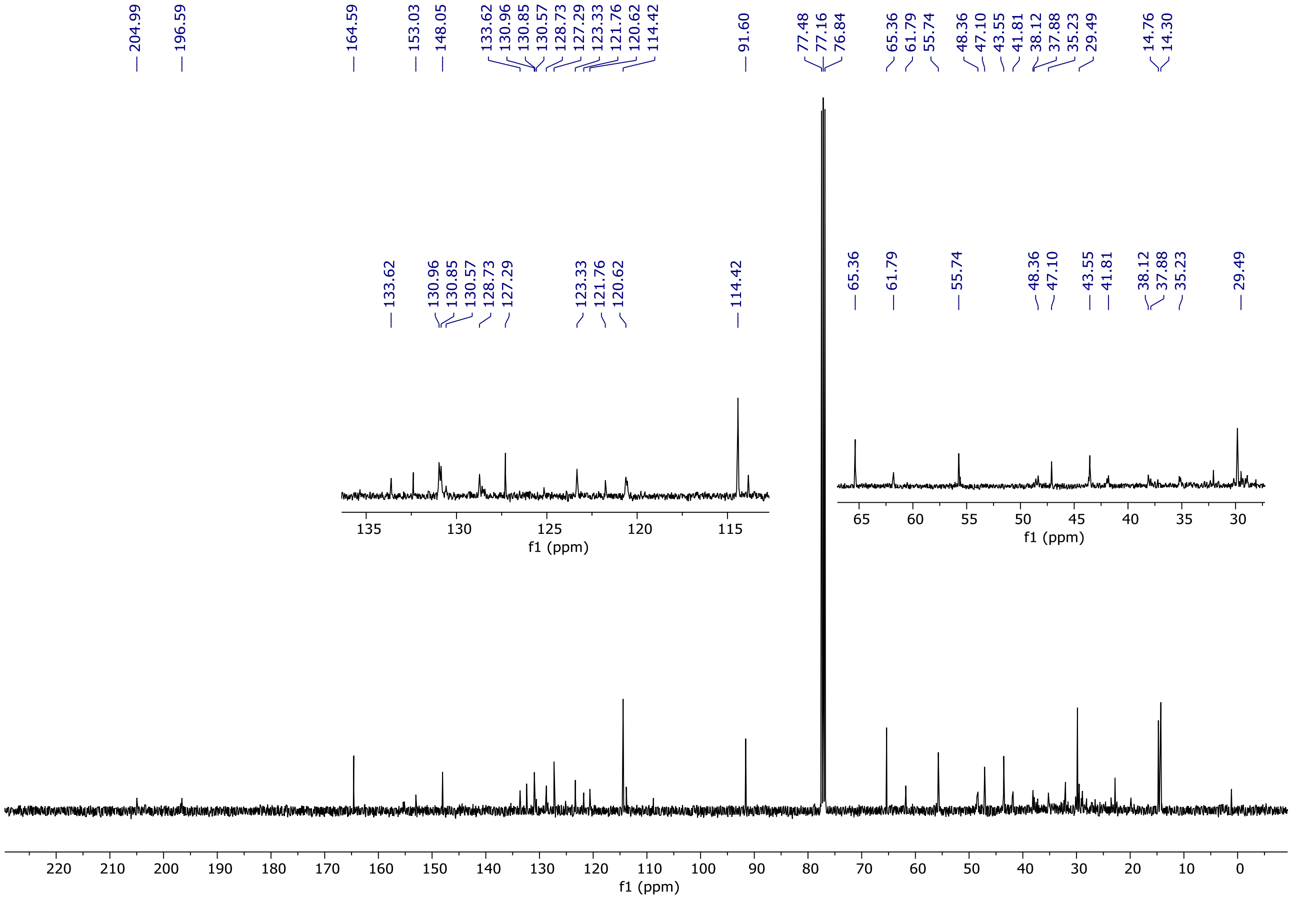

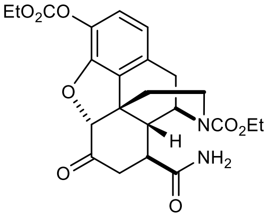

2ah
(2.3:1 mixture of rotamers)

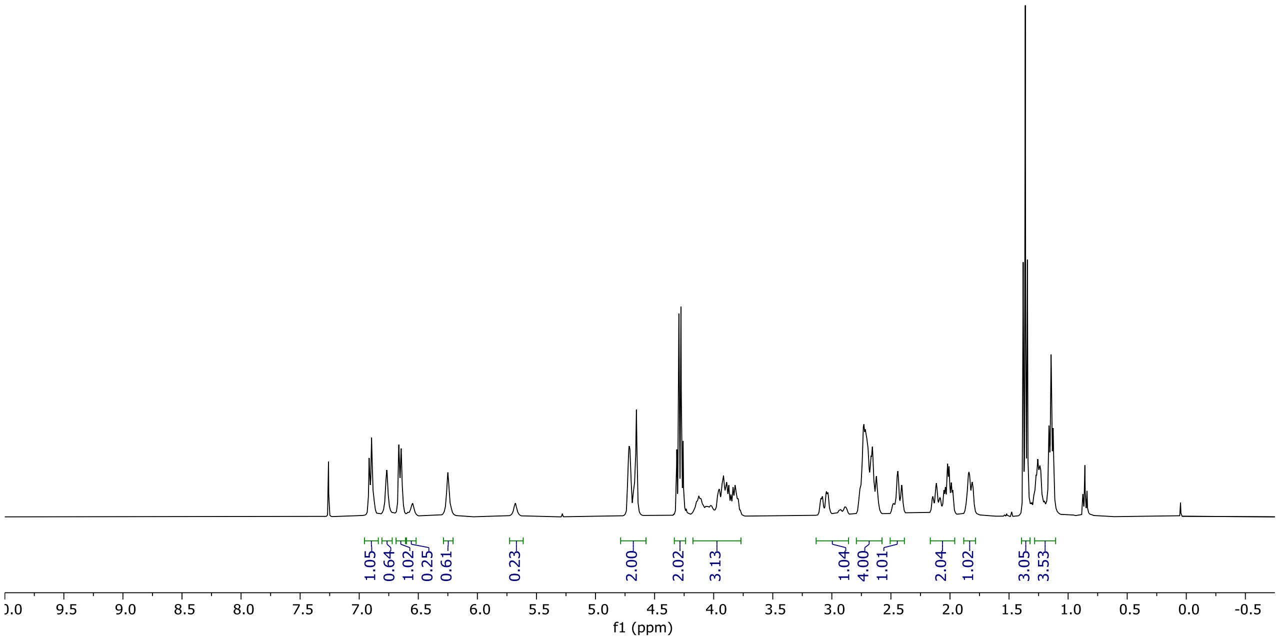

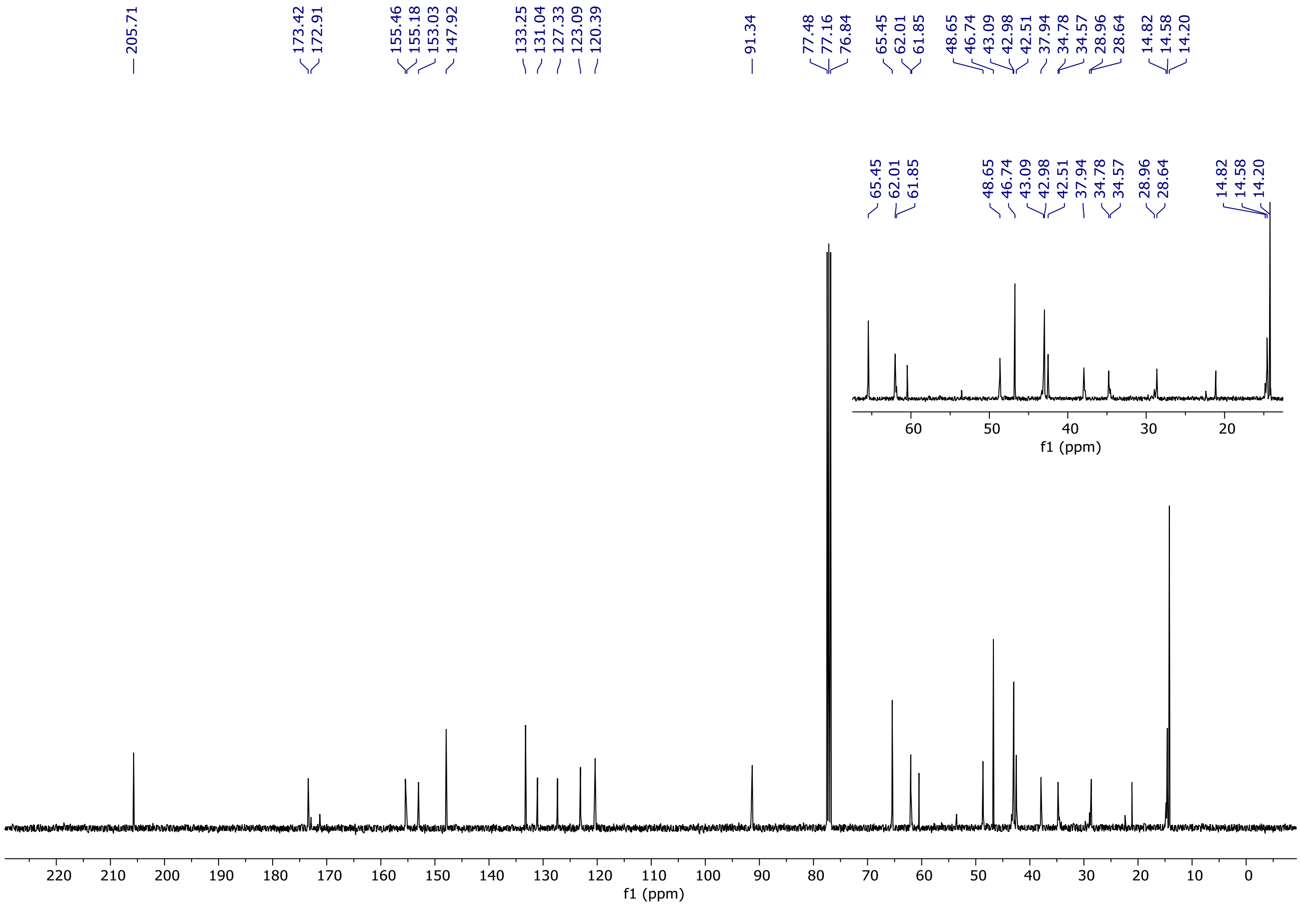

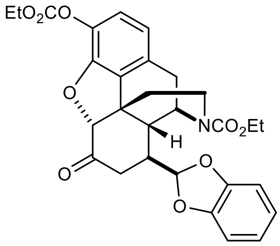

2ai
(*ca.* 1:1 mixture of rotamers)

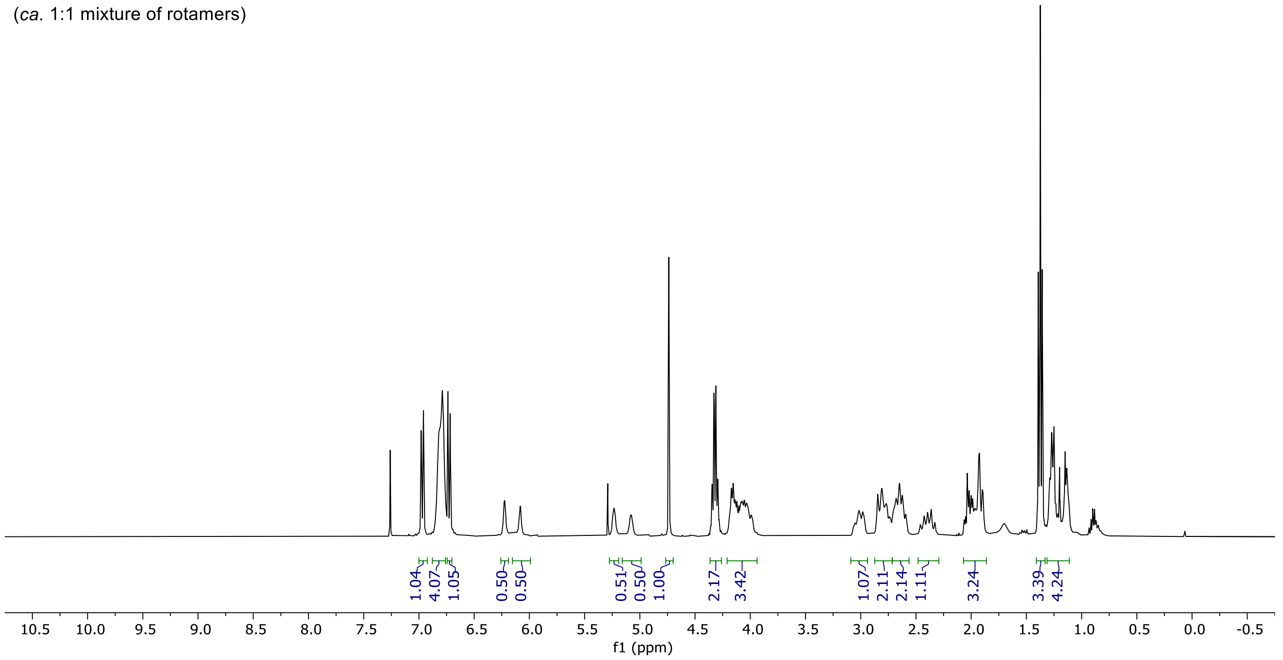

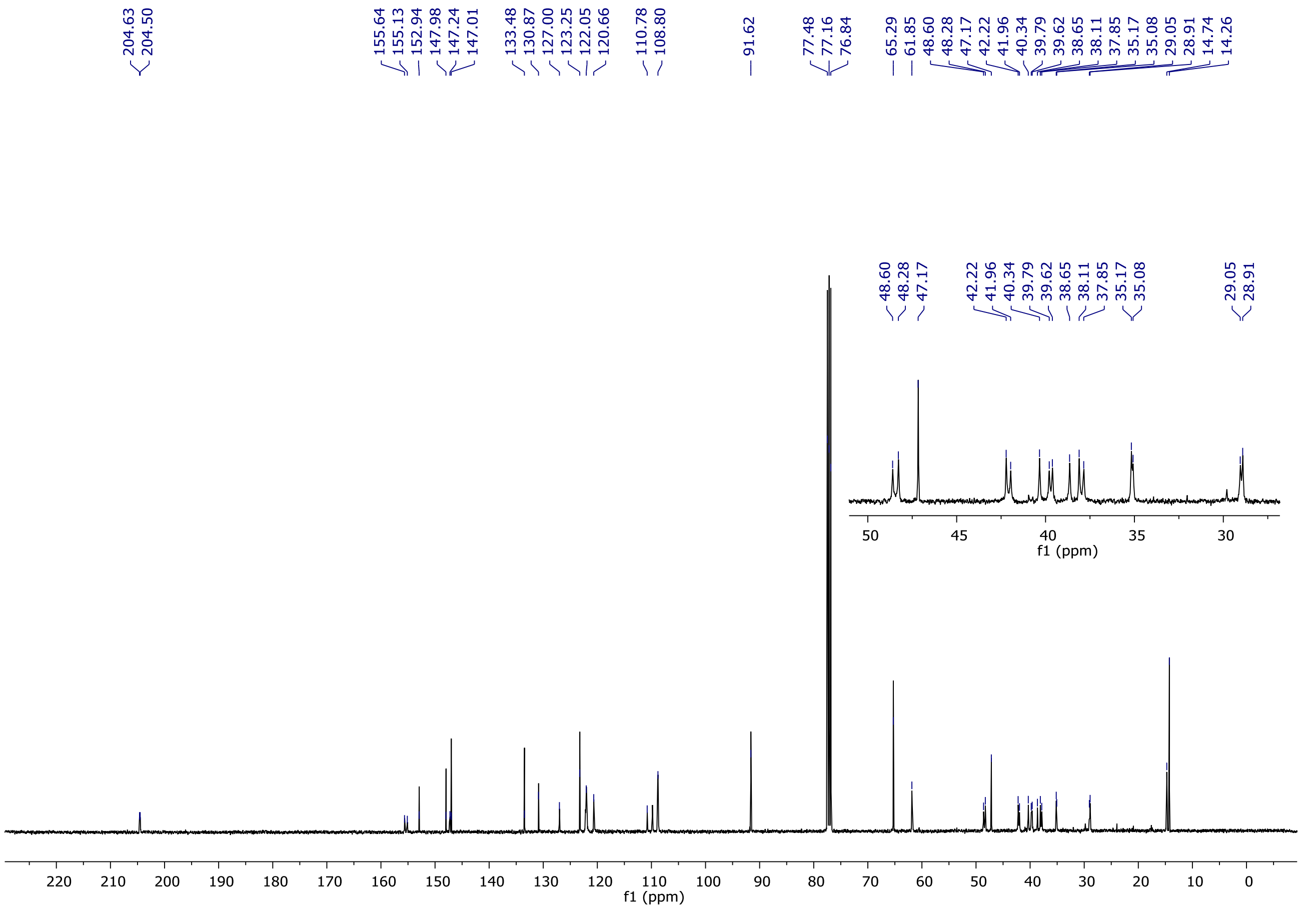

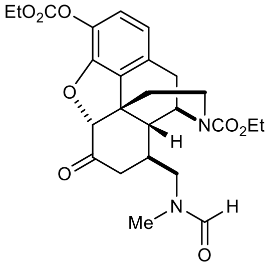
EtO2CO
O
H
NCO2Et
O
Me
N
H
O

2aj

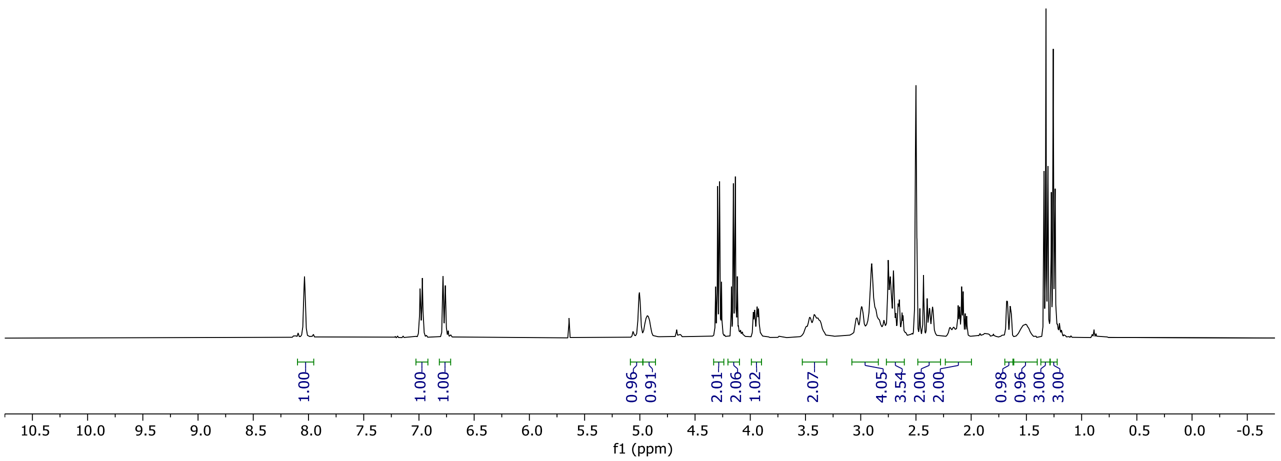
1.00
1.00
1.00
0.96
0.91
2.01
2.06
1.02
2.07
4.05
3.54
2.00
2.00
0.98
0.96
3.00
3.00
10.5 10.0 9.5 9.0 8.5 8.0 7.5 7.0 6.5 6.0 5.5 5.0 4.5 4.0 3.5 3.0 2.5 2.0 1.5 1.0 0.5 0.0 -0.5
f1 (ppm)

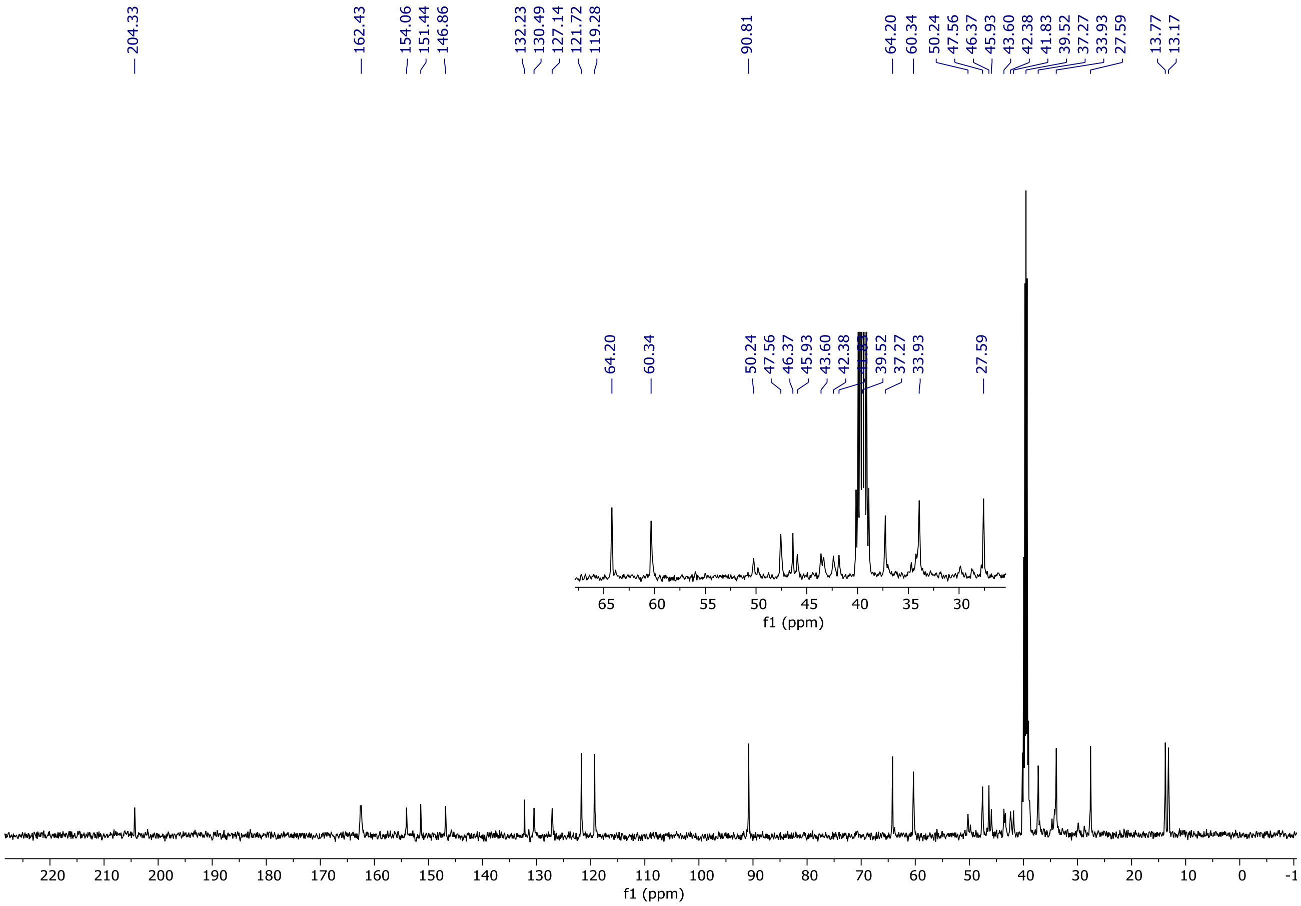
204.33
162.43
154.06
151.44
146.86
132.23
130.49
127.14
121.72
119.28
90.81
64.20
60.34
50.24
47.56
46.37
45.93
43.60
42.38
41.83
39.52
37.27
33.93
27.59
13.77
13.17
65 60 55 50 45 40 35 30
f1 (ppm)
220 210 200 190 180 170 160 150 140 130 120 110 100 90 80 70 60 50 40 30 20 10 0 -1
f1 (ppm)

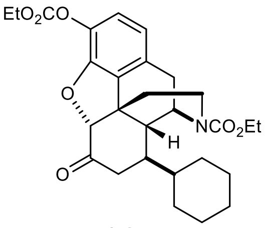

2ak
(1.5 mixture of rotamers)

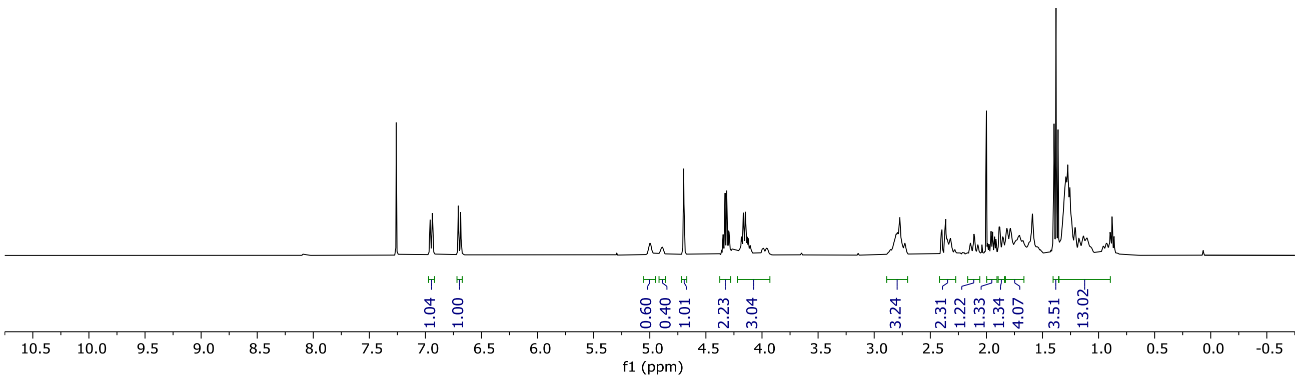

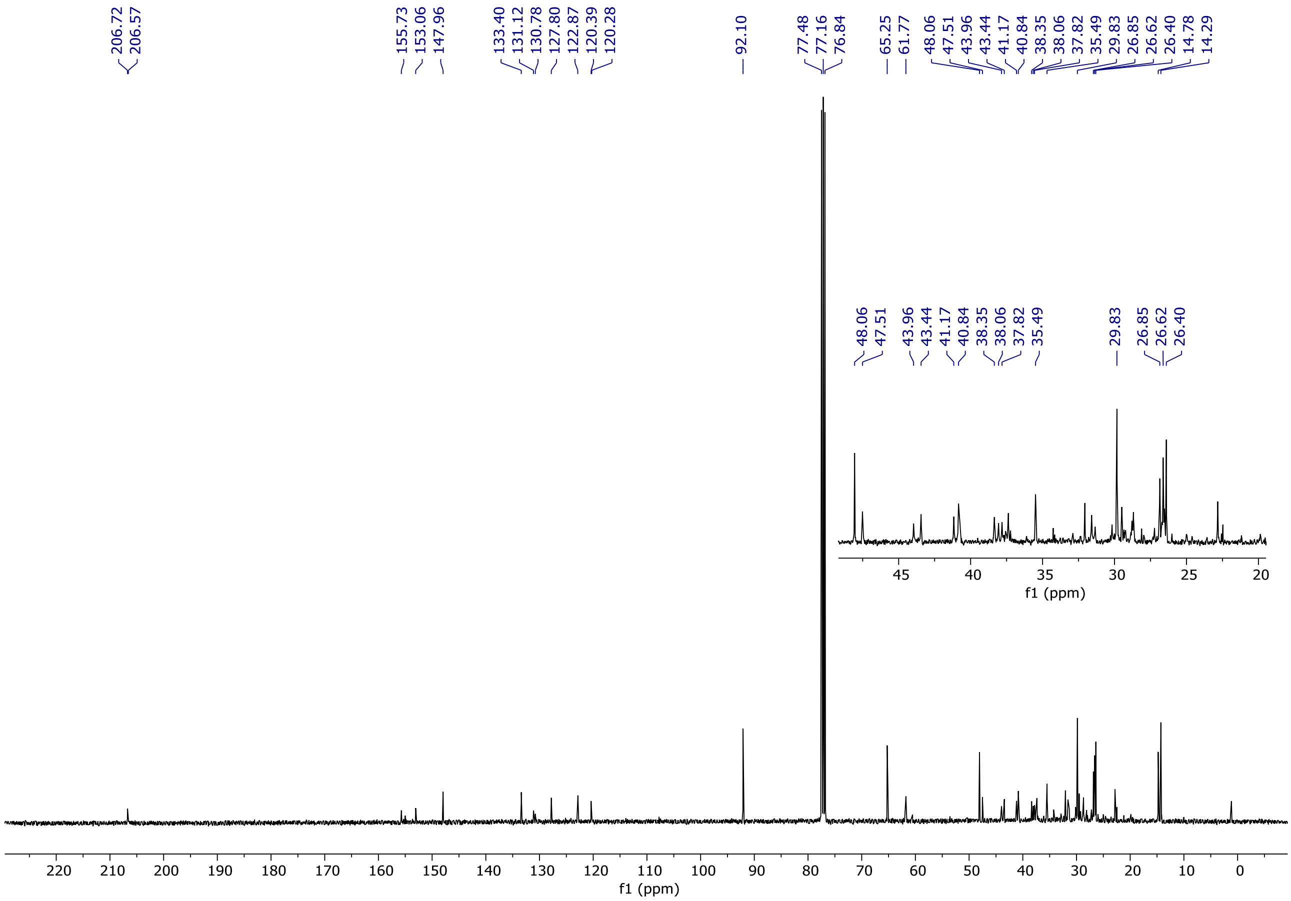

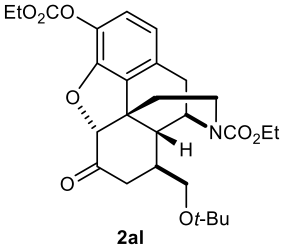

2al
(*ca.* 1:1 mixture of rotamers)

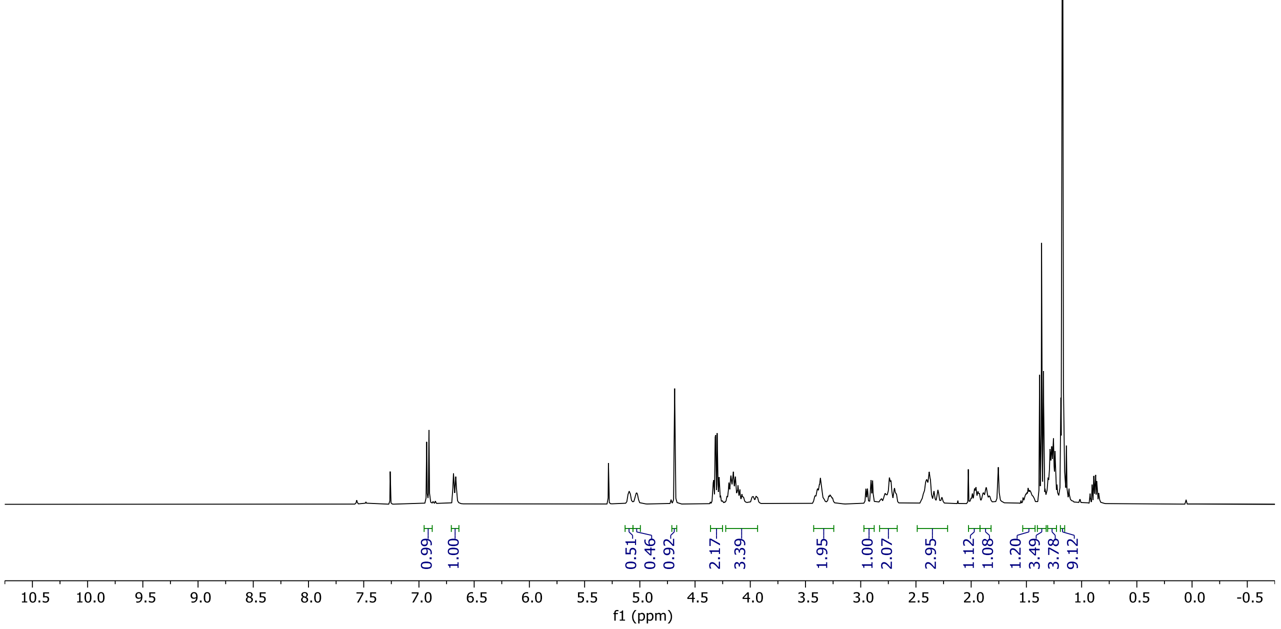

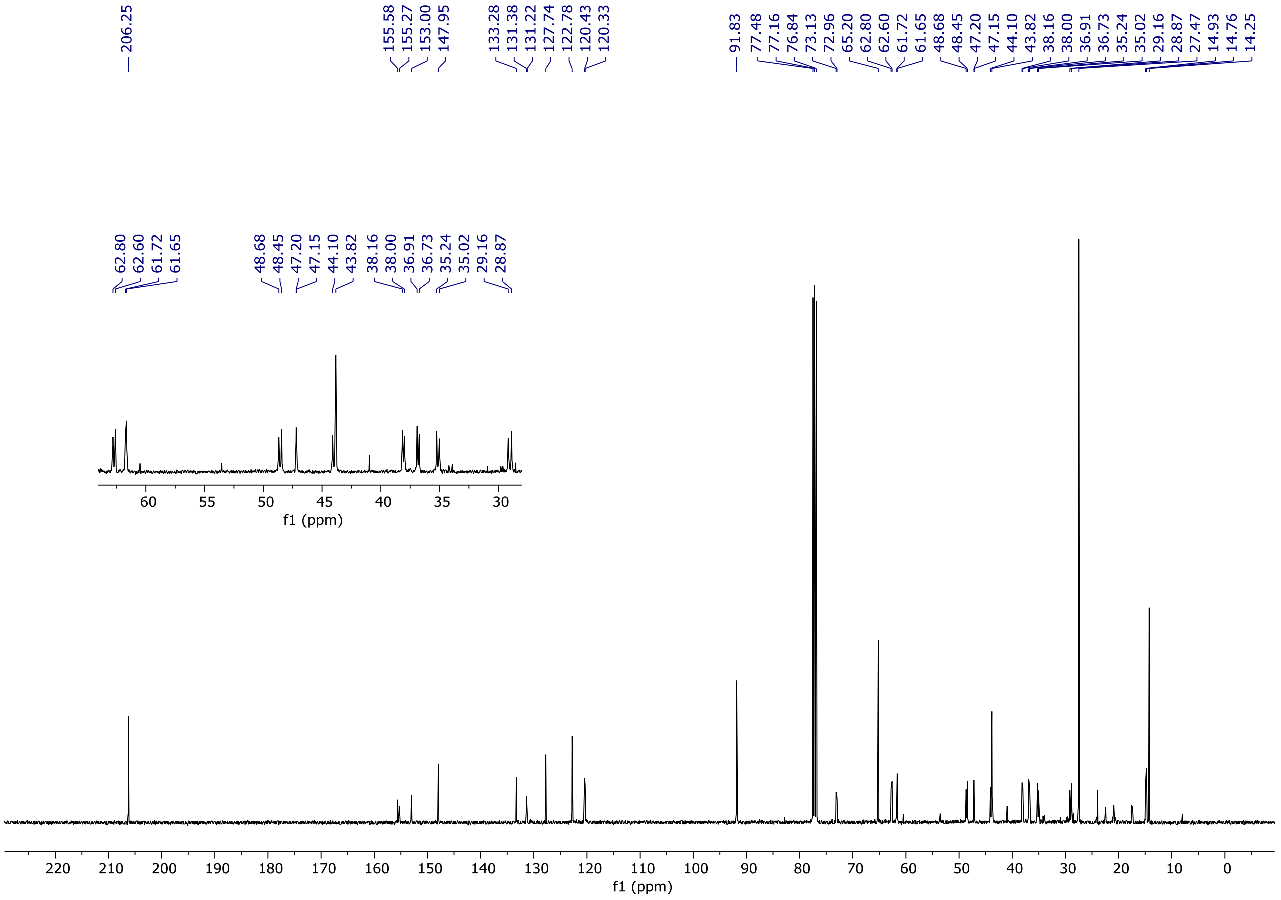

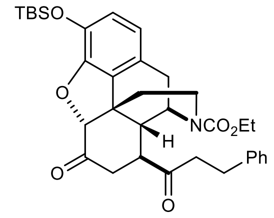

2ba
(*ca.* 1:1 mixture of rotamers)

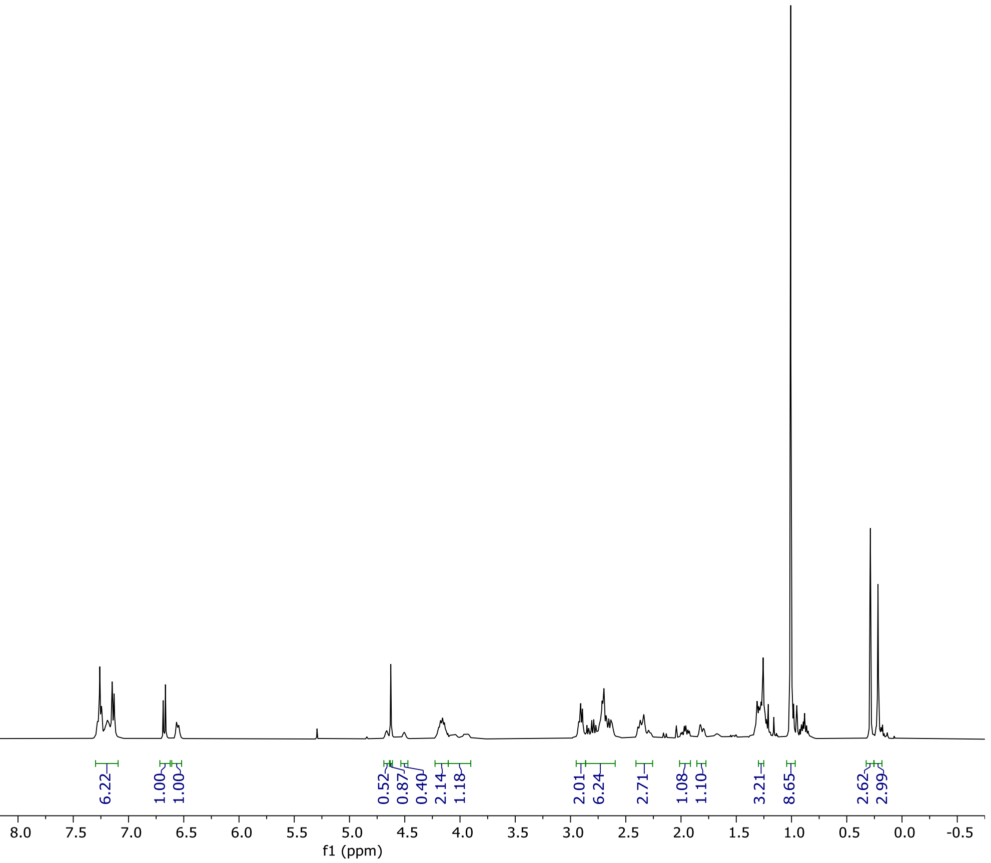

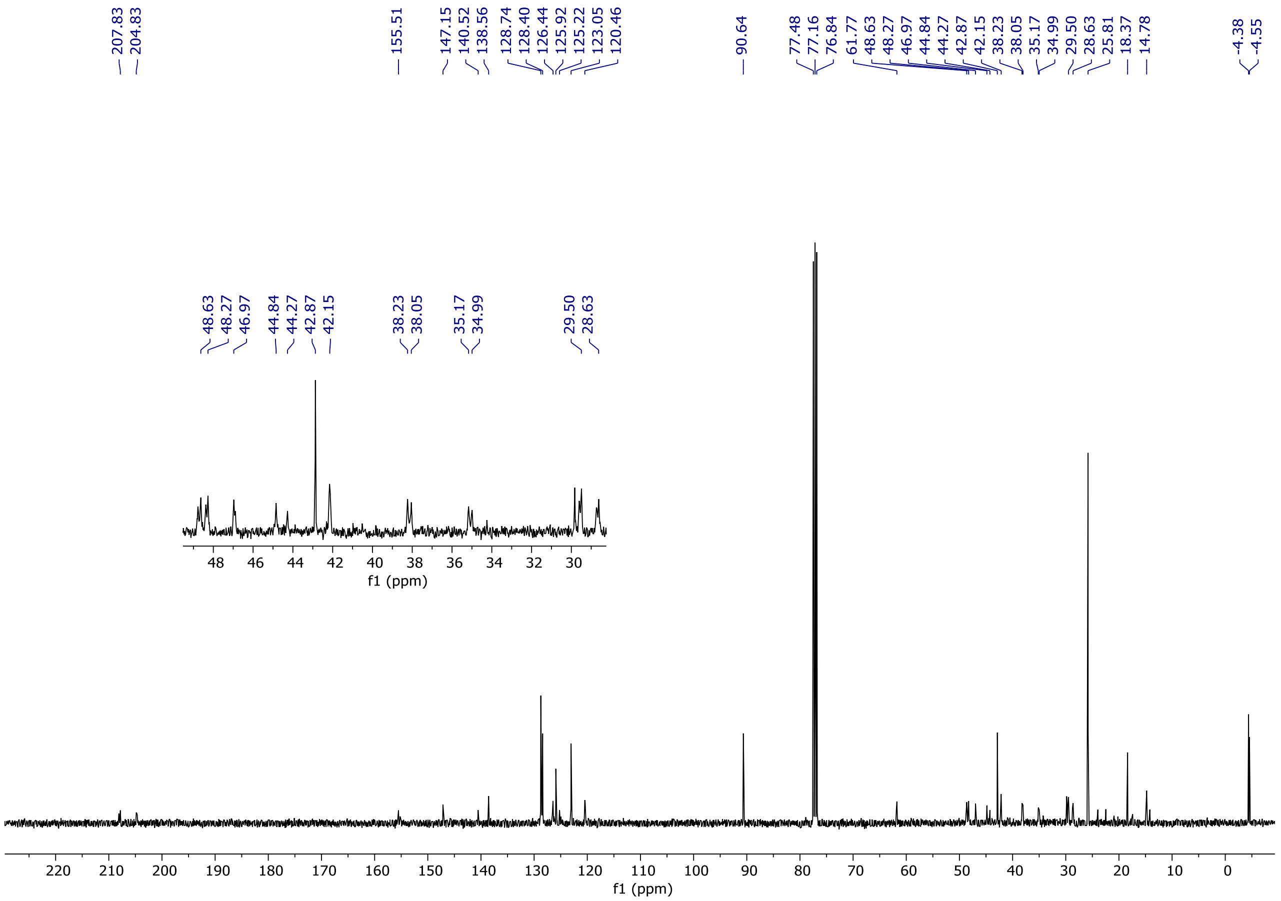

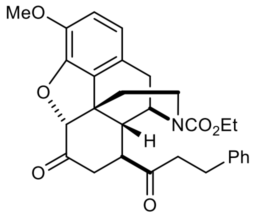

2ca
(*ca.* 1:1 mixture of rotamers)

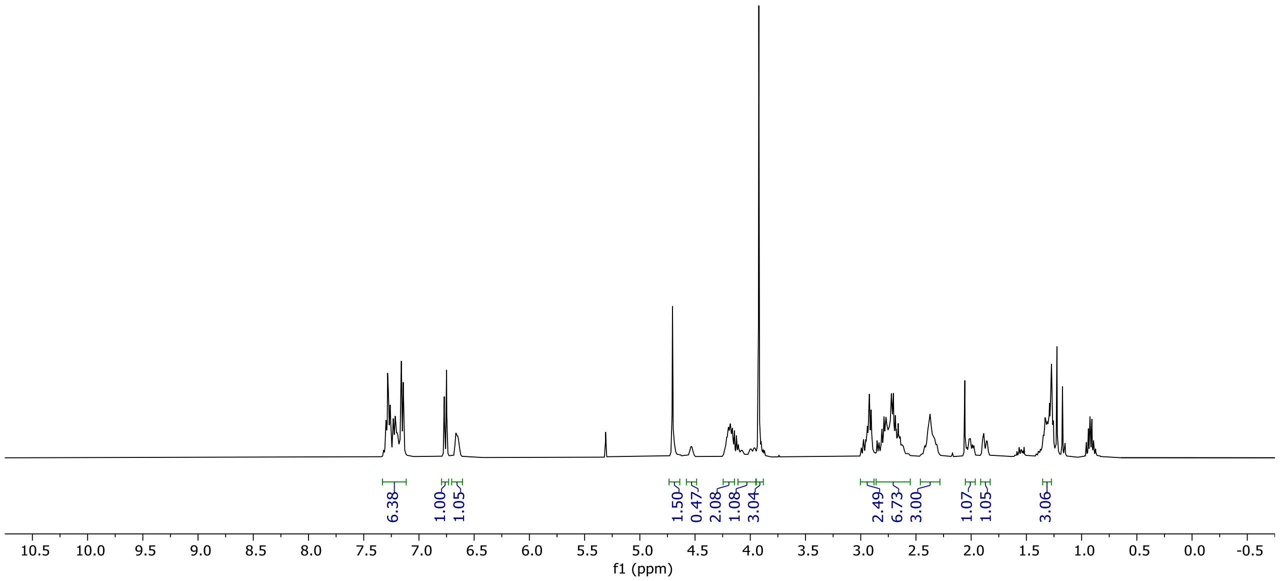

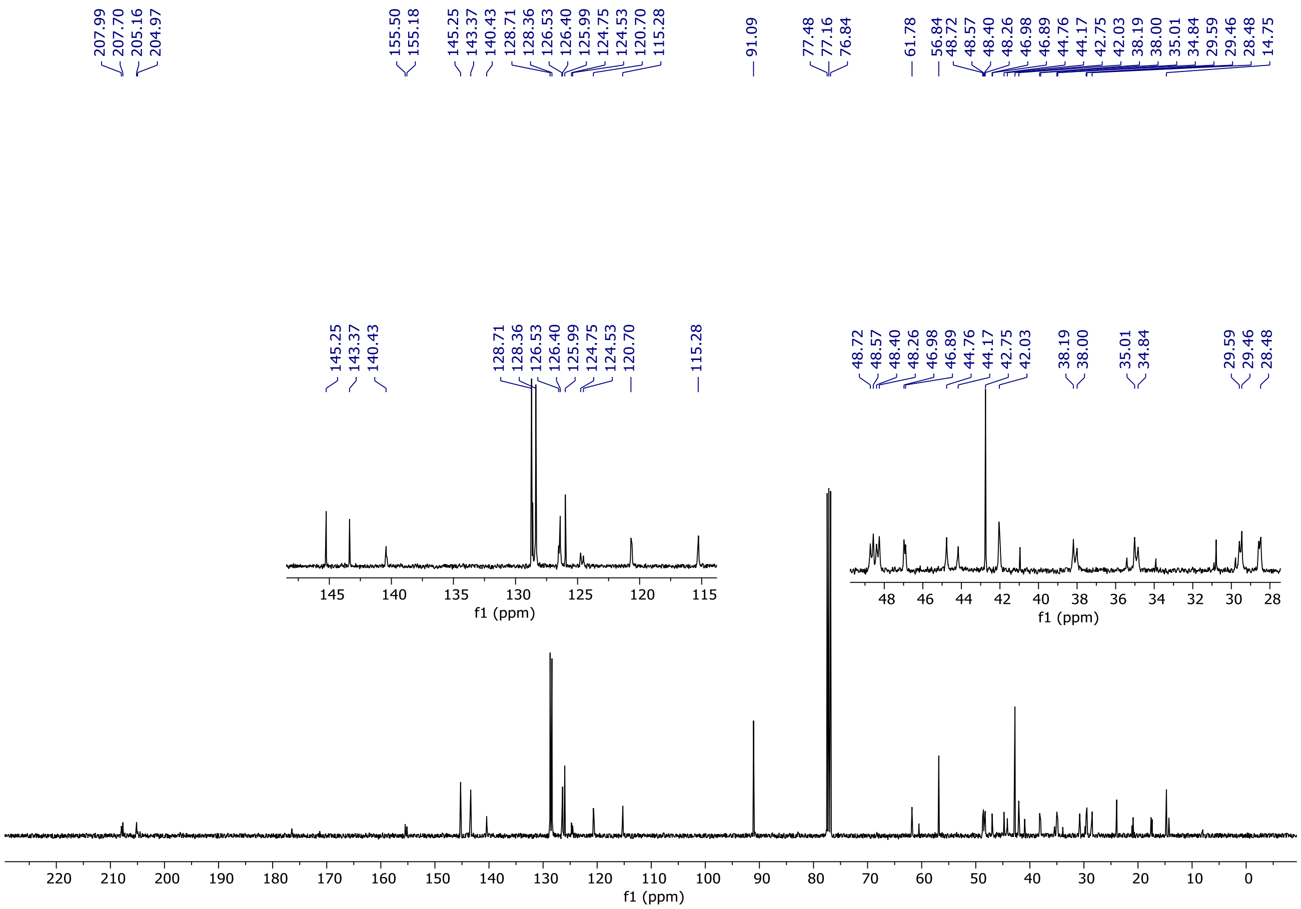

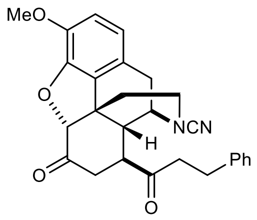
MeO
O
H
NCN
Ph
O
O

2da

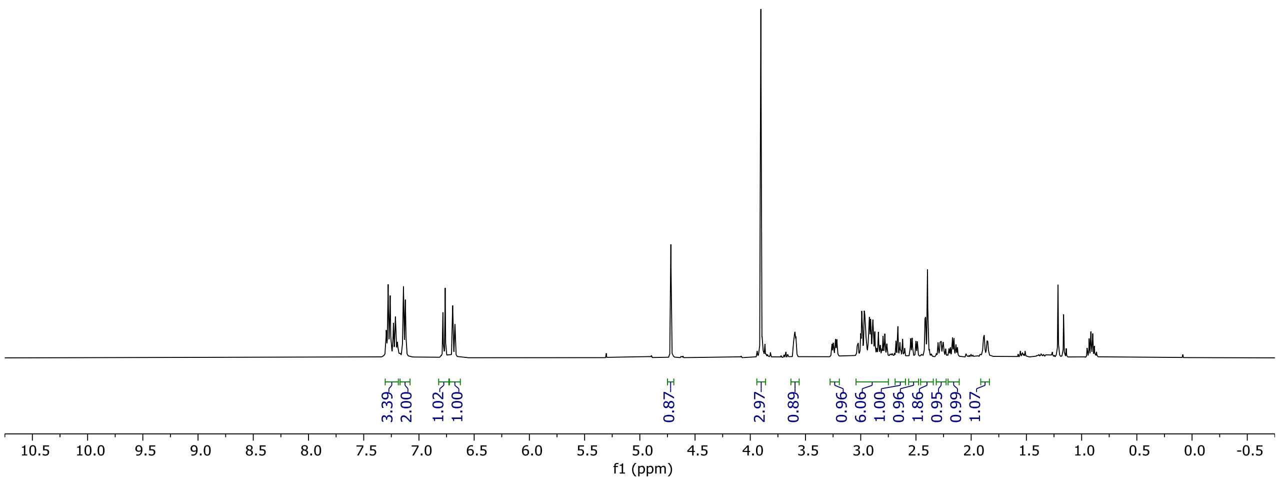
3.39
2.00
1.02
1.00
0.87
2.97
0.89
0.96
6.06
1.00
0.96
1.86
0.95
0.99
1.07
10.5 10.0 9.5 9.0 8.5 8.0 7.5 7.0 6.5 6.0 5.5 5.0 4.5 4.0 3.5 3.0 2.5 2.0 1.5 1.0 0.5 0.0 -0.5
f1 (ppm)

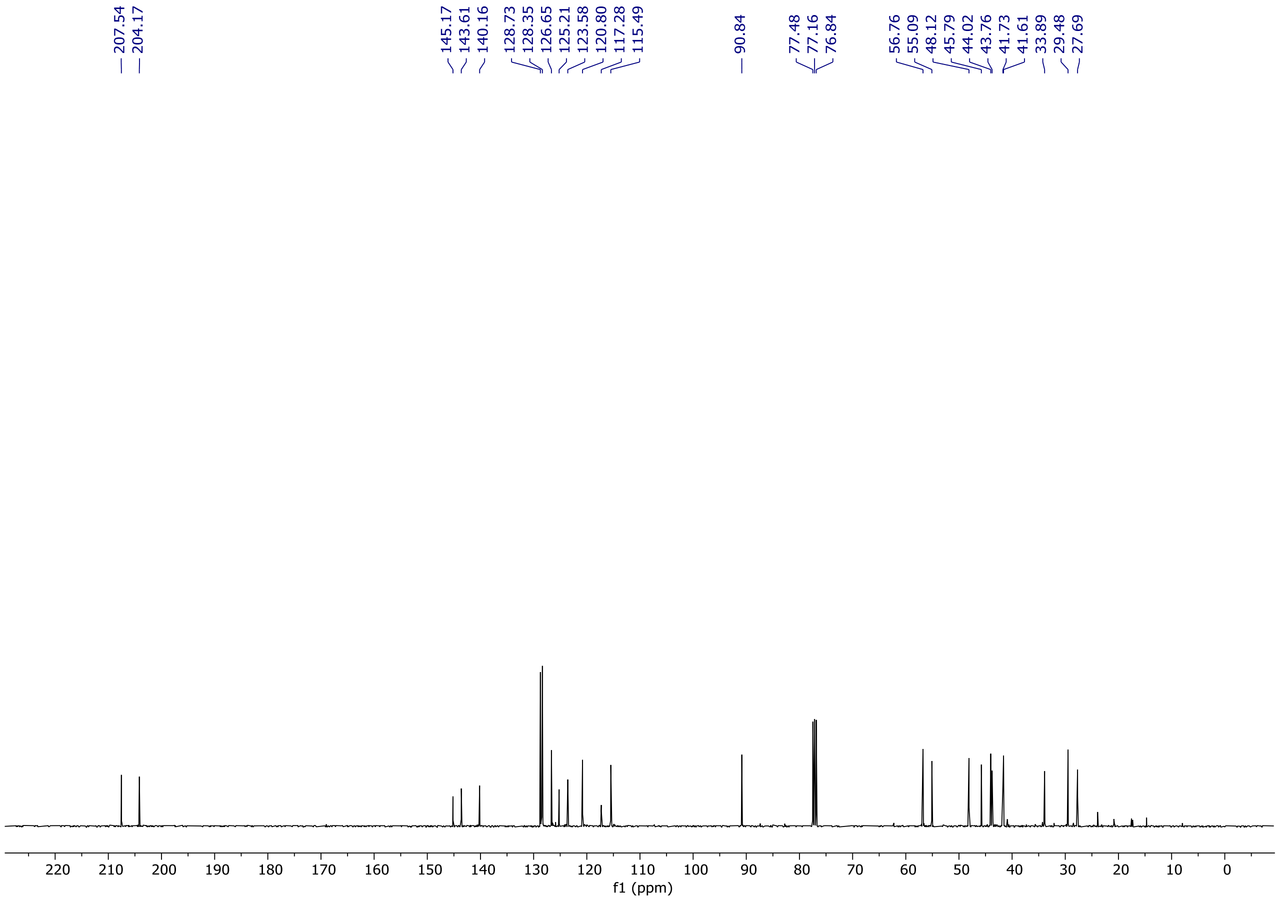
207.54
204.17
145.17
143.61
140.16
128.73
128.35
126.65
125.21
123.58
120.80
117.28
115.49
90.84
77.48
77.16
76.84
56.76
55.09
48.12
45.79
44.02
43.76
41.73
41.61
33.89
29.48
27.69
220 210 200 190 180 170 160 150 140 130 120 110 100 90 80 70 60 50 40 30 20 10 0
f1 (ppm)

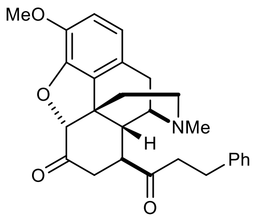
MeO
O
NMe
H
O
O
Ph

2ea

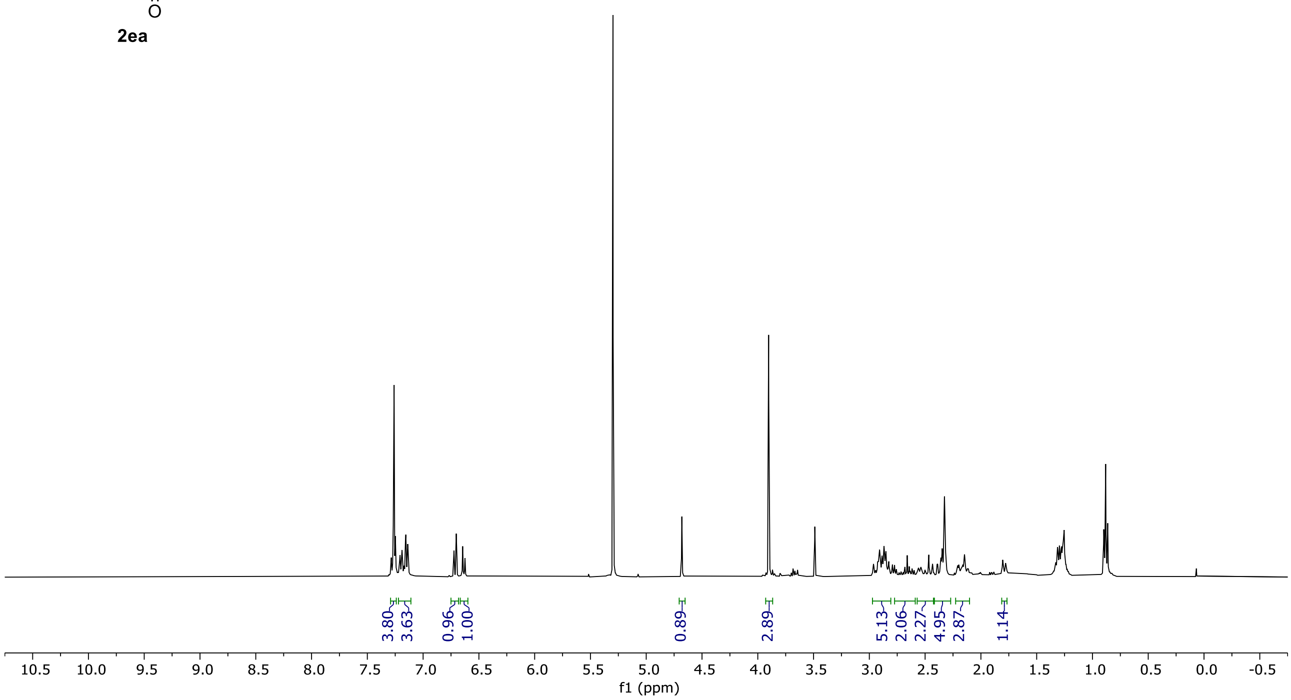
3.80
3.63
0.96
1.00
0.89
2.89
5.13
2.06
2.27
4.95
2.87
1.14
10.5 10.0 9.5 9.0 8.5 8.0 7.5 7.0 6.5 6.0 5.5 5.0 4.5 4.0 3.5 3.0 2.5 2.0 1.5 1.0 0.5 0.0 -0.5
f1 (ppm)

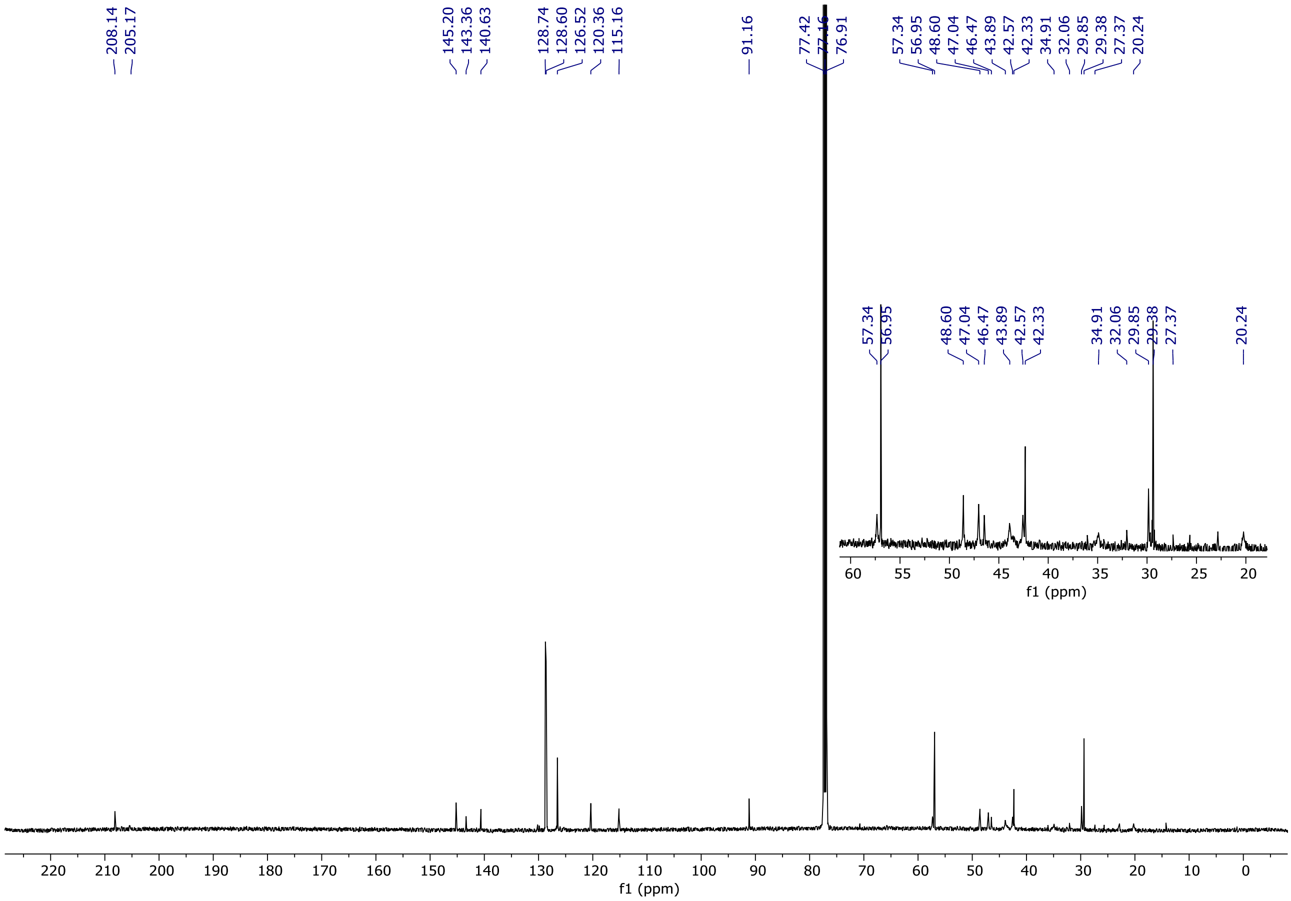
208.14
205.17
145.20
143.36
140.63
128.74
128.60
126.52
120.36
115.16
91.16
77.42
77.16
76.91
57.34
56.95
48.60
47.04
46.47
43.89
42.57
42.33
34.91
32.06
29.85
29.38
27.37
20.24
60 55 50 45 40 35 30 25 20
f1 (ppm)
220 210 200 190 180 170 160 150 140 130 120 110 100 90 80 70 60 50 40 30 20 10 0
f1 (ppm)

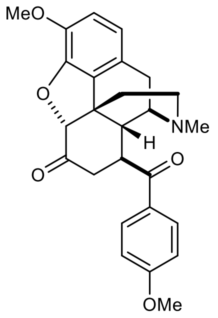
MeO
O
NMe
H
O
O
OMe

2eg

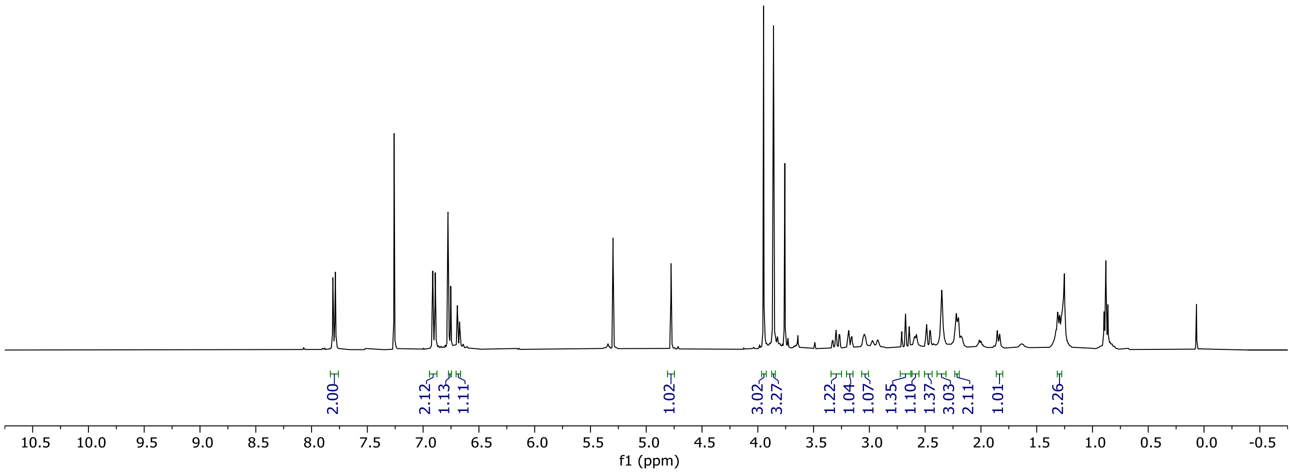
2.00
2.12
1.13
1.11
1.02
3.02
3.27
1.22
1.04
1.07
1.35
1.10
1.37
3.03
2.11
1.01
2.26
10.5 10.0 9.5 9.0 8.5 8.0 7.5 7.0 6.5 6.0 5.5 5.0 4.5 4.0 3.5 3.0 2.5 2.0 1.5 1.0 0.5 0.0 -0.5
f1 (ppm)

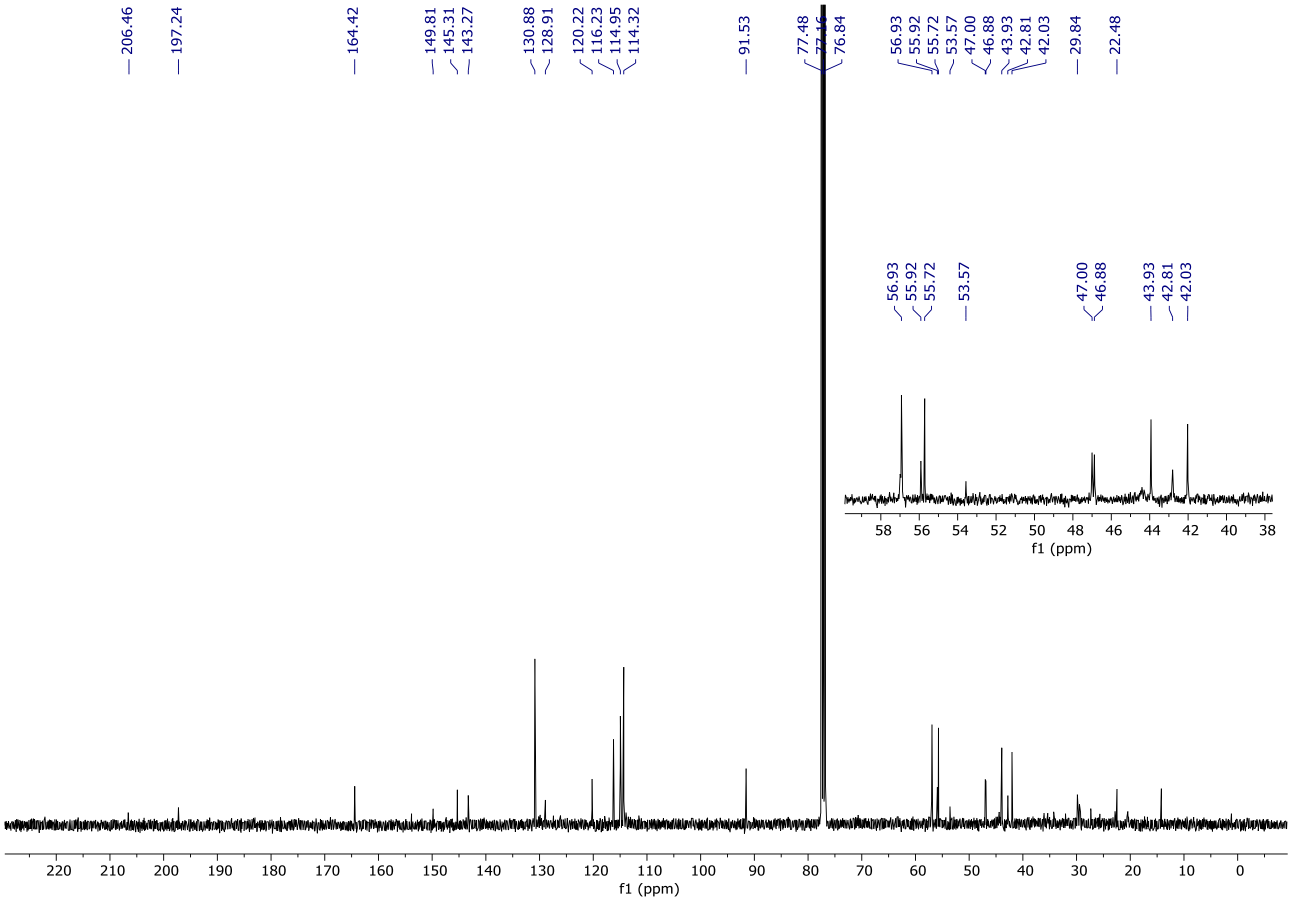
206.46
197.24
164.42
149.81
145.31
143.27
130.88
128.91
120.22
116.23
114.95
114.32
91.53
77.48
76.84
56.93
55.92
55.72
53.57
47.00
46.88
43.93
42.81
42.03
29.84
22.48
56.93
55.92
55.72
53.57
47.00
46.88
43.93
42.81
42.03
58 56 54 52 50 48 46 44 42 40 38
f1 (ppm)
220 210 200 190 180 170 160 150 140 130 120 110 100 90 80 70 60 50 40 30 20 10 0
f1 (ppm)

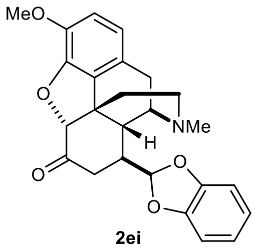
MeO
O
H
NMe
O
O
O

2ei

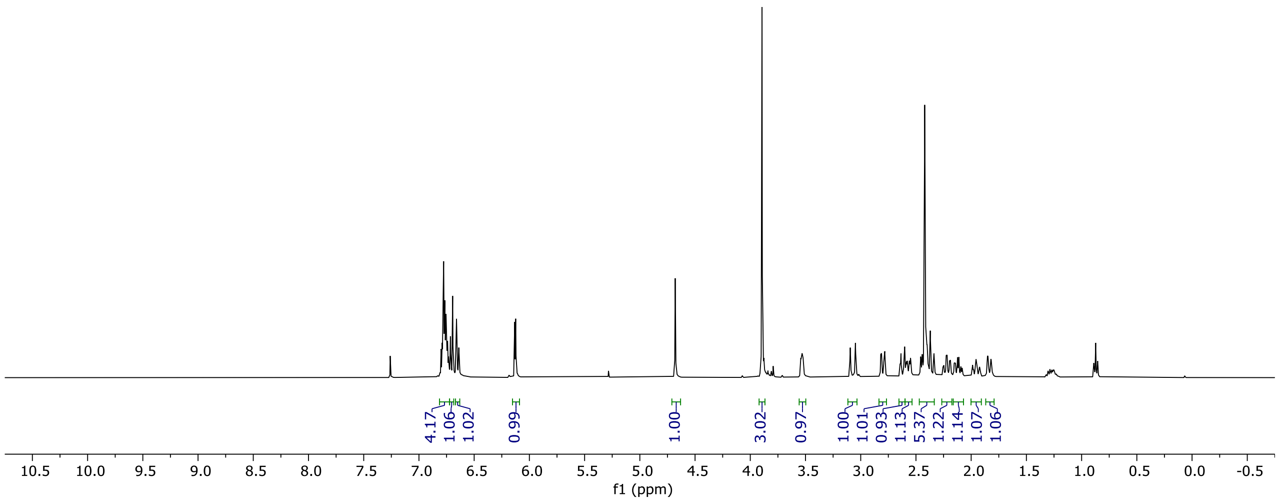
4.17
1.06
1.02
0.99
1.00
3.02
0.97
1.00
1.01
0.93
1.13
5.37
1.22
1.14
1.07
1.06
10.5 10.0 9.5 9.0 8.5 8.0 7.5 7.0 6.5 6.0 5.5 5.0 4.5 4.0 3.5 3.0 2.5 2.0 1.5 1.0 0.5 0.0 -0.5
f1 (ppm)

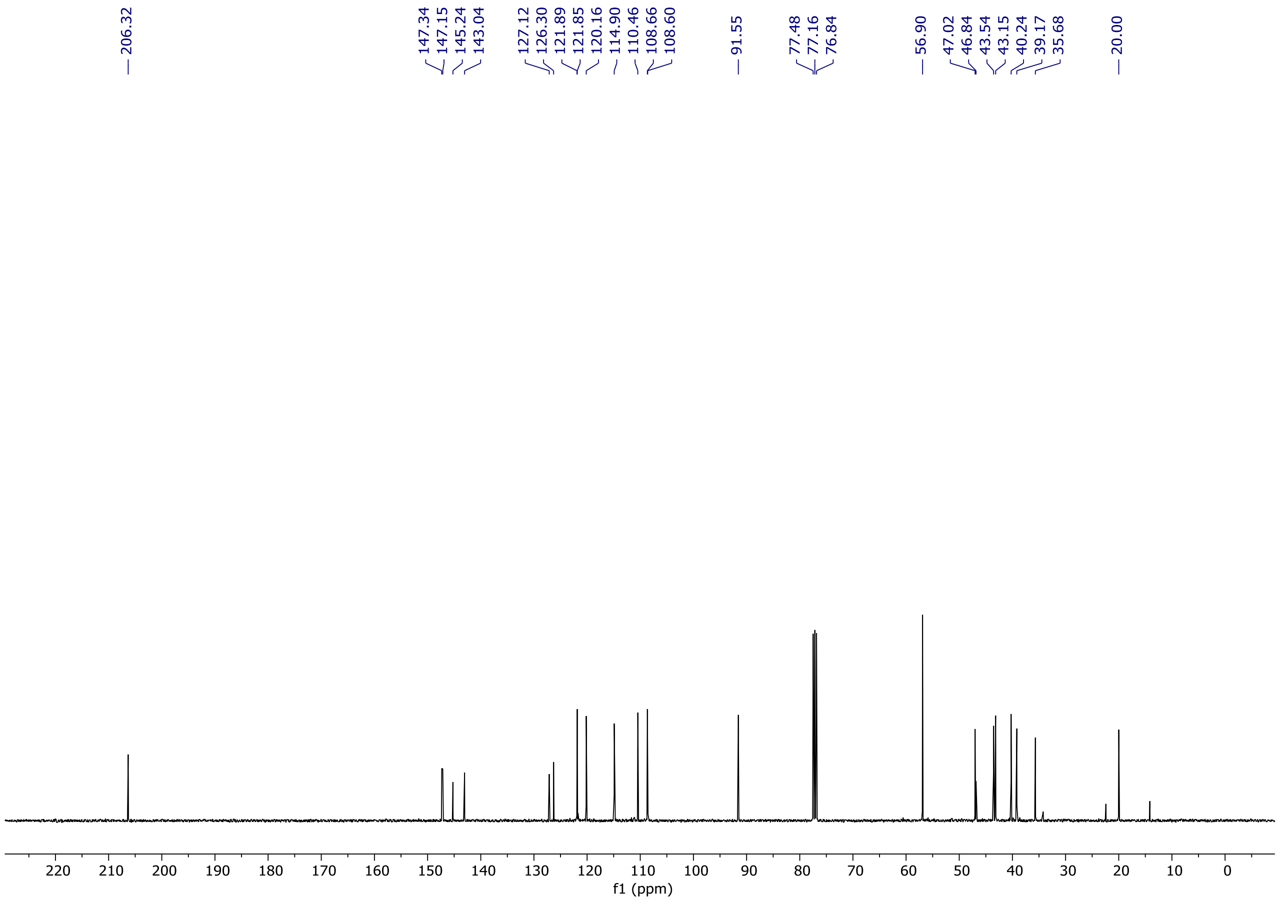
206.32
147.34
147.15
145.24
143.04
127.12
126.30
121.89
121.85
120.16
114.90
110.46
108.66
108.60
91.55
77.48
77.16
76.84
56.90
47.02
46.84
43.54
43.15
40.24
39.17
35.68
20.00
220 210 200 190 180 170 160 150 140 130 120 110 100 90 80 70 60 50 40 30 20 10 0
f1 (ppm)

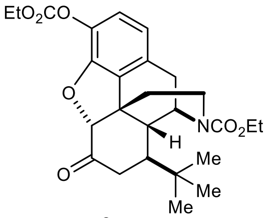

2am
(1.5:1 mixture of rotamers)

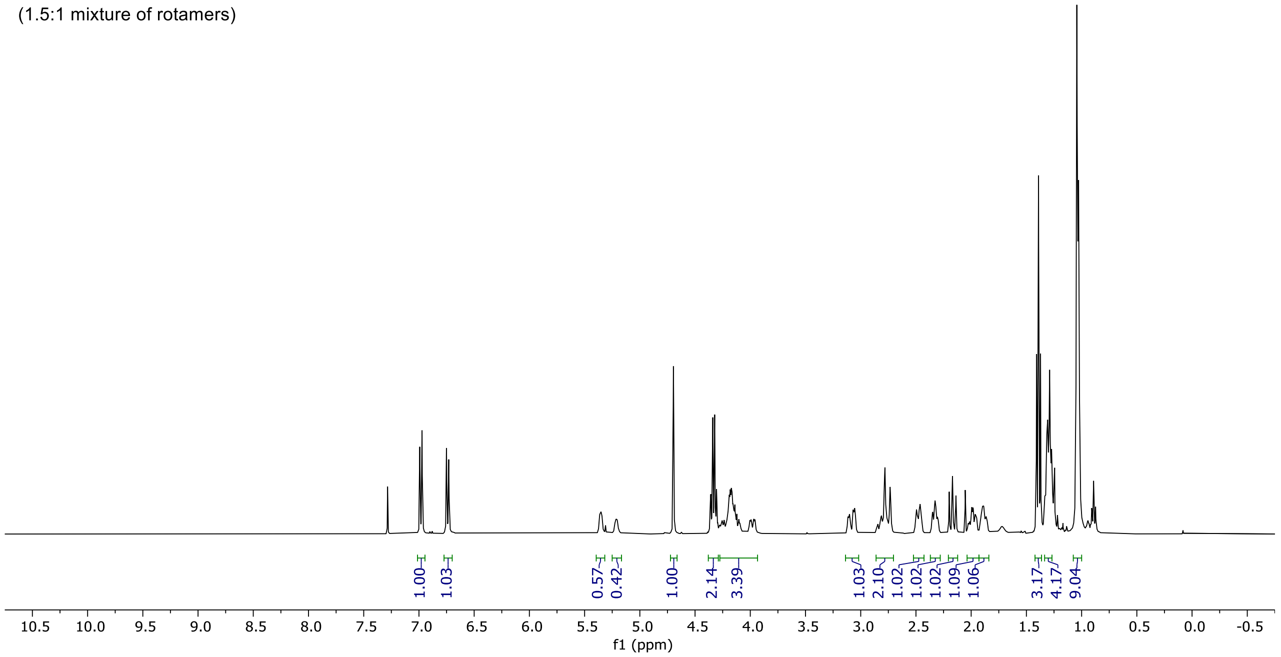

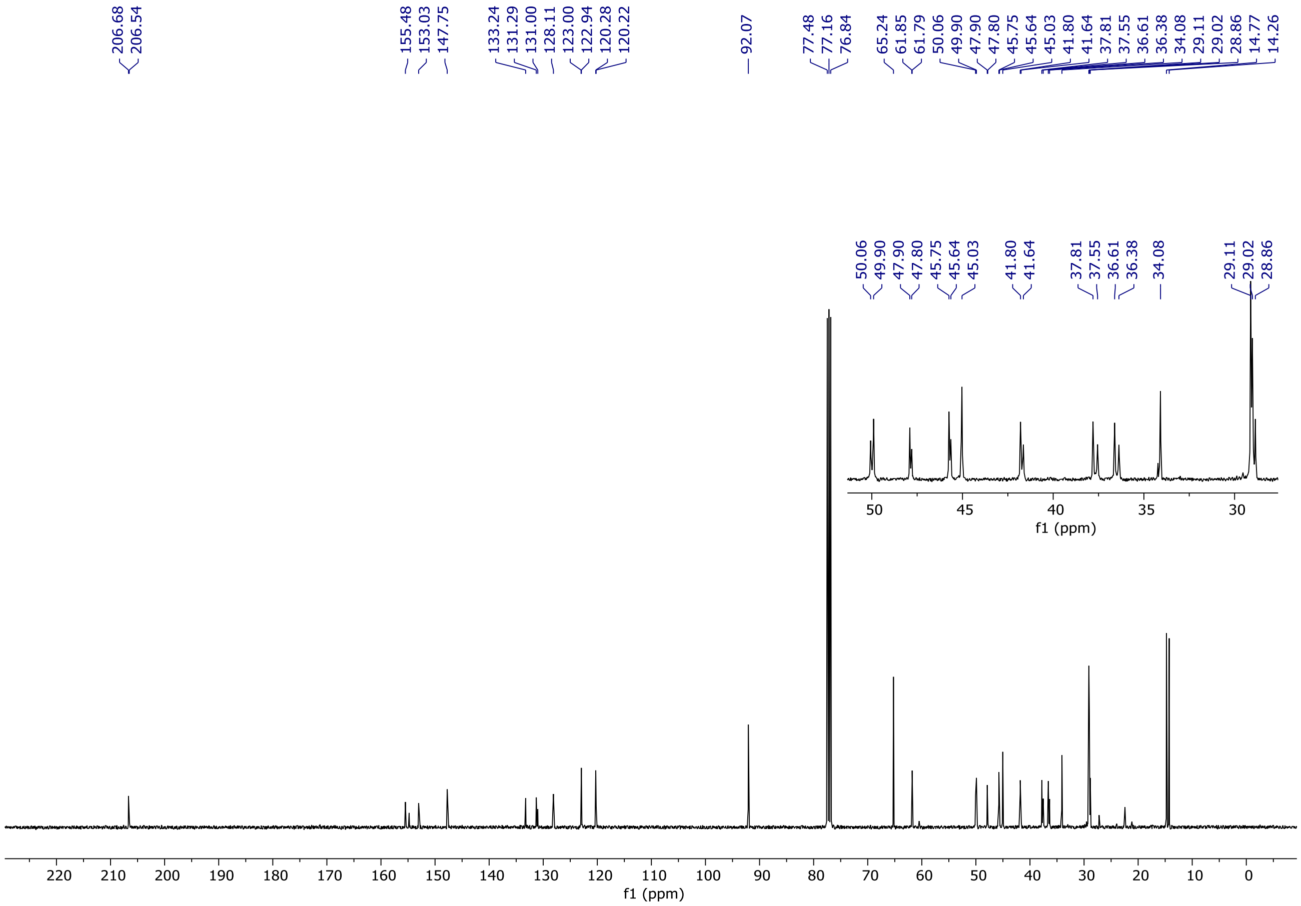

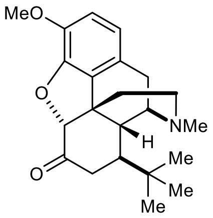
MeO
O
NMe
H
O
Me
Me
Me

2em

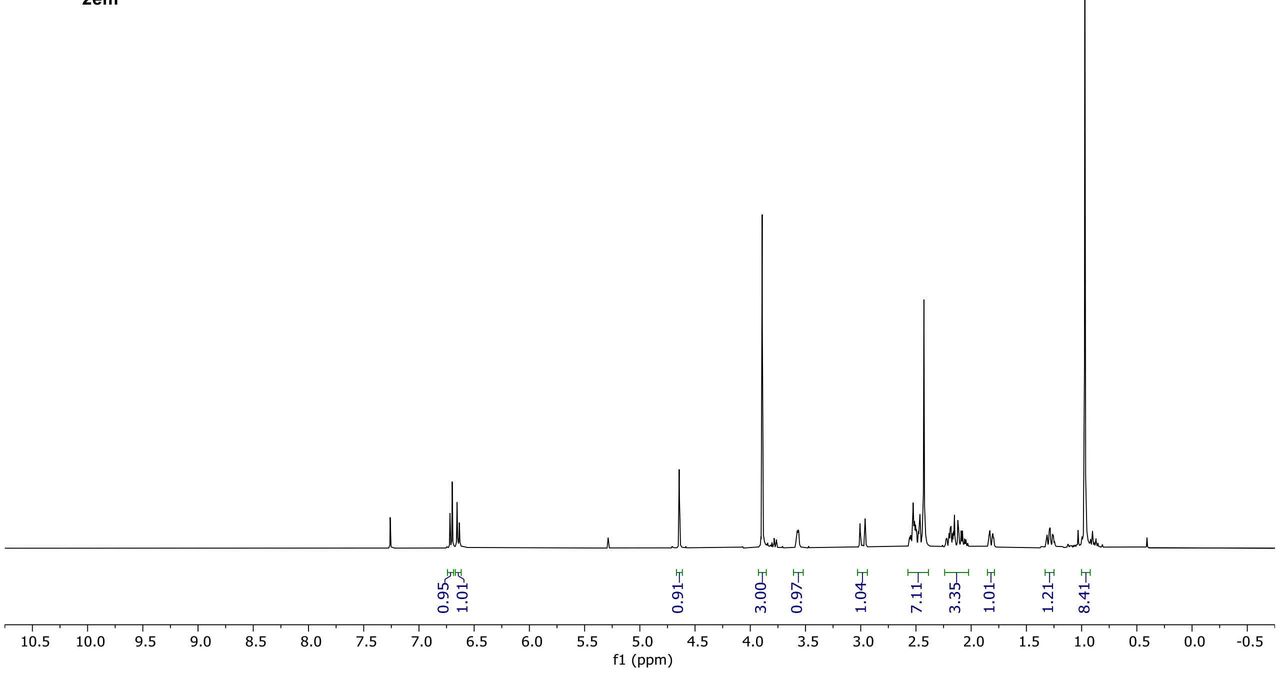
0.95
1.01
0.91
3.00
0.97
1.04
7.11
3.35
1.01
1.21
8.41
10.5 10.0 9.5 9.0 8.5 8.0 7.5 7.0 6.5 6.0 5.5 5.0 4.5 4.0 3.5 3.0 2.5 2.0 1.5 1.0 0.5 0.0 -0.5
f1 (ppm)

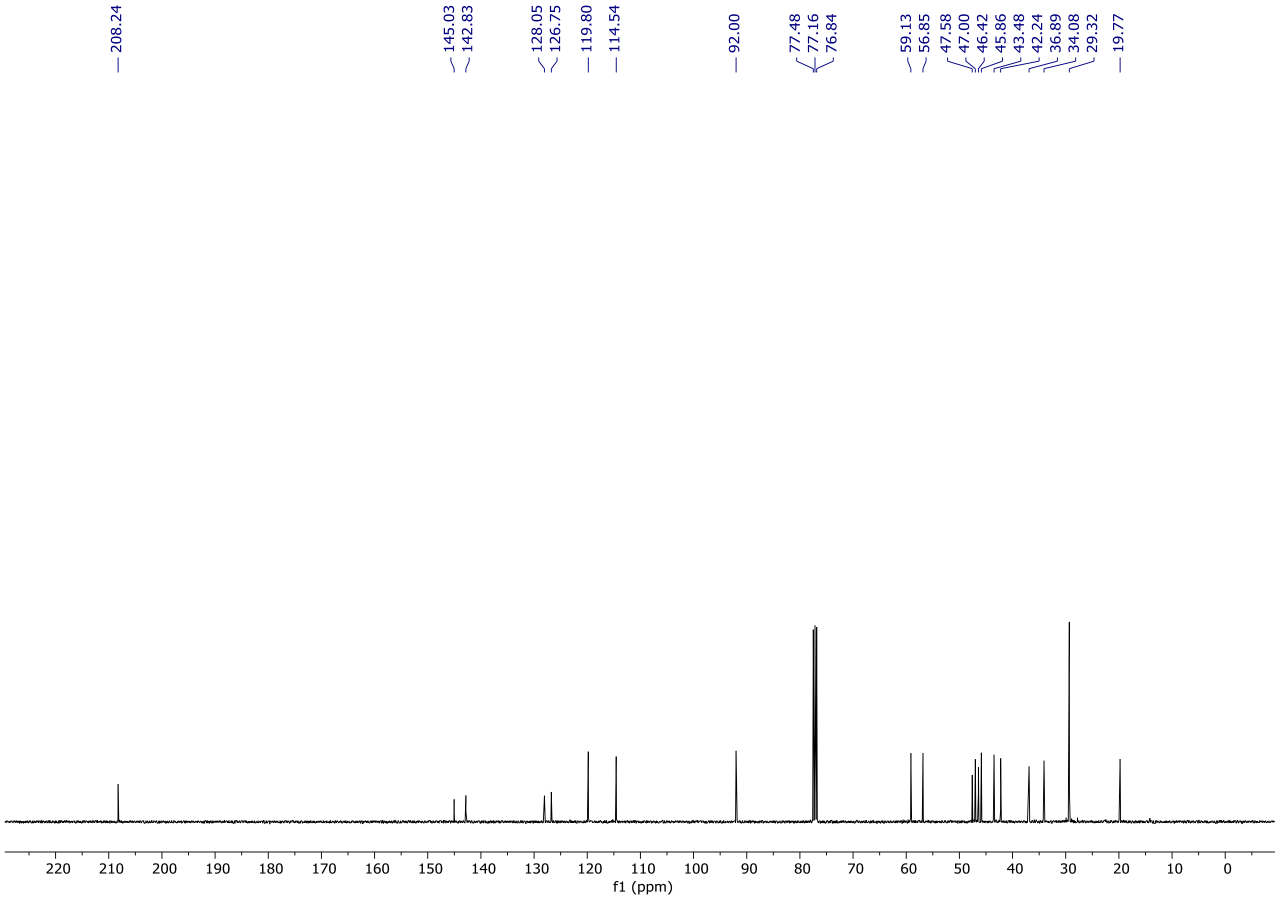
208.24
145.03
142.83
128.05
126.75
119.80
114.54
92.00
77.48
77.16
76.84
59.13
56.85
47.58
47.00
46.42
45.86
43.48
42.24
36.89
34.08
29.32
19.77
220 210 200 190 180 170 160 150 140 130 120 110 100 90 80 70 60 50 40 30 20 10 0
f1 (ppm)

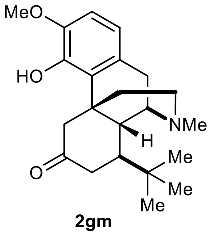
MeO
HO
NMe
H
Me
Me
Me
O

2gm

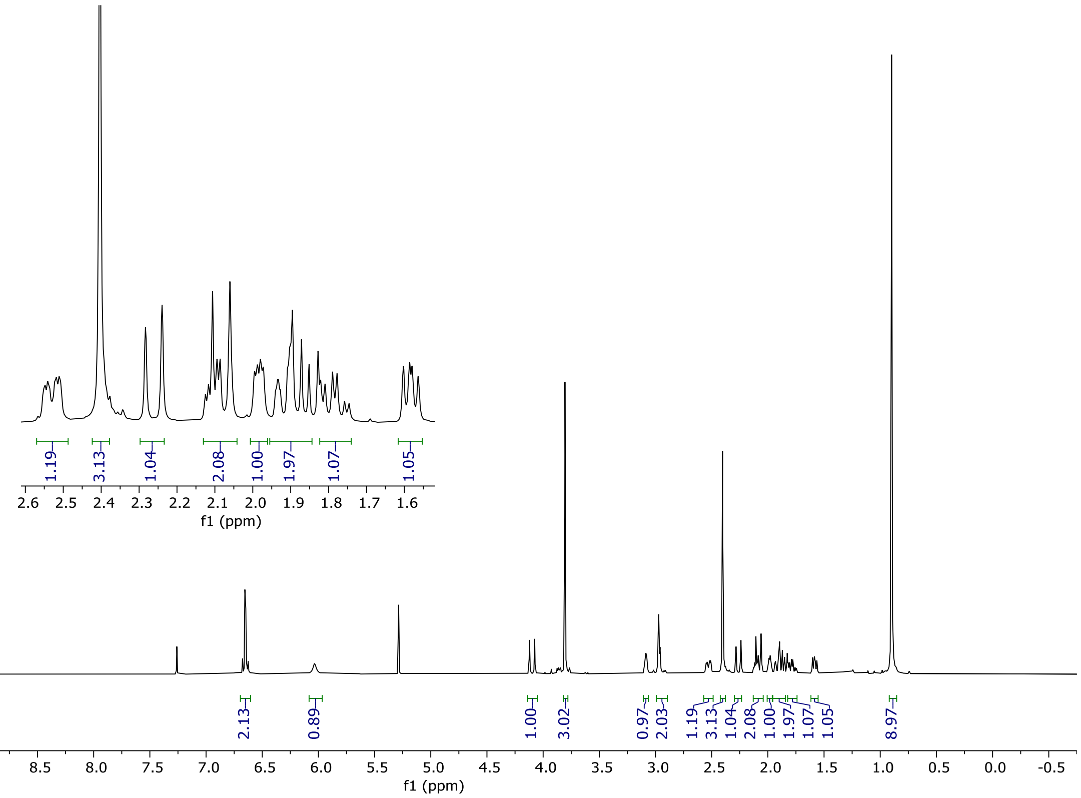
1.19
3.13
1.04
2.08
1.00
1.97
1.07
1.05
2.6 2.5 2.4 2.3 2.2 2.1 2.0 1.9 1.8 1.7 1.6
f1 (ppm)
2.13
0.89
1.00
3.02
0.97
2.03
1.19
3.13
1.04
2.08
1.00
1.97
1.07
1.05
8.97
10.5 10.0 9.5 9.0 8.5 8.0 7.5 7.0 6.5 6.0 5.5 5.0 4.5 4.0 3.5 3.0 2.5 2.0 1.5 1.0 0.5 0.0 -0.5
f1 (ppm)

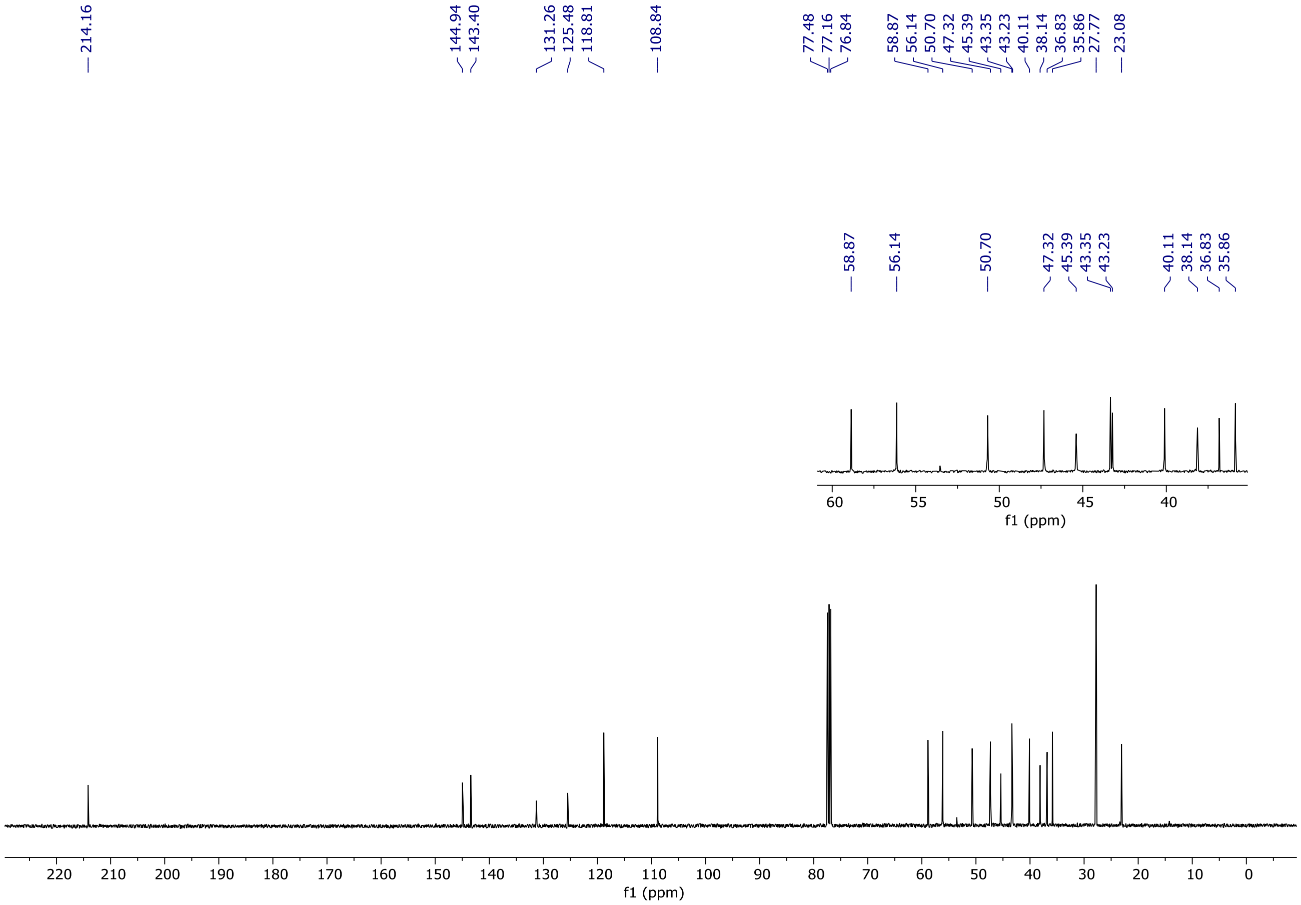
214.16
144.94
143.40
131.26
125.48
118.81
108.84
77.48
77.16
76.84
58.87
56.14
50.70
47.32
45.39
43.35
43.23
40.11
38.14
36.83
35.86
27.77
23.08
60 55 50 45 40
f1 (ppm)
220 210 200 190 180 170 160 150 140 130 120 110 100 90 80 70 60 50 40 30 20 10 0
f1 (ppm)

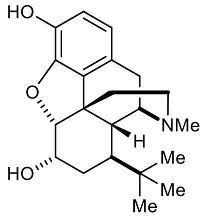
HO
O
NMe
H
Me
Me
HO
Me

3

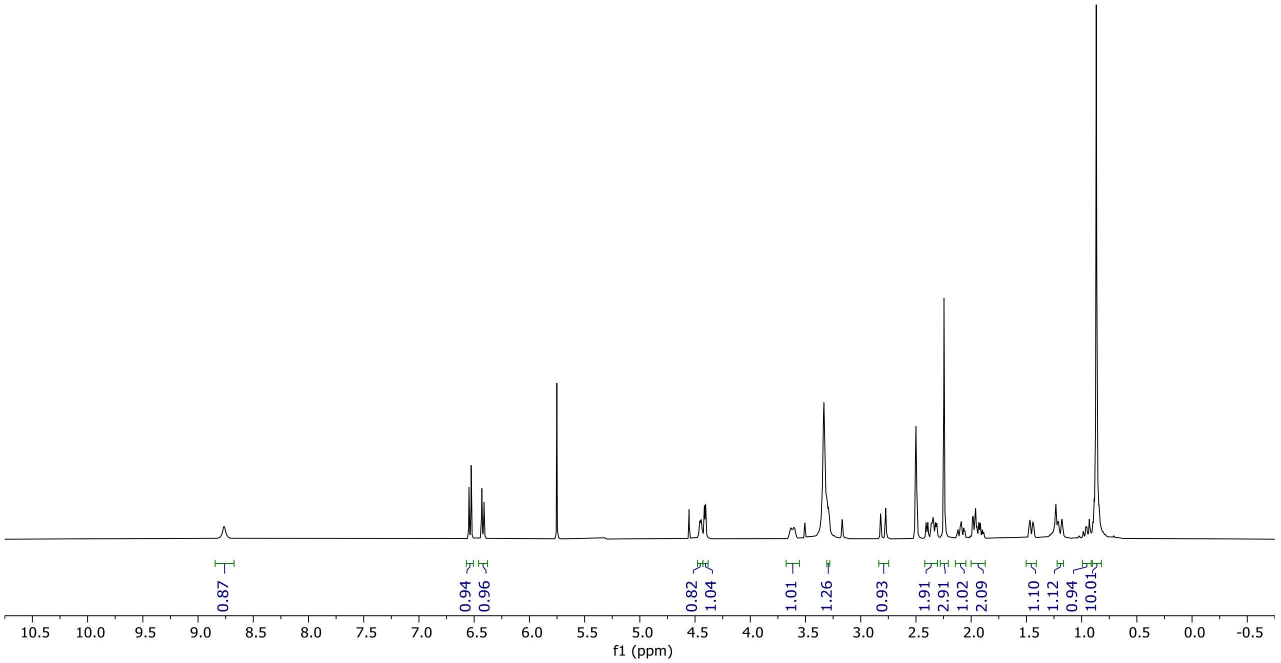
0.87
0.94
0.96
0.82
1.04
1.01
1.26
0.93
1.91
2.91
1.02
2.09
1.10
1.12
0.94
10.01
10.5 10.0 9.5 9.0 8.5 8.0 7.5 7.0 6.5 6.0 5.5 5.0 4.5 4.0 3.5 3.0 2.5 2.0 1.5 1.0 0.5 0.0 -0.5
f1 (ppm)

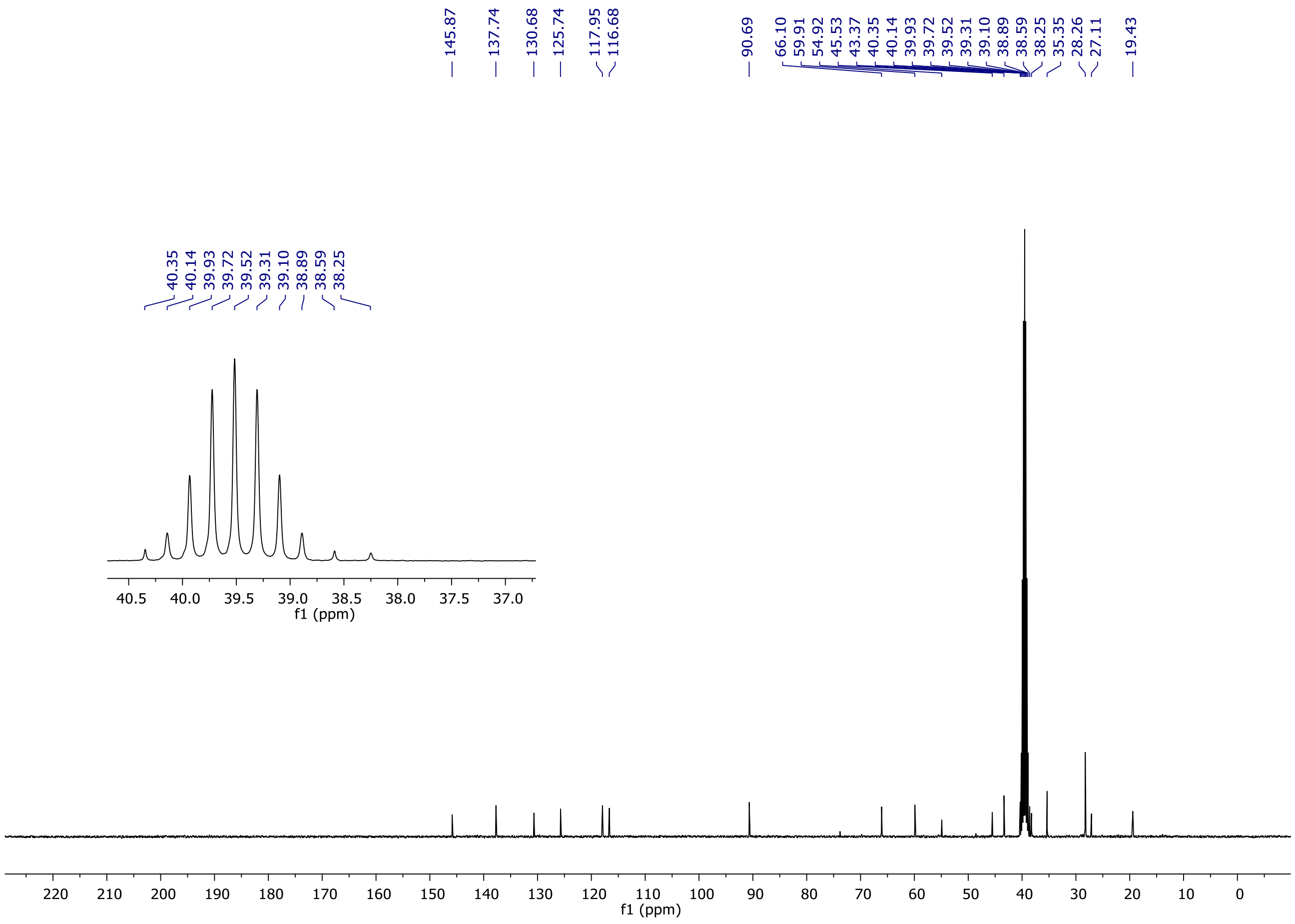
145.87
137.74
130.68
125.74
117.95
116.68
90.69
66.10
59.91
54.92
45.53
43.37
40.35
40.14
39.93
39.72
39.52
39.31
39.10
38.89
38.59
38.25
35.35
28.26
27.11
19.43
40.35
40.14
39.93
39.72
39.52
39.31
39.10
38.89
38.59
38.25
40.5 40.0 39.5 39.0 38.5 38.0 37.5 37.0
f1 (ppm)
220 210 200 190 180 170 160 150 140 130 120 110 100 90 80 70 60 50 40 30 20 10 0
f1 (ppm)

References

1. I. B. Perry, T. F. Brewer, P. J. Sarver, D. M. Schultz, D. A. DiRocco, D. W. C. MacMillan, *Nature* **2018**, *560*, 70-75.
2. R. Gallagher, M. Chebib, T. Balle, M. D. McLeod, *Aust. J. Chem.* **2015**, *68*, 1834-1841.
3. P. Cankař, I. Popa, Z. Trávníček, J. Stýskala, P. Hradil, J. Slouka, *Eur. J. Org. Chem.* **2013**, 6062-6068.
4. X.-R. Huang, S. Srimurugan, G.-H. Lee, C. Chen, *J. Chin. Chem. Soc.* **2011**, *58*, 101-107.
5. A. Zhang, F. Li, C. Ding, Q. Yao, B. I. Knapp, J. M. Bidlack, J. L. Neumeyer, *J. Med. Chem.* **2007**, *50*, 2747-2751.
